# SARS-CoV-2 Infections in a Triad of Primary School Learners (Grades 1-7), Their Parents, and Teachers in KwaZulu-Natal, South Africa: Protocol for a Cross-Sectional and Nested Case-Cohort Study

**DOI:** 10.2196/52713

**Published:** 2024-12-19

**Authors:** Reshmi Dassaye, Terusha Chetty, Brodie Daniels, Zakir Gaffoor, Elizabeth Spooner, Trisha Ramraj, Ncengani Mthethwa, Duduzile Faith Nsibande, Saresha Pillay, Arvin Bhana, Vuyolwethu Magasana, Tarylee Reddy, Khanya Mohlabi, Penelope Linda Moore, Wendy A Burgers, Tulio de Oliveira, Nokukhanya Msomi, Ameena Goga

**Affiliations:** 1 HIV and Other Infectious Diseases Research Unit South African Medical Research Council Cape Town South Africa; 2 Discipline of Public Health Medicine University of KwaZulu-Natal Durban South Africa; 3 Center for Rural Health School of Nursing and Public Health University of KwaZulu-Natal Durban South Africa; 4 Health Systems Research Unit South African Medical Research Council Durban South Africa; 5 Biostatistics Research Unit South African Medical Research Council Durban South Africa; 6 Centre for the AIDS Programme of Research in South Africa Durban South Africa; 7 National Institute for Communicable Diseases of the National Health Laboratory Service Johannesburg South Africa; 8 South African Medical Research Council Antibody Immunity Research Unit, School of Pathology Faculty of Health Sciences University of the Witwatersrand Johannesburg South Africa; 9 Wellcome Centre for Infectious Diseases Research in Africa University of Cape Town Cape Town South Africa; 10 Division of Medical Virology Department of Pathology University of Cape Town Cape Town South Africa; 11 Institute of Infectious Disease and Molecular Medicine University of Cape Town Cape Town South Africa; 12 Centre for Epidemic Response and Innovation School of Data Science and Computational Thinking Stellenbosch University Stellenbosch South Africa; 13 KwaZulu-Natal Research Innovation and Sequencing Platform Nelson R Mandela School of Medicine University of KwaZulu-Natal Durban South Africa; 14 Department of Global Health University of Washington Seattle, WA United States; 15 Discipline of Virology School of Laboratory Medicine and Medical Sciences University of KwaZulu Natal Durban South Africa; 16 National Health Laboratory Service Durban South Africa; 17 Department of Paediatrics and Child Health University of Pretoria Pretoria South Africa

**Keywords:** COVID-19, SARS-CoV-2, learners, seroprevalence, long COVID, transmission dynamics

## Abstract

**Background:**

In low- and middle-income countries (LMICs) such as South Africa, there is paucity of data on SARS-CoV-2 infections among children attending school, including seroprevalence and transmission dynamics.

**Objective:**

This pilot study aims to assess (1) the prevalence of self-reported or confirmed SARS-CoV-2 prior infections, COVID-19 symptoms (including long COVID), seroprevalence of SARS-CoV-2 antibodies, and general/mental health, (2) longitudinal changes in SARS-CoV-2 seroprevalence, and (3) SARS-CoV-2 acute infections, immune responses, transmission dynamics, and symptomatic versus asymptomatic contacts in a unique cohort of unvaccinated primary school learners, their parents, teachers, and close contacts in semirural primary school settings.

**Methods:**

Learners (grades 1-7) from primary schools in KwaZulu-Natal, South Africa, their parents, and teachers will be invited to enroll into the COVID kids school study (CoKiDSS). CoKiDSS comprises 3 parts: a cross-sectional survey (N=640), a follow-up survey (n=300), and a nested case-cohort substudy. Finger-prick blood and saliva samples will be collected for serological and future testing, respectively, in the cross-sectional (451 learners:147 parents:42 teachers) and follow-up (210 learners:70 parents:20 teachers) surveys. The nested case-cohort substudy will include cases from the cross-sectional survey with confirmed current SARS-CoV-2 infection (n=30) and their close contacts (n=up to 10 per infected participant). Finger-prick blood (from all substudy participants), venous blood (from cases), and nasal swabs (from cases and contacts) will be collected for serological testing, immunological testing, and viral genome sequencing, respectively. Questionnaires covering sociodemographic and general and mental health information, prior and current SARS-CoV-2 symptoms and testing information, vaccination status, preventative behavior, and lifestyle will be administered. Statistical methods will include generalized linear mixed models, intracluster correlation, descriptive analysis, and graphical techniques.

**Results:**

A total of 645 participants were enrolled into the cross-sectional survey between May and August 2023. A subset of 300 participants were followed up in the follow-up survey in October 2023. Screening of the participants into the nested case-cohort substudy is planned between November 2023 and September 2024. Data cleanup and analysis for the cross-sectional survey is complete, while those for the follow-up survey and nested case substudy will be completed by the third quarter of 2024. The dissemination and publication of results is anticipated for the fourth quarter of 2024.

**Conclusions:**

This study provides data from an LMIC setting on the impact of SARS-CoV-2 on school-attending learners, their parents, and teachers 3 years after the SARS-CoV-2 pandemic was declared and 21-24 months after resumption of normal school attendance. In particular, this study will provide data on the prevalence of self-reported or confirmed SARS-CoV-2 prior infection, prior and current symptoms, seroprevalence, changes in seroprevalence, SARS-CoV-2 transmission, SARS-CoV-2 adaptive immune responses, and symptoms of long COVID and mental health among a triad of learners, their parents, and teachers.

**International Registered Report Identifier (IRRID):**

DERR1-10.2196/52713

## Introduction

Since the onset of the COVID-19 pandemic, an estimated 75.3 million (21%) children and adolescents <20 years of age have tested positive globally [1]. In South Africa, like many low- and middle-income countries (LMICs), there is a significant population youth bulge: children aged <20 years and children attending primary school make up almost 37% and 28% of the populations, respectively [2]. In their last publicly available report, the National Institute of Communicable Diseases, South Africa, reported that as of December 4, 2021, individuals aged ≤19 years comprised 14.8% of the SARS-CoV-2 tests, 12.5% of laboratory-confirmed COVID-19 cases, 5% of all COVID-19–associated admissions, and 0.7% of COVID-19–associated in-hospital deaths [3]. In most LMICs, children aged <12 years were not offered SARS-CoV-2 vaccines and not prioritized for SARS-CoV-2 testing. In this context, the long-term impact of the COVID-19 pandemic on children requires investigation.

SARS-CoV-2 seroprevalence studies provide an estimate of SARS-CoV-2 antibodies and thus better insight into overall infections, given that access to viral diagnostic testing and testing among mild and asymptomatic cases may be low [4]. In South Africa, during the COVID-19 pandemic, 3 cardinal community studies have reported SARS-CoV-2 seroprevalence among adults and children (Table 1), with an overall increase in the seroprevalence with each successive wave [5-7]. Further, all studies reported that the seroprevalence in adolescents is analogous to that reported in adults, while young children had lower seroprevalence compared to adolescents and adults. Noteworthily, children constituted only a fraction of the sample size in all 3 seroprevalence community surveys (ie, ~55%, 10%, and 20%, respectively) [5-7]. To our knowledge, there is paucity of data reporting the seroprevalence of SARS-CoV-2 among primary school-age children in school settings in South Africa.

**Table 1:** Summary of SARS-CoV-2 seroprevalence studies in South Africa.

Study	Period	Variant of concern	Study setting	Participants (n)	Children and adolescents (n)	Age groups	SARS-CoV-2 infection/seroprevalence among children	SARS-CoV-2 infection/seroprevalence among adults
Cohen et al [5], 2022	July 2020 to August 2021	Beta and Delta variants	Mpumalanga and North West (2 provinces)	1200	664	<5 years of age (n=154, 12.8%) 5-12 years of age (n=340, 28.3%) 13-18 years of age (n=170, 14.2%) 19-39 years of age (n=265, 22.1%) 40-59 years of age (n=168, 14%) ≥50 years of age (n=103, 8.6%)	49% (75/154) in <5 years of age^a^ 60% (205/340) in 5-12 years of age^a^ 78% (132/170) in 13-18 years of age^a^	62.3% (165/265) among 19-39 years of age^b^ 68.5% (115/168) among 40-59 years of age^b^ 55.3% (57/103) among ≥50 years of age^b^
National household-based population seroprevalence survey of SARS-CoV-2 antibodies report [6]	November 2020 to February 2021 and April 2021 to June 2021	Beta and Delta variants	Western Cape, Eastern Cape, Northern Cape, Free State, Kwazulu-Natal, Northwest, Gauteng, Mpumalanga, and Limpopo (9 provinces in South Africa)	13,212	1363	Children<12 years not included 12-17 years of age (n=1363, 10.3%) 18-35 years of age (n=4494, 34%) 36-49 years of age (n=3060, 23.2%) >50 years of age (n=4294, 32.5%)	23.2%, (95% CI 19.2-27.8), 12-17 years of age^c^	17.3% (95% CI 15.0-19.9) among 18-35 years of age^d^ 20.1% (95% CI 17.6-22.8) among 36-49 years of age^d^ 21.3% (95% CI 19.1-23.7) among >50 years of age^d^
Madhi et al [7], 2022	October 2021 to December 9, 2021	Omicron	Gauteng (1 province)	7010	1375	<12 years of age (n=753, 10.7%) 12-17 years of age (n=622, 8.9%) 18-50 years of age (n=4047, 57.7%) >50 years of age (n=1588, 22.7%)	56.2% (95% CI 52.5-59.7) among <12 years of age^c^ 73.8% (95% CI 70.2-77.1) among 12-17 years of age^c^	73.6% (95% CI 72.2-74.9) among 18-50 years of age^d^ 79.7% (95% CI 77.6-81.5) among >50 years of age^d^

^a^SARS-CoV-2 infection among children.

^b^SARS-CoV-2 infection among adults.

^c^SARS-CoV-2 seroprevalence among children.

^d^SARS-CoV-2 seroprevalence among adults.

Initial evidence suggested that children and adolescents are susceptible to infections with ancestral SARS-CoV-2 but at a reduced risk of severe illness or death relative to adults [8]. However, the subsequent Delta and Omicron variants have been more infectious in children than previous variants [9]. A retrospective study in China (n=2135) demonstrated that up to 90% of the pediatric cases were asymptomatic, mild, or moderate [10]. The majority of children hospitalized with severe COVID-19 were unvaccinated or had additional comorbidities such as type 2 diabetes or obesity [11]. A study in the United Kingdom reported that children with underlying neurodisabilities or multiple comorbidities are vulnerable to hospital admission or death [12]. Similar to adults, children and adolescents can also experience long COVID, the frequency and characteristics of which are still under investigation [13].

Studies have focused on differentiating the SARS-CoV-2 immune response between adults and children. In response to SARS-CoV-2 exposure or infection, children elicit a stronger mucosal innate immune response, which facilitates viral clearance [14-22], a lower level of neutrophilia that has previously been associated with microangiopathy and thrombosis [18,20], and a difference in cytokine profiling with a reduced tendency to trigger a cytokine storm [18,19,21-24]. With reference to adaptive immunity, higher lymphocyte counts with a higher proportion of naïve T cells, T regulatory cells, and T follicular helper cells have been reported [24-27]. Additionally, there are conflicting data on mucosal and serum antibody levels reported in children [18-20,24,28-32], and there are discordant reports on the durability and sustainability of the nucleocapsid antibody response in children after infection [33-35]. The Texas Coronavirus Antibody Response ongoing survey reported that 95% of the previously infected children of 5-19 years of age tested positive for nucleocapsid antibodies at the onset of the study and continued to have nucleocapsid antibodies up to 6 months later [36]. SARS-CoV-2 variants have evolving mechanisms to evade host immune defenses; intermittent testing to understand immune response in children exposed to or infected with SARS-CoV-2 infection may contribute to providing key insights into the pathogenesis of severe COVID-19.

During the COVID-19 pandemic, decisions on school closures varied widely between and within countries [37]. Children play a critical role in the transmission of respiratory viruses such as influenza, and school closures were partly guided by such evidence [38,39]. However, the epidemiological benefits of school closures on the transmission of SARS-CoV-2 remain elusive. There are conflicting data on the transmission of SARS-CoV-2 from children to children and from children to adults [40-45], with a lower prevalence of infection reported in younger children [46-48], with studies focusing primarily on household transmissions, while the role of schools remains unclear [47,49]. Young children infected with SARS-CoV-2 have viral loads in their respiratory tract similar to those of adolescents and adults [50,51]. School infection control measures played a role in decreasing outbreaks in some countries [52-54], but cluster outbreaks were reported among children in several provinces of South Africa when schools fully opened in 2022. Hence, further evaluation is needed to determine whether children (and the school setting) play a more substantive role in the community spread of SARS-CoV-2, especially in LMICs.

The overall aim of the COVID kids school study (CoKiDSS) is to assess SARS-CoV-2 prior infection, prior and current COVID-19 symptoms, seroprevalence, acute infection, transmission, immune responses, and symptoms of long COVID among a unique cohort of learners in grades 1-7, their parents/guardians, and teachers in the KwaZulu-Natal province of South Africa. The objectives of this study are shown in Table 2. Here, we describe the research study, its implementation processes, methodology, and expected results.

**Table 2:** COVID kids school study objectives.

Objective	Description
Primary objective	Objective 1: To assess the prevalence of self-reported confirmed SARS-CoV-2 prior infections, prior and current COVID-19 symptoms, and seroprevalence of SARS-CoV-2 antibodies in the overall triad of learners (grades 1-7), their parents/guardians, and teachers.
Secondary objectives	Objective 2: To determine the prevalence of long COVID-19 symptoms, general health and mental health in participants overall, and by SARS-CoV-2 antibody status. Objective 3: To determine the longitudinal changes in SARS-CoV-2 seroprevalence and symptoms in a subset of participants who are followed up.
Exploratory objectives	Objective 4: To conduct viral genome sequencing and describe transmission dynamics in a subgroup of 30 consenting SARS-CoV-2–positive individuals from the learner-parent-teacher triad and up to 10 of their close contacts. Objective 5: To determine the proportion of previously asymptomatic contacts who test SARS-CoV-2–positive above. Objective 6: To investigate B-cell and T-cell responses in the subgroup of 30 SARS-CoV-2–positive individuals enrolled and correlate these with symptoms.

## Methods

### Study Setting

This study will be conducted within the catchment areas of the South African Medical Research Council (SAMRC)’s Verulam Clinical Research Site (CRS) based in the Verulam suburb of the eThekwini district in KwaZulu-Natal. The KwaZulu-Natal province has the second largest population in South Africa, with an estimated 11.3 million people; 43% of the population are younger than 18 years [2]. The province is divided into 1 metropolitan municipality (eThekwini Metropolitan Municipality; Durban) and 11 district municipalities. Verulam is an urban area in the northern part of eThekwini Municipality in close proximity to the iLembe district municipality. Approximately 94% of the school-age children between 5 and 17 years are in school in this municipality [3]. The catchment areas of the Verulam CRS comprise urban, periurban, and rural localities. In South Africa, the COVID-19 pandemic evolved against a backdrop of longstanding tuberculosis and HIV epidemics. KwaZulu-Natal has the largest burden of HIV and tuberculosis infections [55], and of the 9 provinces in South Africa, KwaZulu-Natal had the third highest cumulative number of COVID-19 cases recorded as of June 29, 2022 [56].

### Study Design

**Figure 1:**
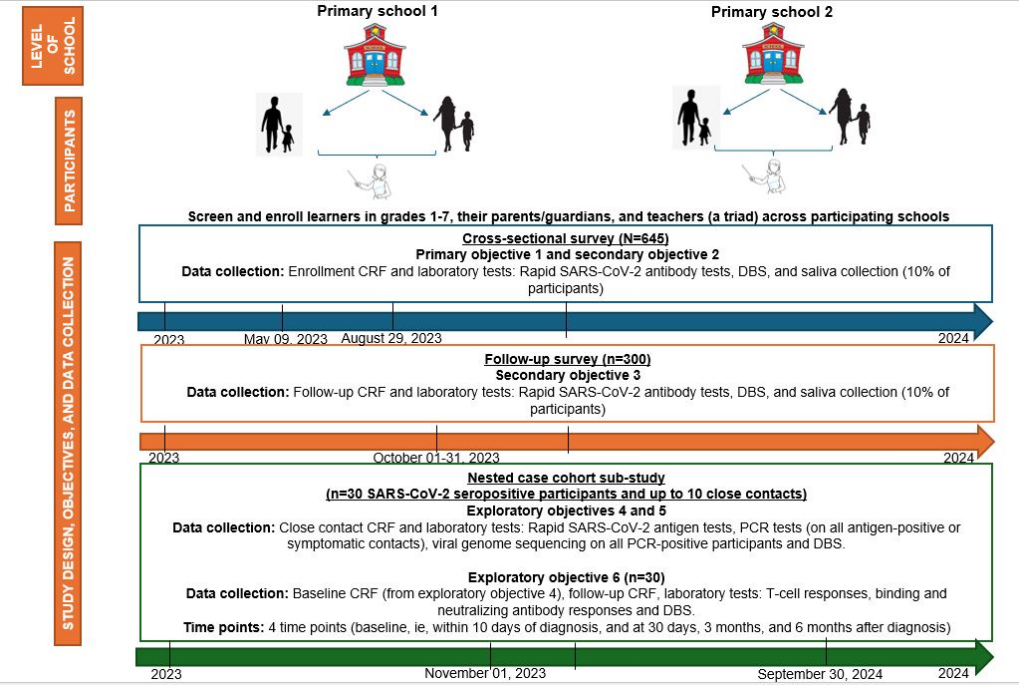
Study design, objectives, and data collection. CRF: case report form; DBS: dried blood spot; PCR: polymerase chain reaction.

This pilot study will implement a cross-sectional survey, follow up a subsample of survey participants, and conduct a nested case-cohort substudy in a preselect number of primary schools within the catchment area of the Verulam CRS in KwaZulu-Natal. Participating learners from primary schools (grades 1-7), their parents/guardians, and teachers will be invited to enroll in the CoKiDSS cross-sectional survey from May to August 2023 (primary objective 1; Figure 1). The cross-sectional survey will be conducted over 6 consecutive school weeks (window ±3 weeks; Figure 1). A subset of participants from the cross-sectional survey will be invited to participate in the follow-up survey (secondary objectives 2 and 3), with repeat SARS-CoV-2 antibody testing (3-4 months later from October 2023) until the desired sample is achieved (Figure 1). Saliva samples will be collected and stored from the first 10% (65/645 and 30/300, respectively) of the participants enrolled across the cross-sectional survey and in the follow-up survey for future testing. The nested case-cohort substudy (exploratory objectives 4-6) aims to enroll 30 cross-sectional survey participants with confirmed current SARS-CoV-2 infection—preferably all or at least 20 children (Figure 1) from November 2023 to September 2024. The follow-up survey will allow us to monitor the dynamics of SARS-CoV-2 seroprevalence. Long COVID will also be assessed in the cross-sectional and follow-up surveys as well as in the nested case-cohort substudy. Participants in the cross-sectional survey will have 1 study visit, while participants in the follow-up survey will have 2 study visits (Figure 1). During the follow-up survey, participants lost to follow-up will not be replaced.

For objectives 4-6, the first 30 participants in the cross-sectional surveys (learner/parent/teacher) who test SARS-CoV-2 polymerase chain reaction (PCR)–positive (30 children or at least 20 children) identified through the school or existing linkages with the National Institute of Communicable Diseases/National Health Laboratory Services will be invited to enroll into the exploratory substudy (nested case-cohort substudy; Figure 1). These participants are referred to as the primary positives. To assess transmission dynamics (objective 4) and proportion of asymptomatic cases (objective 5), all close contacts of the 30 primary positives (30 SARS-CoV-2 PCR-positive participants) up to a maximum of 10 contacts will be tested with a South African Health Products Regulatory Authority–approved SARS-CoV-2 point-of-care antigen test, and a swab will be sent to the National Health Laboratory Services for a SARS-CoV-2 PCR test. Only 1 swab will be collected from children who are primary positives or close contacts. For the primary positives and their PCR-positive contacts, remnant swabs will be sent for viral genome sequencing (objective 4; Figure 1). To assess B-cell and T-cell immune responses (objective 6), the primary positives (30 SARS-CoV-2 PCR-positive participants) will also provide venous blood at baseline (ie, within 10 days of a positive SARS-CoV-2 test), 30 days, 3 months, and 6 months post diagnosis (nested case-cohort substudy; Figure 1). Dried blood spot samples will be collected from all participants enrolled in the cross-sectional and follow-up surveys as well as the nested case-cohort substudy for future antibody testing.

### Study Populations

The inclusion and exclusion criteria for the school, school-age learners, their parents or guardians, and teachers are summarized in Table 3.

**Table 3:** Inclusion and exclusion criteria for the school, learners in grades 1-7, their parents/guardians, and teachers.

Participant and part of the study		Inclusion criteria	Exclusion criteria
**School**			
	Cross-sectional and follow-up surveys and nested case-cohort substudy	Primary schools within the catchment areas of the Verulam South African Medical Research Council clinical research site in eThekwini, KwaZulu-Natal, South Africa.	Special schools, learner referral units, and education colleges. Schools where other school-based COVID-19 studies are already being conducted. Small school size with <40 learners per grade in grades 1-7.
**Learners, parents/guardians, and teachers**			
	Cross-sectional survey	Learner must be attending school in person. A parent/guardian is only eligible if his/her child is eligible and participating in the study. A teacher is only eligible if the teacher teaches at a participating school. For learners <18 years of age, a parent/guardian is able to provide consent. For learners 8 to <18 years of age, the learner can provide assent. For learners 7 years of age, only parental/guardian consent is required. Willing for study staff to obtain routine SARS-CoV-2 results from National Institute of Communicable Diseases/National Health Laboratory Services.	Learners 8 to <18 years of age who are unable to provide appropriate informed assent. Parent excluded if learner already has 1 parent enrolled in the study. Learner does not anticipate completing the school year in the selected school. Learners with physical or intellectual challenges. Learners who, in the opinion of the principal investigators or designees, will be at risk if they participate in this study.
	Follow-up survey	Same criteria as for cross-sectional survey Learner must be attending school in person during the 2023 academic year. Willing to be followed up once and to give blood during follow-up.	Same criteria as for cross-sectional survey.

### Ethics Approval

This study has been approved by the SAMRC Human Research Ethics Committee (EC018-9/2022) and the South African Department of Basic Education (national and provincial) and the South African Department of Health (provincial). Written informed consent will be obtained from parents or guardians of participating learners and from all participating parents/guardians and teachers. Additionally, assent will be obtained from children aged 8 to <18 years. All participants will be reimbursed with a voucher valued at R 300 (US $16), in accordance with local ethics guidance to cover the cost of time, inconvenience, and expenses.

### Schools: Sampling and Sample Size

For objectives 1-6, a convenience sample of schools will be selected. In the first stage, a sampling frame of all public primary schools within the Verulam CRS catchment areas will be developed. Attempts will be made to obtain the head counts in these schools and number of classes. Only large schools (≥40 learners per grade in grades 1-7) able to contribute to the sample size will be shortlisted (short list 1) and their principals approached to ascertain interest. From short list 1, schools whose principals are agreeable to study participation will be identified (short list 2), and a sample of 2 primary schools will be selected from short list 2. We will expand to additional selected primary schools if needed, until the enrolment target is reached.

**Figure 2:**
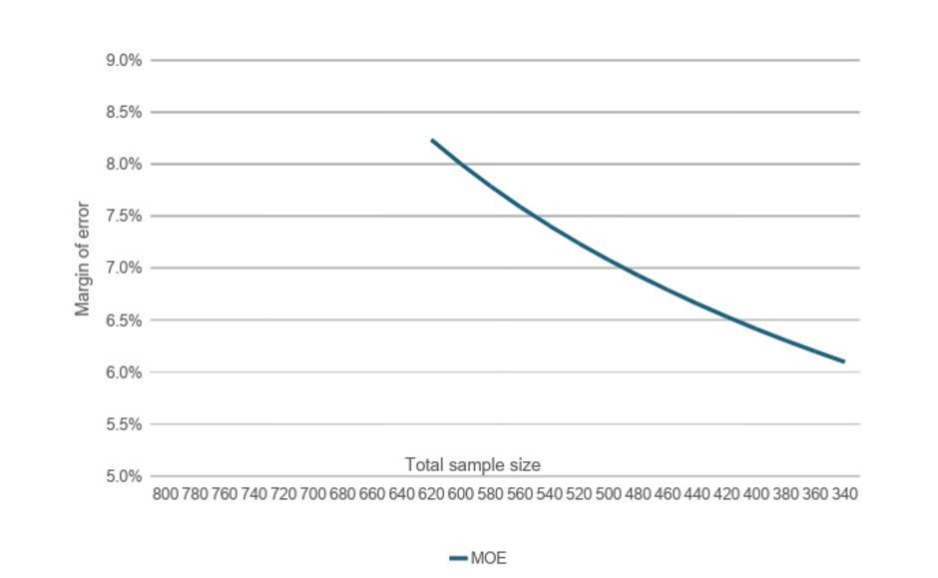
Margin of error for various sample sizes assuming a fixed prevalence of 40% and design effect of 2.5. MOE: margin of error.

Madhi et al [7] demonstrated a seroprevalence of 56.2% (95% CI 52.6%-59.7%) among children younger than 12 years in a seroepidemiological study conducted from October to December 2021 [7]. We opted to be more conservative due to the waning immunity anticipated in unvaccinated children or those who did not receive the booster dose almost 2 years after the pandemic. The sample size calculations for this study were based on estimating seroprevalence rates with a specific degree of precision. Specifically, the 
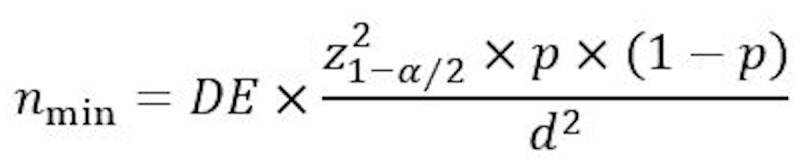
 was used, where DE refers to the design effect, p refers to the estimated prevalence, and d to the margin of error. A sample size of 640 will allow us to estimate seroprevalence rates in the combined group of 40% and greater with a margin of error of 6%, accounting for a design effect of 2.5 and assuming a 5% α. The survey is designed to be stratified by school, with class defining the cluster. We expect a minimum of 40 clusters in total across the 2 schools with an average cluster size of 16. In Figure 2, we have illustrated the margin of error for estimation under various sample sizes assuming all the other aforementioned parameters are fixed. As depicted in Figure 2, a reduction in the sample size below 600 will result in a greater margin of error (lower precision) larger than 7%.

A review of 14 studies indicated that long COVID symptoms varied from 4% to 66% among children and adolescents [57]. A cohort of 300 individuals will enable us to estimate the prevalence rates of long COVID at follow-up, 45% or greater with a 7% margin of error, accounting for a 20% loss to follow-up rate, and a maximum design effect of 1.2. Tables 4-5 summarize the sample size allocation for the respective surveys. The nested case-cohort substudy sample size is described in Table 6.

**Table 4:** Sample size allocation of primary school learners, parents, and teachers for the cross-sectional survey (N=640).^a^

Sample	Grade						
	1 (n=91)	2 (n=91)	3 (n=91)	4 (n=91)	5 (n=92)	6 (n=92)	7 (n=92)
Learners (n=451)	64	64	64	64	65	65	65
Parents (learner: parent, 3:1) (n=147)	21	21	21	21	21	21	21
Teachers (learner: teacher, 11:1) (n=42)	6	6	6	6	6	6	6

^a^The sample size might vary by ±10 people per group (learner, parent, teacher).

**Table 5:** Sample size allocation of primary school learners, parents, and teachers for the follow-up survey (n=300).^a^

Sample	Grade						
	1 (n=43)	2 (n=43)	3 (n=43)	4 (n=43)	5 (n=43)	6 (n=43)	7 (n=43)
Learners (% of cross-sectional survey ~50%) (n=210)	30	30	30	30	30	30	30
Parents (% of cross-sectional survey ~50%) (n=70)	10	10	10	10	10	10	10
Teachers (% of cross-sectional survey ~50%) (n=20)	3	3	3	3	3	3	2

^a^The sample size might vary by ±5 people per group (learner, parent, teacher).

**Table 6:** Sample size allocation for the nested case-cohort substudy.

Participants	Value, n
SARS-CoV-2–positive learners across grades 1-7	20-30
SARS-CoV-2–positive parents/teachers	0-10
Close contacts	10 per positive participant

### Study Procedures

#### Stakeholder Engagement

A strong community engagement program is underway to assist with buy-in and recruitment for the study among the Department of Basic Education, school principals, school governing boards, teachers, and parents/guardians of learners in grades 1-7. Schools that agree to participate will be asked to register and complete a short questionnaire.

#### Participant Recruitment and Follow-Up

Informed consent will be sought from teachers and from parents/guardians for their own/their children’s participation. Simplified informed consent forms were piloted to evaluate their understanding of the study and implications of the study. Assent will also be obtained from children aged 8 to <18 years, and participation will be contingent on obtaining both assent and parental consent. Only parental consent will be required for 7-year-old children. Flexible systems will be used to engage with parents, including weekend meetings and interviewer-administered or self-administered parental questionnaires. Multiple learners from one family can participate, but separate consent will be sought for each learner. Recruitment for the cross-sectional survey commenced on May 9, 2023, and was completed before the end of August 2023. The follow-up survey will follow up the first 210 learners, 70 parents, and 20 teachers who consent to follow-up at least 6 weeks after the first interview and after a school holiday. SARS-CoV-2–positive participants enrolled in the immunological substudy will be followed up for 6 months.

#### Data Collection

Questionnaire data will be gathered from school principals or a substitute member of the school senior leadership team, teachers, and parents/guardians of the primary school learners (in grades 1-7) (Table 7 and Multimedia Appendices 1-7).

Information will be collected on sociodemographic and basic health information; use of nonpharmacological measures; COVID-19 vaccination status of learner, parent/guardian, and teacher; household structure and COVID-19 history; symptoms of long COVID; household exposures; lifestyle; and preventive behavior related to the pandemic since January 2020 (see Multimedia Appendices 1-6). Parents/guardians will complete all the study case report forms on the children’s behalf (Multimedia Appendices 3 and 4).

Additionally, information will be collected on the learner’s lifestyle, mental health, and well-being. The Revised Children’ Anxiety and Depression Scale will be used to determine the internalizing symptoms of anxiety and depression among children and young adolescents enrolled in this study (see Multimedia Appendix 7) [58]. If the tool triggers negative memories in participants, these participants will be referred to a counsellor at the nearest health care facility. Samples will be collected from participants as outlined in Table 7 and described in Multimedia Appendix 8.

**Table 7:** Summary of data collection.

Participant	Cross-sectional and follow-up survey	Nested case-cohort substudy	
		Immunology	Transmission dynamics
School principal or senior school leadership	Questionnaire about number of learners, number of staff, and nonpharmaceutical interventions	N/A^a^	N/A
Learner/parent/teacher triad	Questionnaire at baseline and follow-up (if applicable) about contacts/nonpharmaceutical interventions/symptoms of long COVID. Blood draw to determine seroprevalence (rapid antibody test), dried blood spot for anti-S IgA/IgG, anti-N IgG, neutralizing antibody and antibody titers. Saliva samples will be collected in 10% of participants and stored if applicable.	N/A	N/A
Primary positives identification and follow-up within 10 days, 30 days, 3 months, and 6 months of a positive SARS-CoV-2–positive test	N/A	Questionnaire at baseline and follow-up (if applicable) about contacts/nonpharmaceutical interventions/symptoms of long COVID. Blood draw to determine seroprevalence (rapid antibody test), dried blood spot for anti-S IgA/IgG, anti-N IgG, neutralizing antibody and antibody titers. Saliva samples will be collected in 10% of participants and stored if applicable.	Telephonic questionnaire to identify contacts. Initial swab sent for viral genome sequencing.
Contacts of the primary positives	May or may not be included in the cross-sectional and follow-up surveys.	N/A	Questionnaire + rapid antigen test + polymerase chain reaction + dried blood spot

^a^N/A: not applicable.

### Data Management

Data will be collected in research electronic data capture, a secure web-based application hosted by the SAMRC. More information on this platform can be found elsewhere [59,60]. Research electronic data capture access will be restricted to CoKiDSS personnel. A lookup function will be created in research electronic data capture to link/track participants enrolled in the cross-sectional survey, follow-up survey, and nested case-cohort substudy. This will be useful if the close contact is already a participant in the study.

### Biospecimen Management

Samples will be stored at the HIV and other Infectious Diseases Research Unit, SAMRC, biorepository. The Laboratory Data Management System program developed by Frontier Science is used by the HIV and other Infectious Diseases Research Unit biorepository and is a storage module that conforms to US Food and Drug Administration, 21 Code of Federal Regulations part 58 and 11 [61]. The program is utilized to manage specimen tracking, inventory storage, and specimen shipment.

### Data Analysis

We will perform descriptive analysis of participant sociodemographic, lifestyle, and behavior information. Total seroprevalence and cumulative incidence (ie, total number of reverse transcription-PCR–confirmed infections in official statistics per population) will be calculated and compared as well as age-specific and time-specific estimates. The total numbers of learners in the respective grades per school will be used for poststratification so that the estimates are representative of the demographics in KwaZulu-Natal, which will be incorporated via weights. The prevalence prior and current COVID-19 symptoms and seroprevalence will be presented with 95% CIs, where standard errors will be computed via jackknife methods. Within the cohort, changes in seroprevalence, symptoms, mental health, and physical health will be assessed using generalized linear mixed models, considering the within-individual correlation of responses as well as the hierarchical structure of the data. The clustering of seropositivity within classes and school will be quantified using the intracluster correlation. To analyze the exploratory objectives, the following statistical methods will be employed: (1) means with standard deviations or medians with interquartile ranges (as appropriate depending on the distribution) will be presented for continuous data, (2) the graphical presentation of continuous outcomes will be undertaken using box plots, (3) bar charts will be used to represent categorical data across groups of interest, and (4) the correlation between variables will be addressed using 2-way scatterplots with the presentation of Spearman correlation coefficient.

### Patient and Public Involvement

Several school principals were consulted during the development of the protocol to ensure the feasibility of the planned study procedures. Early feedback was collected from learners and parents invited to participate to adapt the communication strategies and channels. Further feedback will also be collected from enrolled learners and school principals during the cross-sectional survey to adapt the follow-up survey. Results of individual tests will be communicated to the participants, and overall study results disseminated to participating schools. Findings will be disseminated to the South African Department of Basic Education and Department of Health.

## Results

Recruitment for the cross-sectional survey occurred between May and August 2023, and a total of 645 participants were enrolled. Three hundred participants were followed up in the follow-up survey implemented in October 2023. Screening of the participants into the nested case-cohort substudy is planned between November 2023 and September 2024. Data cleanup and analysis for the cross-sectional survey is complete. Data cleanup and analysis for the follow-up survey and nested case substudy will be completed in the third quarter of 2024. The dissemination and publication of study findings is anticipated for the fourth quarter of 2024. The abovementioned activities and timelines are outlined in Multimedia Appendix 9.

## Discussion

### Anticipated Main Findings of This Study

We anticipate that this study will find high SARS-CoV-2 seroprevalence, especially among teachers and parents, despite low reported SARS-CoV-2 infections and low prevalence of long COVID-19 and that transmission dynamics will favor parent-child transmission.

### Contextualization of the Anticipated Results

The direct impact of COVID-19 on child and adolescent mortality is limited, with these age groups accounting for a meagre 0.4% (over 17,400) of COVID-19 deaths worldwide as of March 2023 [1]. However, according to UNICEF (United Nations Children’s Fund), children and adolescents bear the brunt of the indirect effects of the COVID-19 pandemic, including more households plummeting into multidimensional poverty, exacerbating the hardships of children living in the poorest countries, augmenting the learning crisis, threatening child survival and death, increasing child malnutrition, and deprivation and disruptions in health services [62]. Hence, the harms associated with school closures were profound. CoKiDSS is centered around the school rather than community settings, as schools play a critical role in a child’s learning and development, and in many countries, schools also provide access to immunizations, health care, and nutritional services.

In LMICs, among children aged 5-13 years, there is a paucity of data on SARS-CoV-2 infections, including seroprevalence and transmission dynamics. In South Africa, 3 cardinal community studies have been published to date, reporting SARS-CoV-2 seroprevalence among adults and children [5-7]. However, the last serosurvey was conducted in December 2021 during the Omicron wave, and children constituted a small proportion of the study population [7].

CoKiDSS will provide data on the impact of COVID-19 on school-attending children ~36 months after the pandemic was declared and 21-24 months after the resumption of normal school attendance. In particular, data will be provided on the prevalence of confirmed prior SARS-CoV-2 infections, prior and current COVID-19 symptoms, seroprevalence of SARS-CoV-2 antibodies, prevalence of long COVID symptoms, and general and mental health in a triad of learners, their parents/guardians, and teachers in the current COVID-19 endemic. The longitudinal design will allow a description of the temporal trends of immunity to SARS-CoV-2. Presently, in South Africa, children aged 5-11 years are only eligible to receive SARS-CoV-2 vaccination if they are at risk of developing severe COVID-19. The CoKiDSS data will provide a better understanding on the natural protection within this population and could potentially guide local vaccination strategies.

### Strengths and Limitations

This study is the first study of SARS-CoV-2 infections in a school setting within a high HIV/tuberculosis prevalence LMIC. Thus, it will provide information on the impact of COVID-19 on schools, learners, teachers, and parents in these settings, and could guide future responses to pandemics within these settings. However, this study is not without challenges and limitations. Given the limited geographic area and number of schools, the findings may not be representative of all South African schools. In order to recruit a triad of learners in grades 1-7, their parents, and teachers, several rounds of engagement may be needed, resulting in oversampling or undersampling at schools. This study will assess SARS-CoV-2 seroprevalence by using a qualitative SARS-CoV-2 point-of-care antibody test. It is possible that the qualitative antibody assessment is below the threshold of conferring immunity. We would, however, prefer to use a minimally invasive test in this vulnerable population. Notwithstanding these, this study will contribute to the limited body of knowledge on the effect of the COVID-19 pandemic on school-attending children 21-24 months after resumption of normal school attendance.

### Dissemination Plan

The study objectives and outcomes will be disseminated to participants, community, and other stakeholders, including the South African Department of Health and South African Department of Basic Education (district, provincial, and national levels), regulatory bodies, research organizations, community members, and participants. Additionally, the information linked to this study will be shared by the community staff with the participating school principals and members of the school governing board as well as the community advisory board/community working group members. The results of this study will be disseminated via virtual methods and face-to-face meetings. Communication methods will include the use of SMS text messaging to mobiles, monthly telephone calls with community advisory board members, hand delivery of relevant written material, email, Microsoft Teams, and social media platforms (WhatsApp). These methods will contribute to ongoing community acceptance of the study and trust in the research team and the program.

## Appendix

Multimedia Appendix 1 Principal enrollment and follow-up case report form.

Multimedia Appendix 2 Teacher enrollment and follow-up case report form.

Multimedia Appendix 3 Parent of learner enrollment and follow-up e-case report form.

Multimedia Appendix 4 Parent of learner enrollment and follow-up case report form.

Multimedia Appendix 5 Close contact case report form.

Multimedia Appendix 6 Nested case-cohort substudy case report form.

Multimedia Appendix 7 Learner mental health case report form.

Multimedia Appendix 8 Detailed data collection.

Multimedia Appendix 9 Study timeline.

## Abbreviations

CoKiDSS: COVID kids school study
CRS: clinical research site
LMIC: low- and middle-income country
PCR: polymerase chain reaction
SAMRC: South African Medical Research Council
UNICEF: United Nations Children’s Fund

## Data Availability

Data availability will need to be requested from the corresponding author. The request will be considered a concept note, and the reason for data access will be required. Data will only be shared after the minimum papers have been written by the authors. Data will be made publicly available on the South African Medical Research Council website after all the main papers have been published.

## References

[1] Child mortality and COVID-19. UNICEF.

[2] (2019). Mid-year population estimates. Statistics South Africa, Statistical release P0302.

[3] Monthly COVID-19 in children. National Institute of Communicable Diseases.

[4] Sutton D, Fuchs K, D’Alton M, Goffman D (2020). Universal screening for SARS-CoV-2 in women admitted for delivery. N Engl J Med.

[5] Cohen C, Kleynhans J, von Gottberg Anne, McMorrow M, Wolter N, Bhiman J, Moyes Jocelyn, du Plessis Mignon, Carrim Maimuna, Buys Amelia, Martinson Neil A, Kahn Kathleen, Tollman Stephen, Lebina Limakatso, Wafawanaka Floidy, du Toit Jacques D, Gómez-Olivé Francesc Xavier, Dawood Fatimah S, Mkhencele Thulisa, Sun Kaiyuan, Viboud Cécile, Tempia Stefano, PHIRST-C Group (2022). SARS-CoV-2 incidence, transmission, and reinfection in a rural and an urban setting: results of the PHIRST-C cohort study, South Africa, 2020-21. Lancet Infect Dis.

[6] Moyo Sizulu, Simbayi Leickness C, Zuma K, Zungu Nompumelelo, Marinda Edmore, Jooste Sean, Ramlagan Shandir, Fortuin Mirriam, Singh Beverley, Mabaso Musawenkosi, Reddy Tarylee, Parker Whadi-Ah, Naidoo Inbarani, Manda Samuel, Goga Ameena, Ngandu Nobubelo, Cawood Cherie, Moore Penny L, Puren Adrian (2023). Seroprevalence survey of anti-SARS-CoV-2 antibody and associated factors in South Africa: Findings of the 2020-2021 population-based household survey. PLOS Glob Public Health.

[7] Madhi S, Kwatra G, Myers J, Jassat W, Dhar N, Mukendi C, Nana Amit J, Blumberg Lucille, Welch Richard, Ngorima-Mabhena Nicoletta, Mutevedzi Portia C (2022). Population immunity and COVID-19 severity with Omicron variant in South Africa. N Engl J Med.

[8] Novel coronavirus (2019-nCoV). WHO.

[9] Cox D (2023). What do we know about COVID-19 and children?. British Medical Journal.

[10] Dong Y, Mo X, Hu Y, Qi X, Jiang F, Jiang Z, Tong S (2020). Epidemiology of COVID-19 among children in China. Pediatrics.

[11] Smith C, Odd D, Harwood R, Ward J, Linney M, Clark M, Hargreaves D, Ladhani SN, Draper E, Davis PJ, Kenny SE, Whittaker E, Luyt K, Viner R, Fraser LK (2021). Deaths in children and young people in England after SARS-CoV-2 infection during the first pandemic year. Nat Med.

[12] Ward J, Harwood R, Smith C, Kenny S, Clark M, Davis P (2022). Risk factors for PICU admission and death among children and young people hospitalized with COVID-19 and PIMS-TS in England during the first pandemic year. Nature Medicine.

[13] Buonsenso D, Munblit D, De RC, Sinatti D, Ricchiuto A, Carfi A, Valentini P (2021). Preliminary evidence on long COVID in children. Acta Paediatics.

[14] Loske J, Röhmel J, Lukassen S, Stricker S, Magalhães V G, Liebig J, Chua R L, Thürmann L, Messingschlager M, Seegebarth A, Timmermann B, Klages S, Ralser M, Sawitzki B, Sander L E, Corman V M, Conrad C, Laudi S, Binder M, Trump S, Eils R, Mall M A, Lehmann I (2022). Pre-activated antiviral innate immunity in the upper airways controls early SARS-CoV-2 infection in children. Nat Biotechnol.

[15] Pierce C, Sy S, Galen B, Goldstein D, Orner E, Keller M, Herold Kevan C, Herold Betsy C (2021). Natural mucosal barriers and COVID-19 in children. JCI Insight.

[16] Winkley K, Banerjee D, Bradley T, Koseva B, Cheung W, Selvarangan R, Pastinen Tomi, Grundberg Elin (2021). Immune cell residency in the nasal mucosa may partially explain respiratory disease severity across the age range. Sci Rep.

[17] Zhu Y, Chew K, Karawita A, Yamamoto A, Labzin L, Yarlagadds T Pediatric nasal epithelial cells are less permissive to SARS-CoV-2 replication compared to adult cells. BioRxiv.

[18] Carsetti R, Zaffina S, Piano Mortari E, Terreri S, Corrente F, Capponi C, Palomba P, Mirabella M, Cascioli S, Palange P, Cuccaro I, Milito C, Zumla A, Maeurer M, Camisa V, Vinci Mr, Santoro A, Cimini E, Marchioni L, Nicastri E, Palmieri F, Agrati C, Ippolito G, Porzio O, Concato C, Onetti Muda A, Raponi M, Quintarelli C, Quinti I, Locatelli F (2020). Different innate and adaptive immune responses to SARS-CoV-2 infection of asymptomatic, mild, and severe cases. Front. Immunol.

[19] Bordallo Bruno, Bellas Mozart, Cortez Arthur Fernandes, Vieira Matheus, Pinheiro Marcelo (2020). Severe COVID-19: what have we learned with the immunopathogenesis?. Adv Rheumatol.

[20] Bartsch Y, Wang C, Zohar T, Fischinger S, Atyeo C, Burke J, Kang Jaewon, Edlow Andrea G, Fasano Alessio, Baden Lindsey R, Nilles Eric J, Woolley Ann E, Karlson Elizabeth W, Hopke Alex R, Irimia Daniel, Fischer Eric S, Ryan Edward T, Charles Richelle C, Julg Boris D, Lauffenburger Douglas A, Yonker Lael M, Alter Galit (2021). Humoral signatures of protective and pathological SARS-CoV-2 infection in children. Nat Med.

[21] Neeland M, Bannister S, Clifford V, Dohle K, Mulholland K, Sutton P, Curtis Nigel, Steer Andrew C, Burgner David P, Crawford Nigel W, Tosif Shidan, Saffery Richard (2021). Innate cell profiles during the acute and convalescent phase of SARS-CoV-2 infection in children. Nat Commun.

[22] Bordoni V, Sacchi A, Cimini E, Notari S, Grassi G, Tartaglia Eleonora, Casetti Rita, Giancola Maria Letizia, Bevilacqua Nazario, Maeurer Markus, Zumla Alimuddin, Locatelli Franco, De Benedetti Fabrizio, Palmieri Fabrizio, Marchioni Luisa, Capobianchi Maria R, D'Offizi Gianpiero, Petrosillo Nicola, Antinori Andrea, Nicastri Emanuele, Ippolito Giuseppe, Agrati Chiara (2020). An inflammatory profile correlates with decreased frequency of cytotoxic cells in coronavirus disease 2019. Clin Infect Dis.

[23] Li H, Chen K, Liu M, Xu Hua, Xu Q (2020). The profile of peripheral blood lymphocyte subsets and serum cytokines in children with 2019 novel coronavirus pneumonia. J Infect.

[24] Pierce C, Preston-Hurlburt P, Dai Yile, Aschner Clare Burn, Cheshenko Natalia, Galen Benjamin, Garforth Scott J, Herrera Natalia G, Jangra Rohit K, Morano Nicholas C, Orner Erika, Sy Sharlene, Chandran Kartik, Dziura James, Almo Steven C, Ring Aaron, Keller Marla J, Herold Kevan C, Herold Betsy C (2020). Immune responses to SARS-CoV-2 infection in hospitalized pediatric and adult patients. Sci Transl Med.

[25] Yoshida M, Worlock K, Huang N, Lindeboom R, Butler C, Kumasaka N, Dominguez Conde Cecilia, Mamanova Lira, Bolt Liam, Richardson Laura, Polanski Krzysztof, Madissoon Elo, Barnes Josephine L, Allen-Hyttinen Jessica, Kilich Eliz, Jones Brendan C, de Wilton Angus, Wilbrey-Clark Anna, Sungnak Waradon, Pett J Patrick, Weller Juliane, Prigmore Elena, Yung Henry, Mehta Puja, Saleh Aarash, Saigal Anita, Chu Vivian, Cohen Jonathan M, Cane Clare, Iordanidou Aikaterini, Shibuya Soichi, Reuschl Ann-Kathrin, Herczeg Iván T, Argento A Christine, Wunderink Richard G, Smith Sean B, Poor Taylor A, Gao Catherine A, Dematte Jane E, Reynolds Gary, Haniffa Muzlifah, Bowyer Georgina S, Coates Matthew, Clatworthy Menna R, Calero-Nieto Fernando J, Göttgens Berthold, O'Callaghan Christopher, Sebire Neil J, Jolly Clare, De Coppi Paolo, Smith Claire M, Misharin Alexander V, Janes Sam M, Teichmann Sarah A, Nikolić Marko Z, Meyer Kerstin B, NU SCRIPT Study Investigators (2022). Local and systemic responses to SARS-CoV-2 infection in children and adults. Nature.

[26] Fazolo T, Lima K, Fontoura J, de Souza Priscila Oliveira, Hilario G, Zorzetto Renata, Júnior Luiz Rodrigues, Pscheidt Veridiane Maria, de Castilhos Ferreira Neto Jayme, Haubert Alisson F, Gambin Izza, Oliveira Aline C, Mello Raissa S, de Bastos Balbe E Gutierres Matheus, Gassen Rodrigo Benedetti, Coimbra Lais Durço, Borin Alexandre, Marques Rafael Elias, Sartor Ivaine Tais Sauthier, Zavaglia Gabriela Oliveira, Fernandes Ingrid Rodrigues, Nakaya Helder I, Varela Fernanda Hammes, Polese-Bonatto Márcia, Borges Thiago J, Callegari-Jacques Sidia Maria, da Costa Marcela Santos Correa, de Araujo Schwartz Jaqueline, Scotta Marcelo Comerlato, Stein Renato T, Bonorino Cristina (2021). Pediatric COVID-19 patients in South Brazil show abundant viral mRNA and strong specific anti-viral responses. Nat Commun.

[27] Cohen CA, Li APY, Hachim A, Hui DSC, Kwan MYW, Tsang OTY, Chiu Susan S, Chan Wai Hung, Yau Yat Sun, Kavian Niloufar, Ma Fionn N L, Lau Eric H Y, Cheng Samuel M S, Poon Leo L M, Peiris Malik, Valkenburg Sophie A (2021). SARS-CoV-2 specific T cell responses are lower in children and increase with age and time after infection. Nat Commun.

[28] Goenka A, Halliday A, Gregorova M, Milodowski E, Thomas A, Williamson M, Baum Holly, Oliver Elizabeth, Long Anna E, Knezevic Lea, Williams Alistair J K, Lampasona Vito, Piemonti Lorenzo, Gupta Kapil, Di Bartolo Natalie, Berger Imre, Toye Ashley M, Vipond Barry, Muir Peter, Bernatoniene Jolanta, Bailey Mick, Gillespie Kathleen M, Davidson Andrew D, Wooldridge Linda, Rivino Laura, Finn Adam (2021). Young infants exhibit robust functional antibody responses and restrained IFN-γ production to SARS-CoV-2. Cell Rep Med.

[29] Ji S, Zhang M, Zhang Y, Xia K, Chen Y, Chu Q, Wei Yong-Chang, Zhou Fu-Ling, Bu Bi-Tao, Tu Hong-Lei, Cao Ya-Yun, Hu Li-Ya (2021). Characteristics of immune and inflammatory responses among different age groups of pediatric patients with COVID-19 in China. World J Pediatr.

[30] Cervia C, Nilsson J, Zurbuchen Yves, Valaperti Alan, Schreiner Jens, Wolfensberger Aline, Raeber Miro E, Adamo Sarah, Weigang Sebastian, Emmenegger Marc, Hasler Sara, Bosshard Philipp P, De Cecco Elena, Bächli Esther, Rudiger Alain, Stüssi-Helbling Melina, Huber Lars C, Zinkernagel Annelies S, Schaer Dominik J, Aguzzi Adriano, Kochs Georg, Held Ulrike, Probst-Müller Elsbeth, Rampini Silvana K, Boyman Onur (2021). Systemic and mucosal antibody responses specific to SARS-CoV-2 during mild versus severe COVID-19. J Allergy Clin Immunol.

[31] Renk H, Dulovic A, Seidel Alina, Becker Matthias, Fabricius Dorit, Zernickel Maria, Junker Daniel, Groß Rüdiger, Müller Janis, Hilger Alexander, Bode Sebastian F N, Fritsch Linus, Frieh Pauline, Haddad Anneke, Görne Tessa, Remppis Jonathan, Ganzemueller Tina, Dietz Andrea, Huzly Daniela, Hengel Hartmut, Kaier Klaus, Weber Susanne, Jacobsen Eva-Maria, Kaiser Philipp D, Traenkle Bjoern, Rothbauer Ulrich, Stich Maximilian, Tönshoff Burkhard, Hoffmann Georg F, Müller Barbara, Ludwig Carolin, Jahrsdörfer Bernd, Schrezenmeier Hubert, Peter Andreas, Hörber Sebastian, Iftner Thomas, Münch Jan, Stamminger Thomas, Groß Hans-Jürgen, Wolkewitz Martin, Engel Corinna, Liu Weimin, Rizzi Marta, Hahn Beatrice H, Henneke Philipp, Franz Axel R, Debatin Klaus-Michael, Schneiderhan-Marra Nicole, Janda Ales, Elling Roland (2022). Robust and durable serological response following pediatric SARS-CoV-2 infection. Nat Commun.

[32] Hachim A, Gu H, Kavian O, Kwan M, Wai-hung C, Yau Y The SARS-CoV-2 antibody landscape is lower in magnitude for structural proteins, diversified for accessory proteins and stable long-term in children. MedRxiv.

[33] Weisberg S, Connors T, Zhu Y, Baldwin M, Lin W, Wontakal S, Szabo Peter A, Wells Steven B, Dogra Pranay, Gray Joshua, Idzikowski Emma, Stelitano Debora, Bovier Francesca T, Davis-Porada Julia, Matsumoto Rei, Poon Maya Meimei Li, Chait Michael, Mathieu Cyrille, Horvat Branka, Decimo Didier, Hudson Krystalyn E, Zotti Flavia Dei, Bitan Zachary C, La Carpia Francesca, Ferrara Stephen A, Mace Emily, Milner Joshua, Moscona Anne, Hod Eldad, Porotto Matteo, Farber Donna L (2021). Distinct antibody responses to SARS-CoV-2 in children and adults across the COVID-19 clinical spectrum. Nat Immunol.

[34] Dowell AC, Butler MS, Jinks E, Tut G, Lancaster T, Sylla P, Begum Jusnara, Bruton Rachel, Pearce Hayden, Verma Kriti, Logan Nicola, Tyson Grace, Spalkova Eliska, Margielewska-Davies Sandra, Taylor Graham S, Syrimi Eleni, Baawuah Frances, Beckmann Joanne, Okike Ifeanyichukwu O, Ahmad Shazaad, Garstang Joanna, Brent Andrew J, Brent Bernadette, Ireland Georgina, Aiano Felicity, Amin-Chowdhury Zahin, Jones Samuel, Borrow Ray, Linley Ezra, Wright John, Azad Rafaq, Waiblinger Dagmar, Davis Chris, Thomson Emma C, Palmarini Massimo, Willett Brian J, Barclay Wendy S, Poh John, Amirthalingam Gayatri, Brown Kevin E, Ramsay Mary E, Zuo Jianmin, Moss Paul, Ladhani Shamez (2022). Children develop robust and sustained cross-reactive spike-specific immune responses to SARS-CoV-2 infection. Nat Immunol.

[35] Renk H, Dulovic A, Seidel A, Becker M, Fabricius D, Zernickel M, Junker D, Groß R, Müller J, Hilger A, Bode SFN, Fritsch L, Frieh P, Haddad A, Görne T, Remppis J, Ganzemueller T, Dietz A, Huzly D, Hengel H, Kaier K, Weber S, Jacobsen E, Kaiser PD, Traenkle B, Rothbauer U, Stich M, Tönshoff B, Hoffmann GF, Müller B, Ludwig C, Jahrsdörfer B, Schrezenmeier H, Peter A, Hörber S, Iftner T, Münch J, Stamminger T, Groß H, Wolkewitz M, Engel C, Liu W, Rizzi M, Hahn BH, Henneke P, Franz AR, Debatin K, Schneiderhan-Marra N, Janda A, Elling R (2022). Robust and durable serological response following pediatric SARS-CoV-2 infection. Nat Commun.

[36] Messiah S, DeSantis S, Leon-Novelo L, Talebi Y, Brito F, Kohl H, Valerio-Shewmaker Melissa A, Ross Jessica A, Swartz Michael D, Yaseen Ashraf, Kelder Steven H, Zhang Shiming, Omega-Njemnobi Onyinye S, Gonzalez Michael O, Wu Leqing, Boerwinkle Eric, Lakey David L, Shuford Jennifer A, Pont Stephen J (2022). Durability of SARS-CoV-2 antibodies from natural infection in children and adolescents. Pediatrics.

[37] Ulyte A, Radtke T, Abela I, Haile S, Braun J, Jung R, Berger Christoph, Trkola Alexandra, Fehr Jan, Puhan Milo A, Kriemler Susi (2020). Seroprevalence and immunity of SARS-CoV-2 infection in children and adolescents in schools in Switzerland: design for a longitudinal, school-based prospective cohort study. Int J Public Health.

[38] Litvinova M, Liu QH, Kulikov ES, Ajelli M (2019). Reactive school closure weakens the network of social interactions and reduces the spread of influenza. Proc Natl Acad Sci U S A.

[39] Cauchemez S, Ferguson N, Wachtel C, Tegnell A, Saour G, Duncan B, Nicoll A (2009). Closure of schools during an influenza pandemic. Lancet Infect Dis.

[40] Bai Y, Yao L, Wei T, Tian F, Jin D, Chen L, Wang M (2020). Presumed asymptomatic carrier transmission of COVID-19. JAMA.

[41] Wei WE, Li Z, Chiew CJ, Yong SE, Toh MP, Lee VJ (2020). Presymptomatic transmission of SARS-CoV-2 — Singapore, January 23-March 16, 2020. MMWR Morb Mortal Wkly Rep.

[42] Kam K, Yung C, Cui L, Tzer Pin Lin Raymond, Mak T, Maiwald M, Li Jiahui, Chong Chia Yin, Nadua Karen, Tan Natalie Woon Hui, Thoon Koh Cheng (2020). A well infant with coronavirus disease 2019 with high viral load. Clin Infect Dis.

[43] Huff H, Singh A (2020). Asymptomatic transmission during the coronavirus disease 2019 pandemic and implications for public health strategies. Clin Infect Dis.

[44] Jiehao Cai, Jin Xu, Daojiong Lin, Zhi U, Lei X, Zhenghai Qu, Yuehua Zhang, Hua Zhang, Ran Jia, Pengcheng Liu, Xiangshi Wang, Yanling Ge, Aimei Xia, He Tian, Hailing Chang, Chuning Wang, Jingjing Li, Jianshe Wang, Mei Zeng (2020). A case series of children with 2019 novel coronavirus infection: clinical and epidemiological features. Clin Infect Dis.

[45] Teherani M, Kao C, Camacho-Gonzalez A, Banskota S, Shane A, Linam W, Jaggi P (2020). Burden of illness in households with severe acute respiratory syndrome coronavirus 2-infected children. J Pediatric Infect Dis Soc.

[46] Viner R, Russell S, Croker H, Packer J, Ward J, Stansfield C, Mytton Oliver, Bonell Chris, Booy Robert (2020). School closure and management practices during coronavirus outbreaks including COVID-19: a rapid systematic review. Lancet Child Adolesc Health.

[47] Stringhini S, Wisniak A, Piumatti G, Azman Andrew S, Lauer S, Baysson H, De Ridder David, Petrovic Dusan, Schrempft Stephanie, Marcus Kailing, Yerly Sabine, Arm Vernez Isabelle, Keiser Olivia, Hurst Samia, Posfay-Barbe Klara M, Trono Didier, Pittet Didier, Gétaz Laurent, Chappuis François, Eckerle Isabella, Vuilleumier Nicolas, Meyer Benjamin, Flahault Antoine, Kaiser Laurent, Guessous Idris (2020). Seroprevalence of anti-SARS-CoV-2 IgG antibodies in Geneva, Switzerland (SEROCoV-POP): a population-based study. Lancet.

[48] Gudbjartsson D, Helgason A, Jonsson H, Magnusson O, Melsted P, Norddahl G, Saemundsdottir Jona, Sigurdsson Asgeir, Sulem Patrick, Agustsdottir Arna B, Eiriksdottir Berglind, Fridriksdottir Run, Gardarsdottir Elisabet E, Georgsson Gudmundur, Gretarsdottir Olafia S, Gudmundsson Kjartan R, Gunnarsdottir Thora R, Gylfason Arnaldur, Holm Hilma, Jensson Brynjar O, Jonasdottir Aslaug, Jonsson Frosti, Josefsdottir Kamilla S, Kristjansson Thordur, Magnusdottir Droplaug N, le Roux Louise, Sigmundsdottir Gudrun, Sveinbjornsson Gardar, Sveinsdottir Kristin E, Sveinsdottir Maney, Thorarensen Emil A, Thorbjornsson Bjarni, Löve Arthur, Masson Gisli, Jonsdottir Ingileif, Möller Alma D, Gudnason Thorolfur, Kristinsson Karl G, Thorsteinsdottir Unnur, Stefansson Kari (2020). Spread of SARS-CoV-2 in the Icelandic Population. N Engl J Med.

[49] Pollán Marina, Pérez-Gómez Beatriz, Pastor-Barriuso R, Oteo J, Hernán Miguel A, Pérez-Olmeda Mayte, Sanmartín Jose L, Fernández-García Aurora, Cruz Israel, Fernández de Larrea Nerea, Molina Marta, Rodríguez-Cabrera Francisco, Martín Mariano, Merino-Amador Paloma, León Paniagua Jose, Muñoz-Montalvo Juan F, Blanco Faustino, Yotti Raquel, ENE-COVID Study Group (2020). Prevalence of SARS-CoV-2 in Spain (ENE-COVID): a nationwide, population-based seroepidemiological study. Lancet.

[50] Heald-Sargent T, Muller W, Zheng X, Rippe J, Patel A, Kociolek L (2020). Age-related differences in nasopharyngeal severe acute respiratory syndrome coronavirus 2 (SARS-CoV-2) levels in patients with mild to moderate coronavirus disease 2019 (COVID-19). JAMA Pediatr.

[51] Jones T, Biele Guido, Mühlemann Barbara, Veith Talitha, Schneider Julia, Beheim-Schwarzbach Jörn, Bleicker Tobias, Tesch Julia, Schmidt Marie Luisa, Sander Leif Erik, Kurth Florian, Menzel Peter, Schwarzer Rolf, Zuchowski Marta, Hofmann Jörg, Krumbholz Andi, Stein Angela, Edelmann Anke, Corman Victor Max, Drosten Christian (2021). Estimating infectiousness throughout SARS-CoV-2 infection course. Science.

[52] Larosa E, Djuric O, Cassinadri M, Cilloni S, Bisaccia E, Vicentini M, Venturelli Francesco, Giorgi Rossi Paolo, Pezzotti Patrizio, Bedeschi Emanuela, Reggio Emilia Covid-19 Working Group (2020). Secondary transmission of COVID-19 in preschool and school settings in northern Italy after their reopening in September 2020: a population-based study. Euro Surveill.

[53] Zimmerman K, Akinboyo I, Brookhart M, Boutzoukas Angelique E, McGann Kathleen A, Smith Michael J, Maradiaga Panayotti Gabriela, Armstrong Sarah C, Bristow Helen, Parker Donna, Zadrozny Sabrina, Weber David J, Benjamin Daniel K, Science Collaborative Abc (2021). Incidence and secondary transmission of SARS-CoV-2 infections in schools. Pediatrics.

[54] Brandal L, Ofitserova T, Meijerink H, Rykkvin R, Lund H, Hungnes O, Greve-Isdahl Margrethe, Bragstad Karoline, Nygård Karin, Winje Brita A (2021). Minimal transmission of SARS-CoV-2 from paediatric COVID-19 cases in primary schools, Norway, August to November 2020. Euro Surveill.

[55] (2020). Strategic plan 2020/2021-2024/2025. KwaZulu-Natal DOH.

[56] COVID-19 online resource and portal. National Department of Health, Republic of South Africa.

[57] Zimmermann Petra, Pittet Laure F, Curtis Nigel (2021). How common is long COVID in children and adolescents?. Pediatr Infect Dis J.

[58] The Revised Children's Anxiety and Depression Scale (RCADS). UCLA.

[59] Laboratory data management system. Frontier Science Foundation.

[60] Harris P, Taylor R, Thielke R, Payne J, Gonzalez N, Conde J (2009). Research electronic data capture (REDCap)--a metadata-driven methodology and workflow process for providing translational research informatics support. J Biomed Inform.

[61] Harris P, Taylor R, Minor B, Elliott V, Fernandez M, O'Neal L, McLeod L, Delacqua G, Delacqua F, Kirby J, Duda Sn (2019). The REDCap consortium: Building an international community of software platform partners. Journal of Biomedical Informatics.

[62] COVID-19 and children. UNICEF.

