# Supplementary material for: SARS-CoV-2 Infections in a Triad of Primary School Learners (Grades 1-7), Their Parents, and Teachers in KwaZulu-Natal, South Africa: Protocol for a Cross-Sectional and Nested Case-Cohort Study
Source: JMIR Res Protoc. 2024 Dec 19;13:e52713. doi: 10.2196/52713 (PMC11695960; doi:10.2196/52713)
Supplement: Multimedia Appendix 1 [file resprot_v13i1e52713_app1.pdf]

COKIDSS
ENROLMENT CRF FOR SCHOOL PRINCIPAL

Instructions:<br>1. *All instructions are in italics.*		
1	What is the full name of the school?	
2	What is the school postcode?	
3	How many pupils are registered at your school?	
4	How many teachers are employed in your school (including teaching assistants)?	
5	Which year groups do your school include?<br>(*multiple options allowed*)	[1] Nursery (before grade 0)<br>[2] Grade 0<br>[3] Grades 1-2<br>[4] Grades 3-5<br>[5] Grades 6-7<br>[6] Other<br>If other, please specify_________________
6	Please provide number of pupils in each grade.	Nursery(before grade 0) ____________<br>Grade 0 ____________<br>Grade 1 ____________<br>Grade 2 ____________<br>Grade 3 ____________<br>Grade 4 ____________<br>Grade 5 ____________<br>Grade 6 ____________<br>Grade 7 ____________
7	Please provide the number teachers per grade (including teaching assistants)?	Nursery (before grade 0) ___________<br>Grade 0 ____________<br>Grade 1 ____________<br>Grade 2 ____________<br>Grade 3 ____________<br>Grade 4 ____________<br>Grade 5 ____________<br>Grade 6 ____________<br>Grade 7 ____________
8	What is your race (sex) (as assigned at birth)?	[1] Male<br>[2] Female<br>[3] Other, please specify other____________________________<br>[4] Prefer not to answer

9	What is your age?	[1] 25 years and under<br>[2] 26-35 years<br>[3] 36-45 years<br>[4] 46-55 years<br>[5] 56 years or over<br>[6] Prefer not to say
10	What is your ethnic group?	[1] Indian<br>[2] Black/African<br>[3] White<br>[4] Any other mixed/ multiple ethnics background<br>[5] Any other ethnic group (*please specify*): ___________________________<br>[6] Prefer not to say
11	Please provide contact details for the member of staff at your school that we should contact to make arrangements for the study:	Name: ___________________________<br>Email Address: ____________________<br>Contact number: ___________________<br>Alternate contact number:____________

**School opening and implementation of preventive measures**		
**Staff:** *The following questions pertain to the school staff.*		
12	Which of the following preventive measures are **currently being implemented at your school?**	
	Staff stay home if they, or someone in their household, has COVID-19 symptoms	[1] Yes – always<br>[2] Yes – sometimes<br>[3] Never<br>[4] Other<br>If other, please specify_________________
	Staff wear facemasks or face coverings in the classroom	[1] Yes – always<br>[2] Yes – sometimes<br>[3] Never<br>[4] Other<br>If other, please specify_______________
	Staff wear facemasks or face coverings in corridors or communal areas	[1] Yes – always<br>[2] Yes – sometimes<br>[3] Never<br>[4] Other<br>If other, please specify_______________
	Staff wear facemasks or face covering in staff rooms or shared office space	[1] Yes – always<br>[2] Yes – sometimes<br>[3] Never<br>[4] Other

		If other, please specify_______________
	No in-person staff meetings are held	[1] Yes – always<br>[2] Yes – sometimes<br>[3] Never<br>[4] Other<br>If other, please specify_______________
	Teachers stay at the front of the classroom	[1] Yes – always<br>[2] Yes – sometimes<br>[3] Never<br>[4] Other<br>If other, please specify_______________
	Staff regularly wash or sanitize hands	[1] Yes – always<br>[2] Yes – sometimes<br>[3] Never<br>[4] Other<br>If other, please specify_______________
	The same teacher/teaching assistants work with a single class throughout the school week	[1] Yes – always<br>[2] Yes – sometimes<br>[3] Never<br>[4] Other<br>If other, please specify_______________
13	**Learners**<br>*The following questions pertain to the learners.*	
	Learners stay home if they, or someone in their household, has COVID-19 symptoms	[1] Yes – always<br>[2] Yes – sometimes<br>[3] Never<br>[4] Other<br>If other, please specify_______________
	Learners wear face masks or face coverings in the classroom	[1] Yes – always<br>[2] Yes – sometimes<br>[3] Never<br>[4] Other<br>If other, please specify_______________
	Learners wear facemasks or face coverings in corridors or communal areas	[1] Yes – always<br>[2] Yes – sometimes<br>[3] Never<br>[4] Other<br>If other, please specify_______________
	Learners are not offered any after-school activities, including after-care	[0] No<br>[1] Yes

	Daily / weekly temperature checks for Learners	[1] Yes – always<br>[2] Yes – sometimes<br>[3] Never<br>[4] Other<br>If other, please specify_______________
	One-way systems in school corridors	[1] Yes – always<br>[2] Yes – sometimes<br>[3] Never<br>[4] Other<br>If other, please specify_______________
	Hand sanitizers at the school entrance	[1] Yes – always<br>[2] Yes – sometimes<br>[3] Never<br>[4] Other<br>If other, please specify_______________
	No large gatherings of Learners e.g., assemblies	[1] Has been implemented<br>[2] Not applicable<br>[3] Not sure
	Team sports is not allowed	[0] No<br>[1] Yes<br>[2] Not sure
	Learners regularly wash or sanitize hands	[0] No<br>[1] Yes – always<br>[2] Yes - occasionally
14	**Classroom environment:**<br>*The following questions pertain to the classroom environment.*	
	Additional hand sanitizers are provided in classrooms	[0] No<br>[1] Yes
	Learners catch their cough or sneeze with tissue or in their arm	[0] No<br>[1] Yes – always<br>[2] Yes - occasionally
	Seats and desks are spaced apart to maintain distance between Learners	[0] No<br>[1] Yes – in all classrooms<br>[2] Yes – in some classrooms
	Keep all desks facing forward	[0] No<br>[1] Yes – in all classrooms<br>[2] Yes – in some classrooms
	Removing soft furnishings and toys that are hard to clean	[0] No<br>[1] Yes – in all classrooms<br>[2] Yes – in some classrooms

	Increased cleaning of frequently touched surfaces	[0] No<br>[1] Yes
	Scheduling more outdoor lessons and activities	[0] No<br>[1] Yes
	Ensuring learners do not share equipment or learning materials in classrooms	[0] No<br>[1] Yes – in all classrooms<br>[2] Yes – in some classrooms
	Ensuring Learners do not carry equipment or learning materials between home and school	[0] No<br>[1] Yes – in all classrooms/grades<br>[2] Yes – in some classrooms/grades
	Does your school have a policy about the number of COVID-19 cases that would necessitate closure of <u>a class</u>?	[0] No<br>[1] Yes, Please enter number of cases___
	Does your school have a policy about the number of COVID-19 cases that would necessitate closure of <u>a grade group</u>?	[0] No<br>[1] Yes, Please enter number of cases___
	Does your school have any policy about the number of COVID-19 cases that will necessitate closure of <u>the whole school</u>?	[0] No<br>[1] Yes, Please enter number of cases___
	Have you been given guidance about whether your school will be notified about confirmed cases among staff from any of the following sources?	[1] Department for Education<br>[2] Department of Health<br>[3] Local municipality<br>[4] Other, specify ____________________<br>[5] No information received
	Have you been given guidance about whether your school will be notified about confirmed cases among <u>learners</u> from any of the following sources?	[1] Department for Education<br>[2] Department of Health<br>[3] Local municipality<br>[4] Other, specify ____________________<br>[5] No information received
	Have you been given guidance about whether your school will be notified about confirmed cases <u>among household members of Learners</u> from any of the following sources?	[1] Department for Education<br>[2] Department of Health<br>[3] Local municipality<br>[4] Other, specify ____________________<br>[5] No information received
	Have you **<u>so far</u>** been directly informed by any government or public health	[1] Yes, staff case(s)<br>[2] Yes, student case(s)<br>[3] Yes, household member(s) of

	agency about confirmed COVID-19 cases in any of the following groups at your school?	student(s)<br>[4] No, we have not received any information directly from public health agencies about confirmed cases at our school
	Has your school so far used the DfE Helpline for advice on COVID-19?	[0] No<br>[1] Yes

15	In your opinion how has COVID-19 affected teachers in your school?<br><br>*(More than one response allowed)*	[1] Increased anxiety<br>[2] Increased depression<br>[3] Less interest in the work<br>[4] Hopelessness<br>[5] Less commitment<br>[6] Mood changes<br>[7] Increased absenteeism<br>[8] Difficulty concentrating<br>[9] Difficulty focusing<br>[10] More interest in the work<br>[11] More positive energy<br>[12] More commitment<br>[13] No effect<br>[14] Other (specify)
16	In your opinion how has COVID-19 affected learners in your school?	[1] Increased anxiety<br>[2] Increased depression<br>[3] Less interest in the work<br>[4] Hopelessness<br>[5] Less commitment<br>[6] Mood changes<br>[7] Increased absenteeism<br>[8] Difficulty concentrating<br>[9] Difficulty focusing<br>[10] More interest in the work<br>[11] More positive energy<br>[12] More commitment<br>[13] No effect<br>[14] Other (specify)_____________________________

17	In your opinion how has COVID-19 affected parents in your school?	[1] Increased anxiety<br>[2] Increased depression<br>[3] Less interest in the work<br>[4] Hopelessness<br>[5] Less commitment<br>[6] Mood changes<br>[7] Increased absenteeism<br>[8] Difficulty concentrating<br>[9] Difficulty focusing<br>[10] More interest in the work<br>[11] More positive energy<br>[12] More commitment<br>[13] No effect<br>[15] Other (specify)___________________

COKIDSS
FOLLOW-UP CRF FOR SCHOOL PRINCIPAL

1	What is the full name of the school?	
2	How many pupils are currently registered at your school?	
3	How many teachers are currently employed in your school (including teaching assistants)?	
4	Have there been any changes regarding the grades that your school currently has (including teaching assistants)?	[1] Yes<br>[2] No
5	Please provide number of pupils in each grade.	Nursery ___________<br>Grade 0 ___________<br>Grade 1 ___________<br>Grade 2 ___________<br>Grade 3 ___________<br>Grade 4 ___________<br>Grade 5 ___________<br>Grade 6 ___________<br>Grade 7 ___________
6	Please provide the number of teachers per grade (including teaching assistants)?	Nursery ___________<br>Grade 0 ___________<br>Grade 1 ___________<br>Grade 2 ___________<br>Grade 3 ___________<br>Grade 4 ___________<br>Grade 5 ___________<br>Grade 6 ___________<br>Grade 7 ___________
7	Please provide contact details for the member of staff at your school that we should contact to make arrangements for the study:	Name: ____________<br>Email Address: ___________<br>Contact number: _________<br>Alternate contact number:_________________

**School opening and implementation of preventive measures**		
**Staff:**<br>*The following questions pertain to the school staff.*		
8	Which of the following preventive measures are **currently being implemented at your school?**	
	Staff stay home if they, or someone in their household, has COVID-19 symptoms	[1] Yes – always<br>[2] Yes – sometimes<br>[3] Never<br>[4] Other

		If other, please specify_______________
	Staff wear facemasks or face coverings in the classroom	[1] Yes – always<br>[2] Yes – sometimes<br>[3] Never<br>[4] Other<br>If other, please specify_______________
	Staff wear facemasks or face coverings in corridors or communal areas	[1] Yes-always<br>[2] Yes-sometimes<br>[3] Never<br>[4] Other<br>If other, please specify
	Staff wear facemasks or face covering in staff rooms or shared office space	[1] Yes – always<br>[2] Yes – sometimes<br>[3] Never<br>[4] Other<br>If other, please specify_______________
	No in-person staff meetings are held	[1] Yes – always<br>[2] Yes – sometimes<br>[3] Never<br>[4] Other<br>If other, please specify_______________
	Teachers stay at the front of the classroom	[1] Yes – always<br>[2] Yes – sometimes<br>[3] Never<br>[4] Other<br>If other, please specify_______________
	Staff regularly wash or sanitize hands	[1] Yes – always<br>[2] Yes – sometimes<br>[3] Never<br>[4] Other<br>If other, please specify_______________
	The same teacher/teaching assistants work with a single class throughout the school week	[1] Yes – always<br>[2] Yes – sometimes<br>[3] Never<br>[4] Other<br>If other, please specify_______________

9	**Learners:**<br>*The following questions pertain to Learners.*	
	Learners stay home if they, or someone in their household, has COVID-19 symptoms	[1] Yes – always<br>[2] Yes – sometimes

		[3] Never<br>[4] Other<br>If other, please specify_______________
	Learners wear face masks or face coverings in the classroom	[1] Yes – always<br>[2] Yes – sometimes<br>[3] Never<br>[4] Other<br>If other, please specify_______________
	Learners wear facemasks or face coverings in corridors or communal areas	[1] Yes – always<br>[2] Yes – sometimes<br>[3] Never<br>[4] Other<br>If other, please specify_______________
	Not providing after-school activities	[0] No<br>[1] Yes
	Daily / weekly temperature checks for learners	[1] Yes – always<br>[2] Yes – sometimes<br>[3] Never<br>[4] Other<br>If other, please specify_______________
	One-way systems in school corridors	[1] Yes – always<br>[2] Yes – sometimes<br>[3] Never<br>[4] Other<br>If other, please specify_______________
	Hand sanitizers at the school entrance	[1] Yes – always<br>[2] Yes – sometimes<br>[3] Never<br>[4] Other<br>If other, please specify_______________
	No large gatherings of Learners e.g., assemblies	[1] Has been implemented<br>[2] Not sure<br>[99] Not applicable
	Stop team sports	[1] Yes<br>[2] No<br>[3] Not sure
	Learners regularly wash or sanitize hands	[1] Yes – always<br>[2] Yes - occasionally<br>[3] No

10	**Classroom environment**

	The following questions pertain to the classroom environment.	
	Additional hand sanitizers are provided in classrooms	[0] No<br>[1] Yes
	Learners catch their cough or sneeze with tissue or in their arm	[0] No<br>[1] Yes – always<br>[2] Yes - occasionally
	Seats and desks are spaced apart to maintain distance between Learners	[0] No<br>[1] Yes – in all classrooms<br>[2] Yes – in some classrooms
	Keep all desks facing forward	[0] No<br>[1] Yes – in all classrooms<br>[2] Yes – in some classrooms
	Removing soft furnishings and toys that are hard to clean	[0] No<br>[1] Yes – in all classrooms<br>[2] Yes – in some classrooms
	Increased cleaning of frequently touched surfaces	[0] No<br>[1] Yes
	Scheduling more outdoor lessons and activities	[[0] No<br>[1] Yes
	Ensuring learners do not share equipment or learning materials in classrooms	[0] No<br>[1] Yes – in all classrooms<br>[2] Yes – in some classrooms
	Ensuring learners do not carry equipment or learning materials between home and school	[0] No<br>[1] Yes – in all classrooms/grades<br>[2] Yes – in some classrooms/grades
	Does your school have a policy about the number of cases that would necessitate closure of <u>a class</u>?	[0] No<br>[1] Yes, Please enter number of cases______________________
	Does your school have a policy about the number of cases that would necessitate closure of <u>a Grade group</u>?	[0] No<br>[1] Yes, Please enter number of cases_____________________
	Does your school have a policy about the number of cases that would necessitate closure of <u>the whole school?</u>	[0] No<br>[1] Yes, Please enter number of cases____________________

	Have you been given guidance about whether your school will be notified about confirmed cases among staff from any of the following sources?	[1] Department for Education<br>[2] Department of Health<br>[3] Local municipality<br>[4] Other, specify ____________<br>[5] No information received
	Have you been given guidance on whether your school will be notified about confirmed cases among learners from any of the following sources?	[1] Department for Education<br>[2] Department of Health<br>[3] Local municipality<br>[4] Other, specify _____________<br>[5] No information received
	Have you been given guidance about whether your school will be notified about confirmed cases <u>among household members of learners</u> from any of the following sources?	[1] Department for Education<br>[2] Department of Health<br>[3] Local municipality<br>[4] Other, specify ____________<br>[5] No information received
	Have you so far been directly informed by any government or public health agency about confirmed COVID-19 cases in any of the following groups at your school?	[1] Yes, staff case(s)<br>[2] Yes, student case(s)<br>[3] Yes, household member(s) of student(s)<br>[4] No, we have not received any information directly from public health agencies about confirmed cases at our school
	Has your school so far used the DfE Helpline for advice on COVID-19?	[0] No<br>[1] Yes
11	In your opinion how has COVID-19 affected teachers in your school?<br><br>*(More than one response allowed)*	[1] Increased anxiety<br>[2] Increased depression<br>[3] Less interest in the work<br>[4] Hopelessness<br>[5] Less commitment<br>[6] Mood changes<br>[7] Increased absenteeism<br>[8] Difficulty concentrating<br>[9] Difficulty focusing<br>[10] More interest in the work<br>[11] More positive energy<br>[12] More commitment<br>[13] No effect<br>[14] Other (specify)_____________________

12	In your opinion how has COVID-19 affected learners in your school?	[1] Increased anxiety<br>[2] Increased depression<br>[3] Less interest in the work<br>[4] Hopelessness<br>[5] Less commitment<br>[6] Mood changes<br>[7] Increased absenteeism<br>[8] Difficulty concentrating<br>[9] Difficulty focusing<br>[10] More interest in the work<br>[11] More positive energy<br>[12] More commitment<br>[13] No effect<br>[14] Other<br>(specify)____________________
13	In your opinion how has COVID-19 affected parents in your school?	[1] Increased anxiety<br>[2] Increased depression<br>[3] Less interest in the work<br>[4] Hopelessness<br>[5] Less commitment<br>[6] Mood changes<br>[7] Increased absenteeism<br>[8] Difficulty concentrating<br>[9] Difficulty focusing<br>[10] More interest in the work<br>[11] More positive energy<br>[12] More commitment<br>[13] No effect<br>[15] Other<br>(specify)____________________
