# Supplementary material for: SARS-CoV-2 Infections in a Triad of Primary School Learners (Grades 1-7), Their Parents, and Teachers in KwaZulu-Natal, South Africa: Protocol for a Cross-Sectional and Nested Case-Cohort Study
Source: JMIR Res Protoc. 2024 Dec 19;13:e52713. doi: 10.2196/52713 (PMC11695960; doi:10.2196/52713)
Supplement: Multimedia Appendix 2 [file resprot_v13i1e52713_app2.pdf]

COKIDSS CROSS-SECTIONAL SURVEY:
eCRF FOR TEACHER

Instructions:<br>*1. All instructions are in italics.*		
1	Visit code	
2	Do you have a SA identity document (ID) or passport?	[1] SA ID<br>[2] Passport
3	What is your SA ID number or passport number?	
4	Research staff ID<br><br>*This refers to the staff member assisting the participant to complete this CRF.*	
5	Study unique identifier<br><br>*RA must ensure that they have filled/ completed the link log with the study ID and name of participant.*	
6	Re-enter study unique identifier<br>*RA to complete.*	
	Today's date	dd/mm/yyyy
**Enrollment/baseline information: Teacher**		
8	What is your gender (sex as assigned at birth)?	[1] Male<br>[2] Female<br>[3] Other<br>*If other, please specify*______________________________
9	What is your ethnicity (Race)?	[1] Black African<br>[2] Indian<br>[3] Coloured<br>[4] White<br>[5] Other<br>*If other, please specify*________________________________
10	What is your current address (needed for tracing purposes) and suburb?	
11	What is your contact number?<br>*Must input 10 digits.*	
12	Do you have an alternate contact?	[0] No<br>[1] Yes

13	What is your alternate contact number?<br>*Must input 10 digits*	

Information about Teacher's work		
14	What is the full name of the school where you work?	
15	How many days do you work per week in this school?	
16	What is your job at the school?	[1] Teacher<br>[2] Teaching Assistant<br>[3] Other, please specify
17	What grade(s) do you interact with (includes teaching)?<br><br>*Tick all that apply*	[1] Grade 1<br>[2] Grade 2<br>[3] Grade 3<br>[4] Grade 4<br>[5] Grade 5<br>[6] Grade 6<br>[7] Grade 7
18	Is there one class you spend most time with?	[0] No<br>[1] Yes
19	IF Yes, what is the name of that class?	
20	Class ID<br>*Class ID to be CODIFIED.*	
21	Do you spend more time in one or more grades compared to others?	[0] No<br>[1] Yes<br>*If yes, answer question 22.*
22	If Yes, which years?	
23	Do you work at any other school apart from this one (e.g., supply teacher)?	[0] No<br>[1] Yes
24	IF Yes, please list the full names of the other school(s) where you work?	

Teacher: Acute COVID-19 infection				
25	Are you currently feeling sick?			[0] No<br>[1] Yes
26	*Do you have any of the following symptoms now? If YES, indicate which symptoms are currently present, and indicate approximate duration and severity of symptoms.*			
Cough	[0] No<br>[1] Yes	Approximate duration (in days)		Severity of symptoms:<br>[1] I could do

			everything that I usually do<br>[2] I could not do some of what I usually do<br><br>[3] I could not do some of what I usually do most of what I usually do
Sore throat	[0] No<br>[1] Yes	Approximate duration (in days)	Severity of symptoms:<br>[1] I could do everything that I usually do<br>[2] I could not do some of what I usually do<br>[3] I could not do some of what I usually do most of what I usually do
Fever	[0] No<br>[1] Yes	Approximate duration (in days)	Severity of symptoms:<br>[1] I could do everything that I usually do<br>[2] I could not do some of what I usually do<br>[3] I could not do some of what I usually do most of what I usually do
Body ache	[0] No<br>[1] Yes	Approximate duration (in days)	Severity of symptoms:<br>[1] I could do everything

			that I usually do<br>[2] I could not do some of what I usually do<br>[3] I could not do some of what I usually do most of what I usually do
Diarrhea	[0] No<br>[1] Yes	Approximate duration (in days)	Severity of symptoms:<br>[1] I could do everything that I usually do<br>[2] I could not do some of what I usually do<br>[3] I could not do some of what I usually do most of what I usually do
Nausea/vomiting	[0] No<br>[1] Yes	Approximate duration (in days)	Severity of symptoms:<br>[1] I could do everything that I usually do<br>[2] I could not do some of what I usually do<br>[3] I could not do some of what I usually do most of what I usually do
Painful muscles and joints	[0] No<br>[1] Yes	Approximate duration (in days)	Severity of symptoms:<br>[1] I could do everything

			that I usually do<br>[2] I could not do some of what I usually do<br>[3] I could not do some of what I usually do most of what I usually do
Loss of smell	[0] No<br>[1] Yes	Approximate duration (in days)	Severity of symptoms:<br>[1] I could do everything that I usually do<br>[2] I could not do some of what I usually do<br>[3] I could not do some of what I usually do most of what I usually do
Loss of taste	[0] No<br>[1] Yes	Approximate duration (in days)	Severity of symptoms:<br>[1] I could do everything that I usually do<br>[2] I could not do some of what I usually do<br>[3] I could not do some of what I usually do most of what I usually do
Tiredness and fatigue	[0] No<br>[1] Yes	Approximate duration (in days)	Severity of symptoms:<br>[1] I could do everything

			that I usually do<br>[2] I could not do some of what I usually do<br>[3] I could not do some of what I usually do most of what I usually do
Chills	[0] No<br>[1] Yes	Approximate duration (in days)	Severity of symptoms:<br>[1] I could do everything that I usually do<br>[2] I could not do some of what I usually do<br>[3] I could not do some of what I usually do most of what I usually do
Headache	[0] No<br>[1] Yes	Approximate duration (in days)	Severity of symptoms:<br>[1] I could do everything that I usually do<br>[2] I could not do some of what I usually do<br>[3] I could not do some of what I usually do most of what I usually do
Irritability/confusion	[0] No<br>[1] Yes	Approximate duration (in days)	Severity of symptoms:<br>[1] I could do everything

			that I usually do<br>[2] I could not do some of what I usually do<br>[3] I could not do some of what I usually do most of what I usually do
General weakness	[0] No<br>[1] Yes	Approximate duration (in days)	Severity of symptoms:<br>[1] I could do everything that I usually do<br>[2] I could not do some of what I usually do<br>[3] I could not do some of what I usually do most of what I usually do
Skin rash	[0] No<br>[1] Yes	Approximate duration (in days)	Severity of symptoms:<br>[1] I could do everything that I usually do<br>[2] I could not do some of what I usually do<br>[3] I could not do some of what I usually do most of what I usually do

27	When did the first symptoms present?	dd/mm/yyyy
28	**In the last month, did you have close contact with any of the**	A suspected COVID-19 patient<br>[0] No [1] Yes

	**following people:** *Note: Close contact means: face-to-face contact without a mask (≤1 meter) OR been in a closed space with a confirmed case for at least 15 minutes with or without a mask OR lived in the same household OR provided direct care without the recommended PPE.*	A confirmed COVID-19 patient<br>[0] No [1] Yes<br><br>Someone with the “flu” or “cold”<br>[0] No [1] Yes
29	If yes for any of the above, what setting was the contact:<br>(circle or tick or cross all that apply)	[1] Healthcare setting<br>[2] Family setting<br>[3] School setting<br>[4] Public transport setting<br>[5] Other<br><br>*If other, please specify*___________________
30	Did you quarantine after the contact?	[0] No<br>[1] Yes
31	If yes, for how long did you quarantine?	_____days
32	If no, how many contacts did you have since that time?	

**Teacher: Previous History of COVID-19**		
33	Have you been diagnosed with COVID-19 before?	[0] No<br>[1] Yes
34	If yes, how many times?	
35	Which was their most recent time? Give an approximate date of diagnosis	dd/mm/yyyy
36	Over the past 2 years have you felt sick or more tired, or had headaches or lost his/her taste or had COVID-19-like symptoms for 28- days or longer?	[0] No<br>[1] Yes

**Teacher: Long COVID**<br>*(only answer question, if answer to question 36 was yes)*	
37	Tell us which signs/symptoms were present for more than 28-days.
Fatigue/tiredness<br>[0] No<br>[1] Yes	
Stuffy/runny nose<br>[0] No<br>[1] Yes	Chest tightness<br>[0] No<br>[1] Yes
Chest pain<br>[0] No<br>[1] Yes	Cough<br>[0] No<br>[1] Yes

Wheezing<br>[0] No<br>[1] Yes	Sore throat<br>[0] No<br>[1] Yes
Muscle ache<br>[0] No<br>[1] Yes	Joint pain/swelling<br>[0] No<br>[1] Yes
Headache<br>[0] No<br>[1] Yes	Dizziness<br>[0] No<br>[1] Yes
Altered sense of taste (change in taste)<br>[0] No<br>[1] Yes	Altered sense of smell<br>[0] No<br>[1] Yes
Difficulty concentrating (focusing)<br>[0] No<br>[1] Yes	Sleep disorders<br>[0] No<br>[1] Yes
Mood alterations<br>[0] No<br>[1] Yes	Cognitive dysfunction<br>(loss of memory or attentiveness)<br>[0] No<br>[1] Yes
Sensorimotor symptoms (tingling in the toes / feet / legs / fingers / hands or twitching of muscles)<br>[0] No<br>[1] Yes	Increased need for sleep<br>[0] No<br>[1] Yes
Weight loss<br>[0] No<br>[1] Yes	Diarrhea<br>[0] No<br>[1] Yes
Stomach pain<br>[0] No<br>[1] Yes	Poor appetite<br>[0] No<br>[1] Yes
Constipation<br>[0] No<br>[1] Yes	Skin rash<br>[0] No<br>[1] Yes
Tachycardia<br>(fast heart rate)<br>[0] No<br>[1] Yes	Fever<br>[0] No<br>[1] Yes
Other<br>*If other, please specify*____.	

38	If, you have ticked at least one symptom (sign) above. Please select what applies:	[1] A doctor had to be consulted because of this<br>[2] You had to stay away from work<br>*(If applicable answer question 39)*<br>[3] You had to be treated with medication<br>*(If applicable answer question 40)*<br>[4] You had to be hospitalized<br>*(If applicable answer question 41)*<br>[5] You have not done anything<br>[6] Other<br>*If other, please specify*________________________
39	How many days did you have to miss work because of any one or more of these symptoms?	_____*(Please enter number in days)*
40	How many days did you have to be treated with medication because of any one or more of these symptoms?	____*(Please enter number in days)*
41	How many days did you have to spend in the hospital because of any one or more of these symptoms?	____*(Please enter number in days)*

**Teacher: COVID-19 vaccination and vaccination history**		
42	Have you been vaccinated against COVID-19?	[0] No<br>[1] Yes
43	If you have not been vaccinated, we would be interested to know why?<br><br>*More than 1 answer allowed.*	[1] It is a choice and I choose not too<br>[2] No time<br>[3] In general, my family is against all vaccinations<br>[4] No expected benefit (vaccination does not work at all or not enough)<br>[5] I do not trust the vaccine manufacturing companies<br>[6] I do not trust the government's ability to roll out a safe vaccine.<br>[7] I want to wait until there is more knowledge<br>[8]I fear the side effects, safety and effectiveness of vaccinations<br>[9] Due to my religious or cultural beliefs

		[10] I am afraid of needles<br>[11] I had COVID-19, so I do not consider the vaccination necessary<br>[14] Due to my medical condition<br>[15] Other<br>If other, please specify<br>___________________________
43	If yes, when did you receive your first dose?<br>Give an approximate date	dd/mm/yyyy
44	Which vaccine did you receive?<br>(*Please tick the correct answer*)	see table below
5	Date of last dose?	dd/mm/yyyy
46	Do you have any of the following complications?<br>*(Please tick the correct answer)*	

Vaccine table for question 44:

	J&J	0	1	2	
	AstraZeneca	0	1	2	3
	CoronaVac	0	1	2	3
	Moderna	0	1	2	3
	Pfizer	0	1	2	3
	Other (please specify)	0	1	2	3

HIV<br>[0] No<br>[1] Yes	Current TB<br>[0] No<br>[1] Yes	Chronic Kidney Disease<br>[0] No<br>[1] Yes	Chronic Liver Disease<br>[0] No<br>[1] Yes
Neurological/neuromuscular disease<br>[0] No<br>[1] Yes	Diabetes Mellitus<br>[0] No<br>[1] Yes	Heart Disease<br>[0] No<br>[1] Yes	Cancer<br>[0] No<br>[1] Yes
Prior TB infection<br>[0] No<br>[1] Yes	Hypertension (High blood pressure)<br>[0] No<br>[1] Yes	Asthma<br>[0] No<br>[1] Yes	Chronic Lung Disease<br>[0] No<br>[1] Yes
Rheumatological (Joint) disease<br>[0] No<br>[1] Yes	Obesity/overweight<br>[0] No<br>[1] Yes	Autoimmune disease e.g., SLE<br>[0] No<br>[1] Yes	Other 1:<br>Details________________<br>Other 2:<br>Details________________<br>Other 3:<br>Details________________<br>Other 4:<br>Details_________________

47	Are you currently taking any of the following medications NOW:
Steroids<br>(e.g., Prednisone, cortisone)	Anti-inflammatories<br>(e.g., high dose aspirin, ibuprofen)<br>[0] No

[0] No<br>[1] Yes<br>[2] Prefer not to answer	[1] Yes<br>[2] Prefer not to answer
Anti-hypertensives<br>(blood pressure medication e.g., indapamide)<br>[0] No<br>[1] Yes<br>[2] Prefer not to answer	Chemotherapy<br>(cancer treatment)<br>[[0] No<br>[1] Yes<br>[2] Prefer not to answer
Hormonal treatment<br>[0] No<br>[1] Yes<br>[2] Prefer not to answer	Antibiotics<br>(e.g., penicillin, amoxicillin)<br>[0] No<br>[1] Yes<br>[2] Prefer not to answer
ARV/ART<br>[0] No<br>[1] Yes<br>[2] Prefer not to answer	Bactrim prophylaxis<br>[0] No<br>[1] Yes<br>[2] Prefer not to answer
Aspirin / Warfarin / Heparin<br>[0] No<br>[1] Yes<br>[2] Prefer not to answer	TB Meds<br>[0] No<br>[1] Yes<br>[2] Prefer not to answer
Other 1: Details<br>Other 2: Details<br>Other 3: Details	

48	What non-pharmaceutical measures do you currently use to prevent COVID-19?			
	Masks in public places e.g., shops, buses and taxis<br>[0] No<br>[1] Yes always<br>[2] Yes sometimes<br>[99] Not applicable (N/A)	Sanitizing before you enter any classroom or shop<br>[0] No<br>[1] Yes always<br>[2] Yes sometimes<br>[99] N/A	Masks in the workplace<br>[0] No<br>[1] Yes always<br>[2] Yes sometimes<br>[99] N/A	Distancing- - more than 1.5 meters away in the workplace?<br>[0] No<br>[1] Yes always<br>[2] Yes sometimes<br>[99] N/A
	Avoiding social gatherings/outings<br>[0] No<br>[1] Yes always<br>[2] Yes sometimes<br>[99] N/A	Avoiding weddings<br>[0] No<br>[1] Yes always<br>[2] Yes sometimes<br>[99] N/A	Avoiding funerals[0] No<br>[1] Yes always<br>[2] Yes sometimes<br>[99] N/A	

	**Information about Teacher's household**<br>*A household is a group of persons who live together and provide themselves jointly with food and/or other essentials for living, or a single person who lives alone.*<br>*You may spend time living across two households regularly within a week [e.g., house/flat share during the week and another dwelling at the weekend] The following questions relate to your primary household and house, where you spend most of your time:*	
49	In addition to you, how many people are in this household?	
50	Do you spend time living across more than one household within a week?	[0] No<br>[1] Yes
51	IF YES , in addition to you, how many other people live across these	
52	Has anyone in your household been infected with COVID-19 before?	[0] No<br>[1] Yes
53	If yes, how many individuals were infected with COVID-19 before?	
55	Are there any instance when they were all infected around the same time?	[0] No<br>[1] Yes

**Teacher: Specimen collection**<br>*This section is to be completed by study staff collecting the specimens from the Teacher*		
56	Date of specimen collection?	*dd/mm/yyyy*
57	Was blood collected for Rapid COVID-19 antibody POC test?	[0] No<br>[1] Yes<br>[99] N/A
58	If yes, what was the name of the test?	[1] Orient gene<br>[2] Other<br>*If other, please specify*
59	What was the result?<br>*(more than one answer allowed)*	[1] Indeterminate<br>[2] IgG positive<br>[3] IgM positive<br>[4] N/A
60	Was blood collected for DBS?	[0] No<br>[1] Yes<br>[99] N/A
61	Was saliva collected for future testing?	[0] No<br>[1] Yes<br>[99] N/A

FOLLOW-UP SURVEY:
eCRF FOR TEACHER

	Instructions:<br>1. *All instructions are in italics.*	
1	Visit Code	
2	Do you have an SA ID document or passport?	[1] SA ID document<br>[2] Passport
3	What is your SA ID number or passport number?	
4	Research staff ID	*This refers to the staff member assisting the participant to complete this CRF.*
5		
6	Study unique identifier	*The RA will obtain the participant's unique identifier from the link log that was completed in the cross-sectional study.*
7	Re-enter study unique identifier	*RA to complete.*
8	Today's date	dd/mm/yyyy
9	What is the name of the school that you work at?	*The name of the schools should be pre-populated on RedCap. The Teacher merely needs to tick a box for the applicable answer.*
10	What year(s) do you teach?	[1] Grade 1<br>[2] Grade 2<br>[3] Grade 3<br>[4] Grade 4<br>[5] Grade 5<br>[6] Grade 6<br>[7] Grade 7
11	What is the name of the class?	*Class ID to be codified.*

**Teacher: Acute COVID-19 infection**			
12	Are you currently feeling sick?	[0] No<br>[1] Yes	
13	*Do you have any of the following symptoms now? If yes, indicate which symptoms are currently present, and indicate approximate duration and severity of symptoms.*		
Cough	[0] No<br>[1] Yes	*Approximate duration* (in days)	Severity of symptoms today:<br>[1] I could do

			everything that I usually do<br>[2] I could not do some of what I usually do<br>[3] I could not do some of what I usually do most of what I usually do
Sore Throat	[0] No<br>[1] Yes	*Approximate duration* (in days)	Severity of symptoms today:<br>[1] I could do everything that I usually do<br>[2] I could not do some of what I usually do<br>[3] I could not do some of what I usually do most of what I usually do
Fever	[0] No<br>[1] Yes	*Approximate duration* (in days)	Severity of symptoms today:<br>[1] I could do everything that I usually do<br>[2] I could not do some of what I usually do<br>[3] I could not do some of what I usually

			do most of what I usually do
Body ache	[0] No<br>[1] Yes	*Approximate duration* (in days)	Severity of symptoms today:<br>[1] I could do everything that I usually do<br>[2] I could not do some of what I usually do<br>[3] I could not do some of what I usually do most of what I usually do
Diarrhea	[0] No<br>[1] Yes	*Approximate duration* (in days)	Severity of symptoms today:<br>[1] I could do everything that I usually do<br>[2] I could not do some of what I usually do<br>[3] I could not do some of what I usually do most of what I usually do
Nausea/vomiting	[0] No<br>[1] Yes	*Approximate duration* (in days)	Severity of symptoms today:<br>[1] I could do everything that I

			usually do<br>[2] I could not do some of what I usually do<br>[3] I could not do some of what I usually do most of what I usually do
Painful muscle and joints	[0] No<br>[1] Yes	*Approximate duration* (in days)	Severity of symptoms today:<br>[1] I could do everything that I usually do<br>[2] I could not do some of what I usually do<br>[3] I could not do some of what I usually do most of what I usually do
Loss of smell	[0] No<br>[1] Yes	*Approximate duration* (in days)	Severity of symptoms today:<br>[1] I could do everything that I usually do<br>[2] I could not do some of what I usually do<br>[3] I could not do some of what I usually do most of

			what I usually do
Loss of taste	[0] No<br>[1] Yes	*Approximate duration* (in days)	Severity of symptoms today:<br>[1] I could do everything that I usually do<br>[2] I could not do some of what I usually do<br>[3] I could not do some of what I usually do most of what I usually do
Tiredness and fatigue	[0] No<br>[1] Yes	*Approximate duration* (in days)	Severity of symptoms today:<br>[1] I could do everything that I usually do<br>[2] I could not do some of what I usually do<br>[3] I could not do some of what I usually do most of what I usually do
Chills	[0] No<br>[1] Yes	*Approximate duration* (in days)	Severity of symptoms today:<br>[1] I could do everything that I usually do

			[2] I could not do some of what I usually do<br>[3] I could not do some of what I usually do most of what I usually do
Headache	[0] No<br>[1] Yes	*Approximate duration* (in days)	Severity of symptoms today:<br>[1] I could do everything that I usually do<br>[2] I could not do some of what I usually do<br>[3] I could not do some of what I usually do most of what I usually do
Irritability or confusion	[0] No<br>[1] Yes	*Approximate duration* (in days)	Seriousness today:<br>[1] I could do everything that I usually do<br>[2] I could not do some of what I usually do<br>[3] I could not do some of what I usually do most of what I usually do

General weakness	[0] No<br>[1] Yes	*Approximate duration* (in days)	Severity of symptoms today:<br>[1] I could do everything that I usually do<br>[2] I could not do some of what I usually do<br>[3] I could not do some of what I usually do most of what I usually do
Skin rash	[0] No<br>[1] Yes	*Approximate duration* (in days)	Severity of symptoms today:<br>[1] I could do everything that I usually do<br>[2] I could not do some of what I usually do<br>[3] I could not do some of what I usually do most of what I usually do

14	When did your symptoms (signs) first present?	dd/mm/yyyy
15	In the last month, have you had close contact with any of the following people:<br>*Note: Close contact means: face-to-face contact without a mask (≤1 meter) OR been in a closed space with a confirmed case for at least 15 minutes with or without a mask OR lived in the same household OR provided direct care without the recommended PPE:*	A suspected COVID-19 patient<br>[0] No [1] Yes<br>A confirmed COVID-19 patient<br>[0] No [1] Yes<br>Someone with the “flu” or “cold”<br>[0] No [1] Yes

16	If yes for any of the above, what setting was the contact<br>*(Please select the answer that applies)*	[1] Healthcare setting<br>[2] Family setting<br>[3] School setting<br>[4] Public transport setting<br>[5] Other<br>*If other, please specify.*
17	Did you quarantine after the contact?	[0] No<br>[1] Yes
18	If yes, for how long did you quarantine?	days
19	If no, how many contacts have you had since that time?	
20	Have you been diagnosed with COVID-19 before?	[0] No<br>[1] Yes
21	If yes, how many times?	
22	Which was their most recent time? Give an approximate date of diagnosis	dd/mm/yyyy
23	Over the past 2 years have you felt sick or more tired, or had headaches or lost his/her taste or had COVID-19-like symptoms for 28-days or longer?	[0] No<br>[1] Yes

**Teacher: Long COVID**<br>*(Only answer question if answer to question 23 was yes)*	
24	Tell us which signs/symptoms were present for more than 28-days.
Fatigue/tiredness<br>[0] No<br>[1] Yes	
Stuffy/runny nose<br>[0] No<br>[1] Yes	Chest tightness<br>[0] No<br>[1] Yes
Chest pain<br>[0] No<br>[1] Yes	Cough<br>[0] No<br>[1] Yes
Wheezing<br>[0] No<br>[1] Yes	Sore throat<br>[0] No<br>[1] Yes
Muscle ache<br>[0] No<br>[1] Yes	Joint pain/swelling<br>[0] No<br>[1] Yes
Headache<br>[0] No<br>[1] Yes	Dizziness<br>[0] No<br>[1] Yes
Altered sense of taste (change in	Altered sense of smell

taste)<br>[0] No<br>[1] Yes	[0] No<br>[1] Yes
Difficulty concentrating<br>(focusing)<br>[0] No<br>[1] Yes	Sleep disorders<br>[0] No<br>[1] Yes
Mood alterations<br>[0] No<br>[1] Yes	Cognitive dysfunction<br>(loss of memory or attentiveness)<br>[0] No<br>[1] Yes
Sensorimotor symptoms (tingling in the toes / feet / legs / fingers / hands or twitching of muscles)<br>[0] No<br>[1] Yes	Increased need for sleep<br>[0] No<br>[1] Yes
Weight loss<br>[0] No<br>[1] Yes	Diarrhea<br>[0] No<br>[1] Yes
Stomach pain<br>[0] No<br>[1] Yes	Poor appetite<br>[0] No<br>[1] Yes
Constipation<br>[0] No<br>[1] Yes	Skin rash<br>[0] No<br>[1] Yes
Tachycardia<br>(fast heart rate)<br>[0] No<br>[1] Yes	Fever<br>[0] No<br>[1] Yes
Other<br>*If other, please provide space to specify*	

25	If, you have ticked at least one symptom (sign) above. Please select what applies:	[1] A doctor had to be seen because of this<br>[2] You had to stay away from work<br>*(If applicable answer question 26)*<br>[3] You had to be treated with medication<br>*(If applicable answer question 27)*<br>[4] You had to be hospitalized<br>*(If applicable answer question 28)*<br>[5] You have not done anything<br>[6] Other<br>*If other, please specify. Please provide space to specify. (multiple answers possible)*

<table>
<tr><td>26</td><td>How many days did you have to miss work because of any one or more of these symptoms?</td><td>_____(Please enter number in days)</td></tr>
<tr><td>27</td><td>How many days did you have to be treated with medication because of any one or more of these symptoms?</td><td>_____(Please enter number in days)</td></tr>
<tr><td>28</td><td>How many days did you have to spend in the hospital because of any one or more of these symptoms?</td><td>_____(Please enter number in days)</td></tr>
<tr><td colspan="3">Teacher: COVID-19 vaccination and vaccination history</td></tr>
<tr><td>29</td><td>Have you been vaccinated against COVID-19?</td><td>[0] No
[1] Yes</td></tr>
<tr><td>30</td><td>If you have not been vaccinated, we would be interested to know why?</td><td>[1] It is a choice and I choose not too
[2] No time
[3] In general, my family is against all vaccinations
[4] No expected benefit (vaccination does not work at all or not enough)
[5] I do not trust the vaccine manufacturing companies
[6] I do not trust the government’s ability to roll out a safe vaccine.
[7] I want to wait until there is more knowledge
[8]I fear the side effects, safety and effectiveness of vaccinations
[9] Due to my religious or cultural beliefs
[10] I am afraid of needles
[11] I had COVID-19, so I do not consider the vaccination necessary
[14] Due to my medical condition
[15] Other
If other, please specify
Please provide space to specify.

More than 1 answer allowed.</td></tr>
<tr><td>31</td><td>If yes, when did you receive your first dose? Give an approximate date</td><td>dd/mm/yyyy</td></tr>
<tr><td>32</td><td>Which vaccine did you receive?
(Please tick the correct</td><td><table><tr><td></td><td>J&J</td><td>0</td><td>1</td><td>2</td><td></td></tr><tr><td></td><td>AstraZen</td><td>0</td><td>1</td><td>2</td><td>3</td></tr></table></td></tr>
</table>

	answer)		eca				
			CoronaV ac	0	1	2	3
			Moderna	0	1	2	3
			Pfizer	0	1	2	3
			Other (please specify)	0	1	2	3
33	Date of last dose?	dd/mm/yyyy					
34	Do you have any of the following complications?<br>*(Please tick the correct answer)*						

HIV<br>[0] No<br>[1] Yes	Current TB<br>[0] No<br>[1] Yes	Chronic Kidney Disease<br>[0] No<br>[1] Yes	Chronic Liver Disease<br>[0] No<br>[1] Yes
Neurological/neuromuscular disease<br>[0] No<br>[1] Yes	Diabetes Mellitus<br>[0] No<br>[1] Yes	Heart Disease<br>[0] No<br>[1] Yes	Cancer<br>[0] No<br>[1] Yes
Prior TB infection<br>[0] No<br>[1] Yes	Hypertension<br>[0] No<br>[1] Yes	Asthma<br>[0] No<br>[1] Yes	Chronic Lung Disease<br>[0] No<br>[1] Yes
Rheumatological disease<br>[0] No<br>[1] Yes	Obesity/overweight<br>[0] No<br>[1] Yes	Autoimmune disease (e.g., SLE)<br>[0] No<br>[1] Yes	Other 1: Details<br>Other 2: Details<br>Other 3: Details<br>Other 4: Details

35	Are you currently taking any of the following medications NOW:

Steroids<br>(e.g., Prednisone, cortisone)<br>[0] No<br>[1] Yes<br>[2] Prefer not to answer	Anti-inflammatories<br>(e.g., high dose aspirin, ibuprofen)<br>[0] No<br>[1] Yes<br>[2] Prefer not to answer
Anti-hypertensives<br>(blood pressure medication e.g., indapamide)<br>[0] No<br>[1] Yes<br>[2] Prefer not to answer	Chemotherapy<br>(cancer treatment)<br>[0] No<br>[1] Yes<br>[2] Prefer not to answer
Hormonal treatment<br>[0] No<br>[1] Yes<br>[2] Prefer not to answer	Antibiotics<br>(e.g., penicillin, amoxicillin)<br>[0] No<br>[1] Yes<br>[2] Prefer not to answer
ARV/ART	Bactrim prophylaxis

[0] No<br>[1] Yes<br>[2] Prefer not to answer		[0] No<br>[1] Yes<br>[2] Prefer not to answer	
Aspirin / Warfarin / Heparin<br>[0] No<br>[1] Yes<br>[2] Prefer not to answer		TB Meds<br>[0] No<br>[1] Yes<br>[2] Prefer not to answer	
Other 1: Details<br>Other 2: Details<br>Other 3: Details			
36	What non pharmaceutical measures do you currently use to prevent COVID-19?		
Masks in public places e.g., shops, buses and taxis<br>[0] No<br>[1] Yes always<br>[2] Yes sometimes<br>[99] Not applicable (N/A)	Sanitizing before you enter any classroom or shop<br>[0] No<br>[1] Yes always<br>[2] Yes sometimes<br>[99] N/A	Masks in the workplace<br>[0] No<br>[1] Yes always<br>[2] Yes sometimes<br>[99] N/A	Distancing- - more than 1.5 meters away in the workplace?<br>[0] No<br>[1] Yes always<br>[2] Yes sometimes<br>[99] N/A
Avoiding social gatherings/outings<br>[0] No<br>[1] Yes always<br>[2] Yes sometimes<br>[99] N/A	Avoiding weddings<br>[0] No<br>[1] Yes always<br>[2] Yes sometimes<br>[99] N/A	Avoiding funerals<br>[0] No<br>[1] Yes always<br>[2] Yes sometimes<br>[99] N/A	

**Teacher: Specimen collection**<br>*This section is to be completed by study staff collecting the specimens from the Teacher*		
37	Date of specimen collection?	dd/mm/yyyy
38	Was blood collected for Rapid COVID-19 antibody POC test?	[0] No<br>[1] Yes<br>[99] N/A
39	If yes, what was the name of the test?	[1] Orient gene<br>[2] Other<br>*If other, please specify*
40	What was the result?<br>*(more than one answer allowed)*	[1] Indeterminate<br>[2] IgG positive<br>[3] IgM positive<br>[4] N/A
41	Was blood collected for DBS?	[0] No<br>[1] Yes<br>[99] N/A
42	Was saliva collected for future testing?	[0] No<br>[1] Yes<br>[99] N/A
