# Supplementary material for: SARS-CoV-2 Infections in a Triad of Primary School Learners (Grades 1-7), Their Parents, and Teachers in KwaZulu-Natal, South Africa: Protocol for a Cross-Sectional and Nested Case-Cohort Study
Source: JMIR Res Protoc. 2024 Dec 19;13:e52713. doi: 10.2196/52713 (PMC11695960; doi:10.2196/52713)
Supplement: Multimedia Appendix 3 [file resprot_v13i1e52713_app3.pdf]

CROSS-SECTIONAL SURVEY:
eCRF FOR PARENT/LEGAL GUARDIAN OF LEARNER IN GRADE 1-7

<u>*Instructions:*</u><br>*1. All instructions are in italics.*<br>*2. In this study, parent also refers to legal guardian.*		
1	Visit Code	
2	Research staff ID	
3	Does the child have a SA identity document or a passport	[1]_SA identity document<br>[2]Passport
4	What is the child's SA ID number/ Passport Number?	
5	Study unique identifier (learner)<br>*RA must ensure that they have filled/ completed the link log with the study ID and name of participant.*	
6	Re-enter study unique identifier (learner)<br>*RA to complete.*	
7	Do you have an SA identity document or passport?	
8	What is your ID number?	
9	What is your SA ID / Passport number ?	[1]_SA identity document<br>[2]Passport
10	Study unique identifier (Parent)<br>*RA must ensure that they have filled/ completed the link log with the study ID and name of participant.*	
11	Re-enter study unique identifier (Parent)<br>*RA to complete.*	
12	Today's date	dd/mm/yyyy
**Enrollment/baseline Information of the parent/guardian**		
13	What is the name of school your child attends?	.
14	What grade is your child in at school?	[1] Grade 1<br>[2] Grade 2<br>[3] Grade 3<br>[4] Grade 4<br>[5] Grade 5<br>[6] Grade 6<br>[7] Grade 7
15	What is the name of your child's class?<br>*Class ID to be CODIFIED.*	

16	What is your relationship to the child?	[1] Parent<br>[2] Guardian
17	If your relationship is parent, are you the?	[1] Mother<br>[2] Father<br>[3] Stepmother<br>[4] Stepfather<br>[5] Foster mother<br>[6] Foster father
18	If your relationship is guardian, are you the?	[1] Grand mother<br>[2] Grand father<br>[3] Aunt<br>[4] Uncle<br>[5] Older Sister<br>[6] Older Brother<br>[7] Older Stepsister<br>[8] Older Stepbrother<br>[9] Cousin<br>[10] Other relative, please specify<br>[11] No relation, please specify<br>________________________
19	What is your gender (sex)?	[1] Male<br>[2] Female<br>[3] Other<br>*If other, please specify*____________
20	What is your ethnicity (race)?	[1] Black African<br>[2] Indian<br>[3] Coloured<br>[4] White<br>[5] Other<br>*If other, please specify.*________________
21	What is your current address and suburb?<br>*Needed for tracing purposes*	
22	What is your contact number?<br>*Must input 10 digits.*	
23	What is your alternate contact number?<br>*Must input 10 digits.*	

24	What is your highest level of education?	[1] No formal education<br>[2] Junior primary school (Grade 1-4)<br>[3] Senior primary school (Grade 5-7)<br>[4] Some secondary school (Grade 8-12)<br>[5] Completed secondary school with certificate (Grade 12)<br>[6] Some university/technical education<br>[7] Completed university/technical education<br>[8] National certificate/trade
25	What is your current work situation?	[1] Employed, part-time<br>[2] Employed, full-time<br>[2] Unemployed<br>[3] Other<br>*If other, please provide space to specify.*

**Parent/Guardian: Acute COVID-19 infection**			
26	Are you currently feeling sick**?**	[1] Yes<br>[0] No	
27	Do you have any of the following symptoms now? If yes, indicate which symptoms are currently present, and indicate approximate duration and severity		
Cough	[1] Yes<br>[0] No	*Approximate duration (in days)*	Seriousness today:<br>[1] I could do everything that I usually do<br>[2] I could not do some of what I usually do<br>[3] I could not do some of what I usually do most of

			what I usually do
Sore Throat	[1] Yes<br>[0] No	*Approximate duration (in days)*	Seriousness today:<br>[1] I could do everything that I usually do<br>[2] I could not do some of what I usually do<br>[3] I could not do some of what I usually do most of what I usually do
Fever	[1] Yes<br>[0] No	*Approximate duration (in days)*	Seriousness today:<br>[1] I could do everything that I usually do<br>[2] I could not do some of what I usually do<br>[3] I could not do some of what I usually do most of what I usually do
Body ache	[1] Yes<br>[0] No	*Approximate duration (in days)*	Seriousness today:<br>[1] I could do everything that I usually do<br>[2] I could not do some of what I usually do<br>[3] I could not do some of what I usually do most of what I usually do
Diarrhea	[1] Yes<br>[0] No	*Approximate duration (in days)*	Seriousness today:<br>[1] I could do

			everything that I usually do<br>[2] I could not do some of what I usually do<br>[3] I could not do some of what I usually do most of what I usually do
Nausea/vomiting	[1] Yes<br>[0] No	*Approximate duration (in days)*	Seriousness today:<br>[1] I could do everything that I usually do<br>[2] I could not do some of what I usually do<br>[3] I could not do some of what I usually do most of what I usually do
Painful muscle and joints	[1] Yes<br>[0] No	*Approximate duration (in days)*	Seriousness today:<br>[1] I could do everything that I usually do<br>[2] I could not do some of what I usually do<br>[3] I could not do some of what I usually do most of what I usually do
Loss of smell	[1] Yes<br>[0] No	*Approximate duration (in days)*	Seriousness today:<br>[1] I could do everything that I usually do<br>[2] I could not do some of

			what I usually do<br>[3] I could not do some of what I usually do most of what I usually do
Loss of taste	[1] Yes<br>[0] No	*Approximate duration (in days)*	Seriousness today:<br>[1] I could do everything that I usually do<br>[2] I could not do some of what I usually do<br>[3] I could not do some of what I usually do most of what I usually do
Tiredness and fatigue	[1] Yes<br>[0] No	*Approximate duration (in days)*	Seriousness today:<br>[1] I could do everything that I usually do<br>[2] I could not do some of what I usually do<br>[3] I could not do some of what I usually do most of what I usually do
Chills	[1] Yes<br>[0] No	*Approximate duration (in days)*	Seriousness today:<br>[1] I could do everything that I usually do<br>[2] I could not do some of what I usually do<br>[3] I could not do some of what I usually do most of

			what I usually do
Headache	[1] Yes<br>[0] No	*Approximate duration (in days)*	Seriousness today:<br>[1] I could do everything that I usually do<br>[2] I could not do some of what I usually do<br>[3] I could not do some of what I usually do most of what I usually do
Irritability or confusion	[1] Yes<br>[0] No	*Approximate duration (in days)*	Seriousness today:<br>[1] I could do everything that I usually do<br>[2] I could not do some of what I usually do<br>[3] I could not do some of what I usually do most of what I usually do
General weakness	[1] Yes<br>[0] No	*Approximate duration (in days)*	Seriousness today:<br>[1] I could do everything that I usually do<br>[2] I could not do some of what I usually do<br>[3] I could not do some of what I usually do most of what I usually do
Skin rash	[1] Yes<br>[0] No	*Approximate duration (in days)*	Seriousness today:<br>[1] I could do everything

			that I usually do<br>[2] I could not do some of what I usually do<br>[3] I could not do some of what I usually do most of what I usually do

28	When did the symptoms first start?	dd/mm/yyyy
29	**In the last month, did you have close contact with any of the following people:** *Note: Close contact means: face-to-face contact without a mask (≤1 meter) OR been in a closed space with a confirmed case for at least 15 minutes with or without a mask OR lived in the same household OR provided direct care without the recommended PPE.*	A suspected COVID-19 patient<br>[1] Yes [0] No<br><br>A confirmed COVID-19 patient<br>[1] Yes [0] No<br><br>Someone with the “flu” or “cold”<br>[1] Yes [0] No
30	If yes for any of the above, what setting was the contact:<br>(Select or cross all that apply)	[1] Healthcare setting<br>[2] Family setting<br>[3] School setting<br>[4] Public transport setting<br>[5] Other<br>If other, please specify.
31	Did you quarantine after the contact?	[1] Yes<br>[0] No
32	If yes, for how long did you quarantine?	_____days
33	If no, how many contacts did you have since that time?	

**Previous history of COVID-19**		
34	Have you been diagnosed with COVID-19 before?	[1] Yes<br>[0] No
35	If yes, how many times?	
36	Which was your most recent time? *Give an approximate date*	dd/mm/yyyy
37	Over the past 2 years have you felt sick or more tired, or had headaches or lost his/her taste or had COVID-19-like symptoms for 28- days or longer?	[1] Yes<br>[0] No
**Parent/ Guardian: Long COVID**<br>*(only answer question if answer to question 37 was yes)*		
38	Indicate which symptoms were present for more than 28-days.<br>Tell us which signs / symptoms were present for more tha28-days	

Fatigue<br>[1] Yes<br>[0] No	
Stuffy/runny nose<br>[1] Yes<br>[0] No	Chest tightness<br>[1] Yes<br>[0] No
Chest pain<br>[1] Yes<br>[0] No	Cough<br>[1] Yes<br>[0] No
Wheezing<br>[1] Yes<br>[0] No	Sore throat<br>[1] Yes<br>[0] No
Muscle ache<br>[1] Yes<br>[0] No	Joint pain/swelling<br>[1] Yes<br>[0] No
Headache<br>[1] Yes<br>[0] No	Dizziness<br>[1] Yes<br>[0] No
Altered sense of taste (change in taste)<br>[1] Yes<br>[0] No	Altered sense of smell<br>[1] Yes<br>[0] No
Difficulty concentrating<br>(focusing)<br>[1] Yes<br>[0] No	Sleep disorders<br>[1] Yes<br>[0] No
Mood alterations<br>[1] Yes<br>[0] No	Cognitive dysfunction<br>(loss of memory or difficulty processing information or paying attention)<br>[1] Yes<br>[0] No
Sensorimotor symptoms (tingling in the toes / feet / legs / fingers / hands or twitching of muscles)<br>[1] Yes<br>[0] No	Increased need for sleep<br>[1] Yes<br>[0] No
Weight loss<br>[1] Yes<br>[0] No	Diarrhea<br>[1] Yes<br>[0] No
Stomach pain<br>[1] Yes<br>[0] No	Poor appetite<br>[1] Yes<br>[0] No
Constipation<br>[1] Yes<br>[0] No	Skin rash<br>[1] Yes<br>[0] No
Tachycardia	Fever

(fast heart rate)<br>[1] Yes<br>[0] No		[1] Yes<br>[0] No
Other<br>*If other, please specify.*<br>*Please provide space to specify*		
38	If, you have selected at least one symptom (sign) above. Please select what applies:	[1] A doctor had to be seen because of this<br>[2] You had to stay away from work<br>*(If applicable answer question 39)*<br>[3] You had to be treated with medication<br>*(If applicable answer question 40)*<br>[4] You had to be hospitalized<br>*(If applicable answer question 41)*<br>[5] You have not done anything<br>[6] Other<br>*If other, please specify. Please provide space to specify. (multiple answers possible)*
39	How many days did you have to miss?	_____*(Please enter number in days)*
40	How many days did you have to be treated with medication?	_____*(Please enter number in days)*
41	How many days did you have to spend in the hospital?	_____*(Please enter number in days)*
**Parent/Guardian: COVID-19 vaccination and vaccination history**		
42	Have you been vaccinated against COVID-19?	[1] Yes<br>[0] No
43	If you have not been vaccinated, we would be interested to know why?	[1] It is a choice and I choose not too<br>[2] No time<br>[3] In general, my family is against all vaccinations<br>[4] No expected benefit (vaccination does not work at all or not enough)<br>[5] I do not trust the vaccine manufacturing companies<br>[6] I do not trust the government's ability to roll out a safe vaccine.<br>[7] I want to wait until there is more knowledge

		[8]I fear the side effects, safety and effectiveness of vaccinations<br>[9] Due to my religious or cultural beliefs<br>[10] I am afraid of needles<br>[11] I had COVID-19, so I do not consider the vaccination necessary<br>[14] Due to my medical condition<br>[15] Other<br>If other, please specify<br>*Please provide space to specify.*<br><br>*More than 1 answer allowed.*
44	If yes, when did you receive the first dose? Give an approximate date	dd/mm/yyyy
45	Which vaccine did you receive?<br>(*Please select the correct answer*)	see vaccine table below
46	Date of last dose?	dd/mm/yyyy
47	Do you have any of the following complications?<br>*(Please select the correct answer)*	

	J&J	0	1	2	
	AstraZeneca	0	1	2	3
	CoronaVac	0	1	2	3
	Moderna	0	1	2	3
	Pfizer	0	1	2	3
	Other (please specify)	0	1	2	3

HIV<br>[1] Yes<br>[0] No	Current TB<br>[1] Yes<br>[0] No	Chronic Kidney Disease<br>[1] Yes<br>[0] No	Chronic Liver Disease<br>[1] Yes<br>[0] No
Neurological/neuromuscular disease<br>[1] Yes<br>[0] No	Diabetes Mellitus (high blood sugar)<br>[1] Yes<br>[0] No	Heart Disease<br>[1] Yes<br>[0] No	Cancer<br>[1] Yes<br>[0] No
Prior TB infection<br>[1] Yes<br>[0] No	Hypertension (high blood pressure)<br>[1] Yes<br>[0] No	Asthma (Difficulty breathing)<br>[1] Yes<br>[0] No	Chronic Lung Disease<br>[1] Yes<br>[0] No
Rheumatological disease (disease of the joints and muscles)<br>[1] Yes<br>[0] No	Obesity/overweight<br>[1] Yes<br>[0] No	Autoimmune disease (not HIV. A disease whereby our immune system starts attacking	Other 1:<br>Details<br>Other 2:<br>Details<br>Other 3:

		our own tissues or organs e.g., SLE)<br>[1] Yes<br>[0] No	Details<br>Other 4: Details

48	Are you currently taking any of the following medications NOW:
Steroids<br>(e.g., Prednisone, cortisone)<br>[0] No<br>[1] Yes<br>[2] Prefer not to answer	Anti-inflammatories<br>(e.g., high dose aspirin, ibuprofen)<br>[0] No<br>[1] Yes<br>[2] Prefer not to answer
Anti-hypertensives<br>(blood pressure medication e.g., indapamide)<br>[0] No<br>[1] Yes<br>[2] Prefer not to answer	Chemotherapy<br>(cancer treatment)<br>[0] No<br>[1] Yes<br>[2] Prefer not to answer
Hormonal treatment<br>[0] No<br>[1] Yes<br>[2] Prefer not to answer	Antibiotics<br>(e.g., penicillin, amoxicillin)<br>[0] No<br>[1] Yes<br>[2] Prefer not to answer
ARV/ART<br>[0] No<br>[1] Yes<br>[3] Prefer not to answer	Bactrim prophylaxis<br>[0] No<br>[1] Yes<br>[2] Prefer not to answer
Aspirin / Warfarin / Heparin<br>[0] No<br>[1] Yes<br>[2] Prefer not to answer	TB Meds<br>[0] No<br>[1] Yes<br>[2] Prefer not to answer
Other 1: Details<br>Other 2: Details<br>Other 3: Details	

47	What non pharmaceutical measures do you currently use to prevent COVID-19?		
Masks in public places e.g., buses and taxis<br>[0] No<br>[1] Yes always<br>[2] Yes sometimes<br>[99] Not applicable (N/A)	Sanitizing<br>[0] No<br>[1] Yes always<br>[2] Yes sometimes<br>[99] N/A	Masks in the workplace<br>[0] No<br>[1] Yes always<br>[2] Yes sometimes<br>[99] N/A	Distancing- - more than 1.5 meters away in the workplace?<br>[0] No<br>[1] Yes always<br>[2] Yes sometimes

			[99] N/A
Avoiding social gatherings/outings<br>[0] No<br>[1] Yes always<br>[2] Yes sometimes<br>[99] N/A	Avoiding weddings<br>[0] No<br>[1] Yes always<br>[2] Yes sometimes<br>[99] N/A	Avoiding funerals<br>[0] No<br>[1] Yes always<br>[2] Yes sometimes<br>[99] N/A	

**Parent/Guardian: Specimen collection**<br>*This section is to be completed by study staff collecting the specimens from the parent/legal guardian*		
49	Date of specimen collection?	dd/mm/yyyy
50	Was blood collected for Rapid COVID-19 antibody POC test?	[0] No<br>[1] Yes<br>[99] N/A
51	If yes, what was the name of the test?	[1] Orient gene<br>[2] Other<br>*If other, please specify*
52	What was the result?<br>*(more than one answer allowed)*	[1] Indeterminate<br>[2] IgG positive<br>[3] IgM positive<br>[99] N/A
53	Was blood collected for DBS?	[0] No<br>[1] Yes<br>[99] N/A
54	Was saliva collected for future testing?	[0] No<br>[1] Yes<br>[99] N/A

**Enrolment/baseline information for learner**		
55	What is the learner's gender (sex)?	[1] Male<br>[2] Female<br>[3] Other<br>*If other, please specify__________________.*
56	What is the learner's ethnicity (race)?	[1] Black African<br>[2] Indian<br>[3] Coloured<br>[4] White<br>[5] Other<br>*If other, please specify______________________.*

57	What is the learner's current address (needed for tracing purposes) and suburb?<br>*This address needs to match the parent's/legal guardian's address*	
58	How was the learner fed when they were a baby?	[1] Exclusively breastfed (no other food or fluids)<br>[2] Exclusively formula fed (no breastmilk)<br>[3] Mixed fed (breastmilk and formula fed)<br>[4] Unknown
59	If the learner was exclusively breastfed for any period of time, for how long?	_________months
60	If the learner was breastfed, for how long?	_________months
	**Learner: Acute COVID-19 infection**	
61	Is the learner currently feeling sick?	[0] No<br>[1] Yes
62	Does the learner have any of the following symptoms now? If yes, indicate which symptoms are currently present, and indicate approximate duration and severity	

Cough	[0] No<br>[1] Yes	*Approximate duration (in days)*	Seriousness today:<br>[1] I could do everything that I usually do<br>[2] I could not do some of what I usually do<br>[3] I could not do some of what I usually do most of what I usually do
Sore Throat	[0] No<br>[1] Yes	*Approximate duration (in days)*	Seriousness today:<br>[1] I could do everything that I usually do<br>[2] I could not do some of what I usually do<br>[3] I could not do some of what I usually do most of what I usually do
Fever	[0] No<br>[1] Yes	*Approximate duration (in days)*	Seriousness today:<br>[1] I could do

			everything that I usually do<br>[2] I could not do some of what I usually do<br>[3] I could not do some of what I usually do most of what I usually do
Body ache	[0] No<br>[1] Yes	*Approximate duration (in days)*	Seriousness today:<br>[1] I could do everything that I usually do<br>[2] I could not do some of what I usually do<br>[3] I could not do some of what I usually do most of what I usually do
Diarrhea	[0] No<br>[1] Yes	*Approximate duration (in days)*	Seriousness today:<br>[1] I could do everything that I usually do<br>[2] I could not do some of what I usually do<br>[3] I could not do some of what I usually do most of what I usually do
Nausea/vomiting	[0] No<br>[1] Yes	*Approximate duration (in days)*	Seriousness today:<br>[1] I could do everything that I usually do<br>[2] I could not do some of what I usually do<br>[3] I could not do some of what I usually do most of what I usually do

Painful muscle and joints	[0] No<br>[1] Yes	*Approximate duration (in days)*	Seriousness today:<br>[1] I could do everything that I usually do<br>[2] I could not do some of what I usually do<br>[3] I could not do some of what I usually do most of what I usually do
Loss of smell	[0] No<br>[1] Yes	*Approximate duration (in days)*	Seriousness today:<br>[1] I could do everything that I usually do<br>[2] I could not do some of what I usually do<br>[3] I could not do some of what I usually do most of what I usually do
Loss of taste	[0] No<br>[1] Yes	*Approximate duration (in days)*	Seriousness today:<br>[1] I could do everything that I usually do<br>[2] I could not do some of what I usually do<br>[3] I could not do some of what I usually do most of what I usually do
Tiredness and fatigue	[0] No<br>[1] Yes	*Approximate duration (in days)*	Seriousness today:<br>[1] I could do everything that I usually do<br>[2] I could not do some of what I usually do<br>[3] I could not do some of what I usually do most of what I usually

			do
Chills	[0] No<br>[1] Yes	*Approximate duration (in days)*	Seriousness today:<br>[1] I could do everything that I usually do<br>[2] I could not do some of what I usually do<br>[3] I could not do some of what I usually do most of what I usually do
Headache	[0] No<br>[1] Yes	*Approximate duration (in days)*	Seriousness today:<br>[1] I could do everything that I usually do<br>[2] I could not do some of what I usually do<br>[3] I could not do some of what I usually do most of what I usually do
Irritability or confusion	[0] No<br>[1] Yes	*Approximate duration (in days)*	Seriousness today:<br>[1] I could do everything that I usually do<br>[2] I could not do some of what I usually do<br>[3] I could not do some of what I usually do most of what I usually do
General weakness	[0] No<br>[1] Yes	*Approximate duration (in days)*	Seriousness today:<br>[1] I could do everything that I usually do<br>[2] I could not do some of what I usually do<br>[3] I could not do some of what I usually do most

			of what I usually do
Skin rash	[0] No<br>[1] Yes	*Approximate duration (in days)*	Seriousness today:<br>[1] I could do everything that I usually do<br>[2] I could not do some of what I usually do<br>[3] I could not do some of what I usually do most of what I usually do

63	When did the learner's symptoms (signs) first present?	dd/mm/yyyy
64	**In the last month, has the learner had close contact with someone with suspected COVID-19, confirmed COVID-19, flu or a cold?**<br>*Note: Close contact means the learner ate with or played with this person. They were close together for at least 15 minutes*	A suspected COVID-19 patient<br>[0] No [1] Yes…<br>A confirmed COVID-19 patient<br>[0] No [1] Yes…<br>Someone with the "flu" or "cold"<br>[1] Yes…[2] No
65	If yes for any of the above, what setting was the contact:<br>*(Please select the answer that applies)*	[1] Healthcare setting<br>[2] Family setting<br>[3] School setting<br>[4] Public transport setting<br>[5] Other<br>*If other, please specify.*
66	Did the learner quarantine after the contact?	[0] No<br>[1] Yes
67	If yes, for how long did the learner quarantine?	____________days
68	If no, how many contacts did the learner have since that time?	
	**Previous history of COVID-19**	
69	Has the learner been diagnosed with COVID-19 before?	[0] No<br>[1] Yes
70	If yes, how many times?	
71	Which was their most recent time? Give an approximate date of diagnosis	dd/mm/yyyy
72	Over the past 2 years has the learner felt sick or more tired, or had headaches or lost his/her taste or had COVID-19-like symptoms for 28-days or longer?	[0] No<br>[1] Yes

**Learner: Long COVID**<br>*(Only answer question, if answer to question 72 was yes)*	
73	Tell us which symptoms were present for more than 28-days.
Fatigue<br>[0] No<br>[1] Yes	
Stuffy/runny nose<br>[0] No<br>[1] Yes	Chest tightness<br>[0] No<br>[1] Yes
Chest pain<br>[0] No<br>[1] Yes	Cough<br>[0] No<br>[1] Yes
Wheezing<br>[0] No<br>[1] Yes	Sore throat<br>[0] No<br>[1] Yes
Muscle ache<br>[0] No<br>[1] Yes	Joint pain/swelling<br>[0] No<br>[1] Yes
Headache<br>[0] No<br>[1] Yes	Dizziness<br>[0] No<br>[1] Yes
Altered sense of taste (change in taste)<br>[0] No<br>[1] Yes	Altered sense of smell<br>[0] No<br>[1] Yes
Difficulty concentrating (focusing)<br>[0] No<br>[1] Yes	Sleep disorders<br>[0] No<br>[1] Yes
Mood alterations<br>[0] No<br>[1] Yes	Cognitive dysfunction<br>(loss of memory or difficulty processing information or paying attention)<br>[0] No<br>[1] Yes
Sensorimotor symptoms (tingling in the toes / feet / legs / fingers / hands or twitching of muscles)<br>[0] No<br>[1] Yes	Increased need for sleep<br>[] No<br>[1] Yes
Weight loss<br>[0] No<br>[1] Yes	Diarrhea<br>[0] No<br>[1] Yes
Stomach pain	Poor appetite

[0] No<br>[1] Yes	[0] No<br>[1] Yes
Constipation<br>[0] No<br>[1] Yes	Skin rash<br>[0] No<br>[1] Yes
Tachycardia<br>(fast heart rate)<br>[0] No<br>[1] Yes	Fever<br>[2] No<br>[1] Yes
Other<br>*If other, please specify.*<br>*Please provide space to specify*	

74	If, you have selected at least one symptom (sign) above. Please select what applies:	[1] A doctor had to be consulted because of this<br>[2] He/she had to stay away from school<br>*(If applicable answer question 75)*<br>[3] He/she had to be treated with medication<br>*(If applicable answer question 76)*<br>[4] He/she had to be hospitalized<br>*(If applicable answer question 77)*<br>[5] He/she has not done anything<br>[6] Other<br>*If other, please specify.*<br>*Please provide space to specify.*<br>*(multiple answers possible)*
75	How many days did the learner have to miss school?	____*(Please enter number in days)*
76	How many days did the learner have to be treated with medication?	____*(Please enter number in days)*
77	How many days did the learner have to spend in the hospital?	____*(Please enter number in days)*

**Learner (≥ 12 years of age): COVID-19 vaccination and vaccine hesitancy**<br>*These questions are applicable for learners that are eligible for the COVID-19 vaccination (≥ 12 years of age)*

78	Has the learner been vaccinated against COVID-19?	[0] No<br>[1] Yes
79	If the learner has not been vaccinated, we would be interested to know why not?<br><br>*More than 1 answer allowed.*	1] It is a choice and the learner or learner's parent/legal guardian choose not too<br>[2] No time<br>[3] In general, the learner's family is against all vaccinations<br>[4] No expected benefit (vaccination does not work at all or not sufficiently)<br>[5] The learner or learner's parent/legal guardian does not trust the vaccine manufacturing companies<br>[6] The learner or learner's parent/legal guardian does not trust the government's ability to roll out a safe vaccine.<br>[7] The learner or learner's parent/legal guradian wants to wait until there is more knowledge<br>[8] The learner or learner's parent/legal guardian fear the side effects, safety and effectiveness of vaccinations<br>[9] Due to the learner or learner's parentt/legal guardian religious or cultural beliefs<br>[10] The learner is afraid of needles<br>[11] The learner had COVID-19, so I do not consider the vaccination necessary<br>[13] The learner's parent/legal guardian just won't let them take it.<br>[14] Due to the learner's medical condition<br>[15] Other<br>If other, please specify<br>*Please provide space to specify.*

80	If yes, when did the learner receive their first dose? Give an approximate date	dd/mm/yyyy
81	Which vaccine did the learner receive?	[1] Pfizer<br>[2] Sinovac (Coronac)<br>[3] Other (specify)<br>[4] Do not know
82	How many doses did the learner receive?<br>*Circle the correct answer*	(see dose table below)
83	Date of last (most recent) dose?	dd/mm/yyyy

	Pfizer	0	1	2	3
	Sinovac (Coronac)	0	1	2	3
	Other (specify)	0			

Learner: Chronic illness

84 Does the learner have any of the following complications?

HIV<br>[0] No<br>[1] Yes	Current TB<br>[0] No<br>[1] Yes	Chronic Kidney Disease<br>[0] No<br>[1] Yes	Chronic Liver Disease<br>[0] No<br>[1] Yes
Neurological/neuromuscular disease<br>[0] No<br>[1] Yes	Diabetes Mellitus (high blood sugar)<br>[0] No<br>[1] Yes	Heart Disease<br>[0] No<br>[1] Yes	Cancer<br>[0] No<br>[1] Yes
Prior TB infection<br>[0] No<br>[1] Yes	Hypertension (high blood pressure)<br>[0] No<br>[1] Yes	Asthma (Difficulty breathing)<br>[0] No<br>[1] Yes	Chronic Lung Disease<br>[0] No<br>[1] Yes
Rheumatological disease (disease of the joints and muscles)<br>[0] No<br>[1] Yes	Obesity/overweight<br>[0] No<br>[1] Yes	Autoimmune disease (not HIV. A disease whereby our immune system starts attacking our own tissues or organs e.g., SLE)<br>[0] No<br>[1] Yes	Other 1: Details<br>Other 2: Details<br>Other 3: Details<br>Other 4: Details

85 Is the learner currently taking any of the following medications NOW:

Steroids<br>(e.g., Prednisone, cortisone)<br>[0] No	Anti-inflammatories<br>(e.g., high dose aspirin, ibuprofen)<br>[0] No<br>[1] Yes

[1] Yes<br>[2] Prefer not to answer	[2] Prefer not to answer
Anti-hypertensives<br>(blood pressure medication e.g., indapamide)<br>[0] No<br>[1] Yes<br>[2] Prefer not to answer	Chemotherapy<br>(cancer treatment)<br>[0] No<br>[1] Yes<br>[2] Prefer not to answer
Hormonal treatment<br>[0] No<br>[1] Yes<br>[2] Prefer not to answer	Antibiotics<br>(e.g., penicillin, amoxicillin)<br>[0] No<br>[1] Yes<br>[2] Prefer not to answer
ARV/ART<br>[0] No<br>[1] Yes<br>[2] Prefer not to answer	Bactrim prophylaxis<br>[0] No<br>[1] Yes<br>[2] Prefer not to answer
Aspirin / Warfarin / Heparin<br>[0] No<br>[1] Yes<br>[2] Prefer not to answer	TB Meds<br>[0] No<br>[1] Yes<br>[2] Prefer not to answer
Other 1: Details<br>Other 2: Details<br>Other 3: Details	

86	What non pharmaceutical measures does the learner currently use to prevent COVID-19?		
Masks in public places e.g., buses and taxis<br>[0] No<br>[1] Yes always<br>[2] Yes – sometimes<br>[99] N/A	Sanitizing<br>[0] No<br>[1] Yes always<br>[2] Yes – sometimes<br>[99] N/A	Masks in the school setting?<br>[0] No<br>[1] Yes always<br>[2] Yes – sometimes<br>[99] N/A	Distancing- - more than 1.5 meters away in the school setting?<br>[0] No<br>[1] Yes always<br>[2] Yes – sometimes<br>[99] N/A
Avoiding social gatherings/outings<br>[0] No<br>[1] Yes always<br>[2] Yes – sometimes<br>[99] N/A	Avoiding weddings<br>[0] No<br>[1] Yes always<br>[2] Yes – sometimes<br>[99] N/A	Avoiding funerals<br>[0] No<br>[1] Yes always<br>[2] Yes – sometimes<br>[99] N/A	
**Information about people in the learner's house**			

A household is a group of persons who live together and provide themselves jointly with food and/or other essentials for living, or a single person who lives alone.<br><br>We would like to ask some question about your child's household(s) and household members. The following question help in understanding the importance of people's living circumstances related to COVID-19.<br>By household, we mean the group of people your child lives with, in a single house or dwelling.		
87	Does the learner spend time living across more than one household within a week?	[0] No<br>[1] Yes
Your child may spend time living across two households regularly within a week. The following questions relate to their primary household and house, where your child spends most of their time:		
88	In addition to the learner, how many people are in this household?	
89	How many rooms does the learner's house have in total (please exclude any kitchens or bathrooms)?	
90	How many rooms in the learner's house are regularly used for sleeping?	
Could you please provide us with some details including any history of COVID- 19-like symptoms (signs) among other household members (excluding {child's name}). The information will help us understand the circumstances around your child's test results. *PLEASE PROCEED FROM THE YOUNGEST TO THE OLDEST MEMBER OF THE HOUSEHOLD;* ***If the child lives between more than one household, please also provide details for members of all households:***		
	**Household Member 1:**<br>*In RedCap, this section must be repeated to allow for up to 10 household members to be inserted.*	
91	Relationship to learner:	[1] Spouse/Partner<br>[2] Child/Stepchild<br>[3] Mother/Mother-in-law<br>[4] Father/Father-in-law<br>[5] Brother/Stepbrother<br>[6] Sister/Stepsister<br>[7]Grand parent<br>[9] Aunt<br>[10] Uncle<br>[11] Other relative, please specify______<br>[12] No relation/ House mate
92	What is their gender (sex)?	[1] Male

		[2] Female<br>[3] Other<br>[4] Prefer not to answer
93	What is their date of birth?	dd/mm/yyyy
94	If date of birth is unknown, what age-group are they in?	[1] 0-4<br>[2] 5-9<br>[3] 10-14<br>[4] 15-19<br>[5] 20-24<br>[6] 25-29<br>[7] 30-34<br>[8] 35-39<br>[9] 40-44<br>[10] 45-49<br>[11] 50-54<br>[12] 55-59<br>[13] 60-64<br>[14] 65-69<br>[5] 70-74<br>[6] 75-79<br>[7] 80-84<br>[8] 85-89<br>[9] 90 and above
95	What is their working status?	[1] Employed, Full-time<br>[2] Employed, Part-time<br>[3] Unemployed<br>[4] Other<br>[5] Retired<br>[6] Child < 5 attending nursery school, pre-school or child minder<br>[7] Child > 5 attending formal school
96	Have they ever tested positive for a COVID-19 test?	[0] No<br>[1] Yes<br>[2] Do not know
97	If yes - date/month of the test?	

	**Household Member 2:**<br>*In RedCap, this section must be repeated to allow for up to 10 household members to be inserted.*	
98	Relationship to learner:	[1] Spouse/Partner<br>[2] Child/Stepchild<br>[3] Mother/Mother-in-law<br>[4] Father/Father-in-law<br>[5] Brother/Stepbrother<br>[6] Sister/Stepsister<br>[7]Grand parent<br>[9] Aunt<br>[10] Uncle<br>[11] Other relative, please specify______<br>[12] No relation/ House mate
99	What is their gender (sex)?	[1] Male<br>[2] Female<br>[3] Other<br>[4] Prefer not to answer
100	What is their date of birth?	dd/mm/yyyy
101	If date of birth is unknown, what age-group are they in?	[1] 0-4<br>[2] 5-9<br>[3] 10-14<br>[4] 15-19<br>[5] 20-24<br>[6] 25-29<br>[7] 30-34<br>[8] 35-39<br>[9] 40-44<br>[10] 45-49<br>[11] 50-54<br>[12] 55-59<br>[13] 60-64<br>[14] 65-69<br>[5] 70-74<br>[6] 75-79<br>[7] 80-84<br>[8] 85-89<br>[9] 90 and above
102	What is their working status?	[1] Employed, Full-time

		[2] Employed, Part-time<br>[3] Unemployed<br>[4] Other<br>[5] Retired<br>[6] Child < 5 attending nursery school, pre-school or child minder<br>[7] Child > 5 attending formal school
103	Have they ever tested positive for a COVID-19 test?	[0] No<br>[1] Yes<br>[2] Do not know
104	If yes - date/month of the test?	
	**Household Member 3:**<br>*In RedCap, this section must be repeated to allow for up to 10 household members to be inserted.*	
105	Relationship to learner:	[1] Spouse/Partner<br>[2] Child/Stepchild<br>[3] Mother/Mother-in-law<br>[4] Father/Father-in-law<br>[5] Brother/Stepbrother<br>[6] Sister/Stepsister<br>[7]Grand parent<br>[9] Aunt<br>[10] Uncle<br>[11] Other relative, please specify______<br>[12] No relation/ House mate
106	What is their gender?	[1] Male<br>[2] Female<br>[3] Other<br>[4] Prefer not to answer
107	What is their date of birth?	dd/mm/yyyy
108	If date of birth is unknown, what age-group	[1] 0-4

	are they in?	[2] 5-9<br>[3] 10-14<br>[4] 15-19<br>[5] 20-24<br>[6] 25-29<br>[7] 30-34<br>[8] 35-39<br>[9] 40-44<br>[10] 45-49<br>[11] 50-54<br>[12] 55-59<br>[13] 60-64<br>[14] 65-69<br>[5] 70-74<br>[6] 75-79<br>[7] 80-84<br>[8] 85-89<br>[9] 90 and above
109	What is their working status?	[1] Employed, Full-time<br>[2] Employed, Part-time<br>[3] Unemployed<br>[4] Other<br>[5] Retired<br>[6] Child < 5 attending nursery school, pre-school or child minder<br>[7] Child > 5 attending formal school
110	Have they ever tested positive for a COVID-19 test?	[1] Yes<br>[2] No<br>[3] Do not know
111	If yes - date/month of the test?	
	**Household Member 4:**<br>*In RedCap, this section must be repeated to allow for up to 10 household members to be inserted.*	
112	Relationship to learner:	[1] Spouse/Partner<br>[2] Child/Stepchild<br>[3] Mother/Mother-in-law

		[4] Father/Father-in-law<br>[5] Brother/Stepbrother<br>[6] Sister/Stepsister<br>[7]Grand parent<br>[9] Aunt<br>[10] Uncle<br>[11] Other relative, please specify______<br>[12] No relation/ House mate
113	What is their gender?	[1] Male<br>[2] Female<br>[3] Other<br>[4] Prefer not to answer
114	What is their date of birth?	dd/mm/yyyy
115	If date of birth is unknown, what age-group are they in?	[1] 0-4<br>[2] 5-9<br>[3] 10-14<br>[4] 15-19<br>[5] 20-24<br>[6] 25-29<br>[7] 30-34<br>[8] 35-39<br>[9] 40-44<br>[10] 45-49<br>[11] 50-54<br>[12] 55-59<br>[13] 60-64<br>[14] 65-69<br>[5] 70-74<br>[6] 75-79<br>[7] 80-84<br>[8] 85-89<br>[9] 90 and above
116	What is their working status?	[1] Employed, Full-time<br>[2] Employed, Part-time<br>[3] Unemployed<br>[4] Other<br>[5] Retired

		[6] Child < 5 attending nursery school, pre-school or child minder<br>[7] Child > 5 attending formal school
117	Have they ever tested positive for a COVID-19 test?	[0] No<br>[1] Yes<br>[2] Do not know
118	If yes - date/month of the test?	
	**Household Member 5:**<br>*In RedCap, this section must be repeated to allow for up to 10 household members to be inserted.*	
119	Relationship to learner:	[1] Spouse/Partner<br>[2] Child/Stepchild<br>[3] Mother/Mother-in-law<br>[4] Father/Father-in-law<br>[5] Brother/Stepbrother<br>[6] Sister/Stepsister<br>[7]Grand parent<br>[9] Aunt<br>[10] Uncle<br>[11] Other relative, please specify______<br>[12] No relation/ House mate
120	What is their gender?	[1] Male<br>[2] Female<br>[3] Other<br>[4] Prefer not to answer
121	What is their date of birth?	dd/mm/yyyy
122	If date of birth is unknown, what age-group are they in?	[1] 0-4<br>[2] 5-9<br>[3] 10-14<br>[4] 15-19<br>[5] 20-24<br>[6] 25-29<br>[7] 30-34<br>[8] 35-39<br>[9] 40-44

		[10] 45-49<br>[11] 50-54<br>[12] 55-59<br>[13] 60-64<br>[14] 65-69<br>[5] 70-74<br>[6] 75-79<br>[7] 80-84<br>[8] 85-89<br>[9] 90 and above
123	What is their working status?	[1] Employed, Full-time<br>[2] Employed, Part-time<br>[3] Unemployed<br>[4] Other<br>[5] Retired<br>[6] Child < 5 attending nursery school, pre-school or child minder<br>[7] Child > 5 attending formal school
124	Have they ever tested positive for a COVID-19 test?	[0] No<br>[1] Yes<br>[2] Do not know
125	If yes - date/month of the test?	
	**Household Member 6:**<br>*In RedCap, this section must be repeated to allow for up to 10 household members to be inserted.*	
126	Relationship to learner:	[1] Spouse/Partner<br>[2] Child/Stepchild<br>[3] Mother/Mother-in-law<br>[4] Father/Father-in-law<br>[5] Brother/Stepbrother<br>[6] Sister/Stepsister<br>[7]Grand parent<br>[9] Aunt

		[10] Uncle<br>[11] Other relative, please specify______<br>[12] No relation/ House mate
127	What is their gender?	[1] Male<br>[2] Female<br>[3] Other<br>[4] Prefer not to answer
128	What is their date of birth?	dd/mm/yyyy
129	If date of birth is unknown, what age-group are they in?	[1] 0-4<br>[2] 5-9<br>[3] 10-14<br>[4] 15-19<br>[5] 20-24<br>[6] 25-29<br>[7] 30-34<br>[8] 35-39<br>[9] 40-44<br>[10] 45-49<br>[11] 50-54<br>[12] 55-59<br>[13] 60-64<br>[14] 65-69<br>[5] 70-74<br>[6] 75-79<br>[7] 80-84<br>[8] 85-89<br>[9] 90 and above
130	What is their working status?	[1] Employed, Full-time<br>[2] Employed, Part-time<br>[3] Unemployed<br>[4] Other<br>[5] Retired<br>[6] Child < 5 attending nursery school, pre-school or child minder<br>[7] Child > 5 attending formal school

131	Have they ever tested positive for a COVID-19 test?	[0] No<br><br>[1] Yes<br><br>[2] Do not know
132	If yes - date/month of the test?	
	**Household Member 7:**<br>*In RedCap, this section must be repeated to allow for up to 10 household members to be inserted.*	
133	Relationship to learner:	[1] Spouse/Partner<br>[2] Child/Stepchild<br>[3] Mother/Mother-in-law<br>[4] Father/Father-in-law<br>[5] Brother/Stepbrother<br>[6] Sister/Stepsister<br>[7]Grand parent<br>[9] Aunt<br>[10] Uncle<br>[11] Other relative, please specify______<br>[12] No relation/ House mate
134	What is their gender?	[1] Male<br>[2] Female<br>[3] Other<br>[4] Prefer not to answer
135	What is their date of birth?	dd/mm/yyyy
136	If date of birth is unknown, what age-group are they in?	[1] 0-4<br>[2] 5-9<br>[3] 10-14<br>[4] 15-19<br>[5] 20-24<br>[6] 25-29<br>[7] 30-34<br>[8] 35-39<br>[9] 40-44<br>[10] 45-49<br>[11] 50-54<br>[12] 55-59<br>[13] 60-64<br>[14] 65-69<br>[5] 70-74<br>[6] 75-79

		[7] 80-84<br>[8] 85-89<br>[9] 90 and above
137	What is their working status?	[1] Employed, Full-time<br>[2] Employed, Part-time<br>[3] Unemployed<br>[4] Other<br>[5] Retired<br>[6] Child < 5 attending nursery school, pre-school or child minder<br>[7] Child > 5 attending formal school
138	Have they ever tested positive for a COVID-19 test?	[0] No<br>[1] Yes<br>[2] Do not know
139	If yes - date/month of the test?	
	**Household Member 8:**<br>*In RedCap, this section must be repeated to allow for up to 10 household members to be inserted.*	
140	Relationship to learner:	[1] Spouse/Partner<br>[2] Child/Stepchild<br>[3] Mother/Mother-in-law<br>[4] Father/Father-in-law<br>[5] Brother/Stepbrother<br>[6] Sister/Stepsister<br>[7]Grand parent<br>[9] Aunt<br>[10] Uncle<br>[11] Other relative, please specify______<br>[12] No relation/ House mate
141	What is their gender?	[1] Male<br>[2] Female

		[3] Other<br>[4] Prefer not to answer
142	What is their date of birth?	dd/mm/yyyy
143	If date of birth is unknown, what age-group are they in?	[1] 0-4<br>[2] 5-9<br>[3] 10-14<br>[4] 15-19<br>[5] 20-24<br>[6] 25-29<br>[7] 30-34<br>[8] 35-39<br>[9] 40-44<br>[10] 45-49<br>[11] 50-54<br>[12] 55-59<br>[13] 60-64<br>[14] 65-69<br>[5] 70-74<br>[6] 75-79<br>[7] 80-84<br>[8] 85-89<br>[9] 90 and above
144	What is their working status?	[1] Employed, Full-time<br>[2] Employed, Part-time<br>[3] Unemployed<br>[4] Other<br>[5] Retired<br>[6] Child < 5 attending nursery school, pre-school or child minder<br>[7] Child > 5 attending formal school
145	Have they ever tested positive for a COVID-19 test?	[0] No<br>[1] Yes<br>[2] Do not know
146	If yes - date/month of the test?	
	**Household Member 9:**	

	In RedCap, this section must be repeated to allow for up to 10 household members to be inserted.	
147	Relationship to learner:	[1] Spouse/Partner<br>[2] Child/Stepchild<br>[3] Mother/Mother-in-law<br>[4] Father/Father-in-law<br>[5] Brother/Stepbrother<br>[6] Sister/Stepsister<br>[7]Grand parent<br>[9] Aunt<br>[10] Uncle<br>[11] Other relative, please specify______<br>[12] No relation/ House mate
148	What is their gender?	[1] Male<br>[2] Female<br>[3] Other<br>[4] Prefer not to answer
149	What is their date of birth?	dd/mm/yyyy
150	If date of birth is unknown, what age-group are they in?	[1] 0-4<br>[2] 5-9<br>[3] 10-14<br>[4] 15-19<br>[5] 20-24<br>[6] 25-29<br>[7] 30-34<br>[8] 35-39<br>[9] 40-44<br>[10] 45-49<br>[11] 50-54<br>[12] 55-59<br>[13] 60-64<br>[14] 65-69<br>[5] 70-74<br>[6] 75-79<br>[7] 80-84<br>[8] 85-89<br>[9] 90 and above
151	What is their working status?	[1] Employed, Full-time

		[2] Employed, Part-time<br>[3] Unemployed<br>[4] Other<br>[5] Retired<br>[6] Child < 5 attending nursery school, pre-school or child minder<br>[7] Child > 5 attending formal school
152	Have they ever tested positive for a COVID-19 test?	[0] No<br>[1] Yes<br>[2] Do not know
153	If yes - date/month of the test?	
	**Household Member 10:**<br>*In RedCap, this section must be repeated to allow for up to 10 household members to be inserted.*	
154	Relationship to learner:	[1] Spouse/Partner<br>[2] Child/Stepchild<br>[3] Mother/Mother-in-law<br>[4] Father/Father-in-law<br>[5] Brother/Stepbrother<br>[6] Sister/Stepsister<br>[7]Grand parent<br>[9] Aunt<br>[10] Uncle<br>[11] Other relative, please specify______<br>[12] No relation/ House mate
155	What is their gender?	[1] Male<br>[2] Female<br>[3] Other<br>[4] Prefer not to answer
156	What is their date of birth?	dd/mm/yyyy
157	If date of birth is unknown, what age-group	[1] 0-4

	are they in?	[2] 5-9<br>[3] 10-14<br>[4] 15-19<br>[5] 20-24<br>[6] 25-29<br>[7] 30-34<br>[8] 35-39<br>[9] 40-44<br>[10] 45-49<br>[11] 50-54<br>[12] 55-59<br>[13] 60-64<br>[14] 65-69<br>[5] 70-74<br>[6] 75-79<br>[7] 80-84<br>[8] 85-89<br>[9] 90 and above
158	What is their working status?	[1] Employed, Full-time<br>[2] Employed, Part-time<br>[3] Unemployed<br>[4] Other<br>[5] Retired<br>[6] Child < 5 attending nursery school, pre-school or child minder<br>[7] Child > 5 attending formal school
159	Have they ever tested positive for a COVID-19 test?	[0] No<br>[1] Yes<br>[1] Do not know
160	If yes - date/month of the test?	

**Learner: Specimen collection**<br>*This section is to be completed by study staff collecting the specimens from the learner*		
161	Date of specimen collection?	dd/mm/yyyy

162	Was blood collected for Rapid COVID-19 antibody POC test?	[0] No<br>[1] Yes<br>[99] N/A
163	If yes, what was the name of the test?	[1] Orient gene<br>[2] Other<br>*If other, please specify*
164	What was the result?<br>*(more than one answer allowed)*	[1] Indeterminate<br>[2] IgG positive<br>[3] IgM positive<br>[99] N/A
165	Was blood collected for DBS?	[0] No<br>[1] Yes<br>[99] N/A
166	Was saliva collected for future testing?	[0] No<br>[1] Yes<br>[99] N/A

**Learner: Mental health**		
167	My child feels empty	[1] Never<br>[2] Sometimes<br>[3] Often<br>[4] Always
168	My child feels afraid of being alone at home	[1] Never<br>[2] Sometimes<br>[3] Often<br>[4] Always
169	My child worries when he/she think he/she has done poorly at something	[1] Never<br>[2] Sometimes<br>[3] Often<br>[4] Always
170	Nothing is much fun for my child anymore	[1] Never<br>[2] Sometimes<br>[3] Often<br>[4] Always
171	My child worries that something awful will happen to someone in the family	[1] Never<br>[2] Sometimes<br>[3] Often<br>[4] Always
172	My child is afraid of being in crowded places like shopping centers, the movies, buses, playgrounds	[1] Never<br>[2] Sometimes<br>[3] Often<br>[4] Always
173	My child worries what other people think of him/her	[1] Never<br>[2] Sometimes<br>[3] Often

		[4] Always
174	My child has trouble sleeping	[1] Never<br>[2] Sometimes<br>[3] Often<br>[4] Always
175	My child feels scared to sleep on his/her own	[1] Never<br>[2] Sometimes<br>[3] Often<br>[4] Always
176	My child has problems with his/her appetite	[1] Never<br>[2] Sometimes<br>[3] Often<br>[4] Always
177	My child feels dizzy or faints when there is no reason for this	[1] Never<br>[2] Sometimes<br>[3] Often<br>[4] Always
178	My child has to do some things over and over again (like washing hands, cleaning or putting things in a certain order)	[1] Never<br>[2] Sometimes<br>[3] Often<br>[4] Always
179	My child has no energy for things	[1] Never<br>[2] Sometimes<br>[3] Often<br>[4] Always
180	My child cannot think clearly	[1] Never<br>[2] Sometimes<br>[3] Often<br>[4] Always
181	My child suddenly starts to tremble or shake when there is no reason for this	[1] Never<br>[2] Sometimes<br>[3] Often<br>[4] Always
182	My child feels worthless	[1] Never<br>[2] Sometimes<br>[3] Often<br>[4] Always
183	My child has to think of special thoughts (like number or words) to stop bad things from happening	[1] Never<br>[2] Sometimes<br>[3] Often<br>[4] Always
184	My child thinks about death	[1] Never<br>[2] Sometimes<br>[3] Often<br>[4] Always
185	My child feels like he/she does not want to move	[1] Never<br>[2] Sometimes

		[3] Often<br>[4] Always
186	My child worries that he/she will suddenly get a scared feeling when there is nothing to be afraid of	[1] Never<br>[2] Sometimes<br>[3] Often<br>[4] Always
187	My child is tired alot	[1] Never<br>[2] Sometimes<br>[3] Often<br>[4] Always
188	My child feels like he/she will make a fool of him/her self in front of people	[1] Never<br>[2] Sometimes<br>[3] Often<br>[4] Always
189	My child has to do some things in just the right way to stop bad things from happening	[1] Never<br>[2] Sometimes<br>[3] Often<br>[4] Always
190	My child feels restless	[1] Never<br>[2] Sometimes<br>[3] Often<br>[4] Always
191	My child worries that something bad will happen to him or her	[1] Never<br>[2] Sometimes<br>[3] Often<br>[4] Always

FOLLOW-UP SURVEY:
eCRF FOR PARENT/ GUARDIAN OF LEARNER IN GRADE 1-7

	Instructions:<br>1. *All instructions are in italics.*<br>2. *In this study, parent also refers to legal guardian.*	
1	Visit Code	
2	Research staff ID	
3	Does the child have an SA ID or passport?	
4	What is the child's SA ID or passport number?	
5	Study unique identifier (Child)<br>*The RA will obtain the participant's unique identifier from the link log that was completed in the cross-sectional study.*	
6	Re-enter study unique identifier<br>*RA to complete.*	
	Does the parent have an SA ID or passport?	
7	What is your SA ID or passport number?	
8	Study unique identifier (Parent)<br>*The RA will obtain the participant's unique identifier from the link log that was completed in the cross-sectional study.*	
9	Re-enter study unique identifier<br>*RA to complete.*	
10	Today's date	dd/mm/yyyy
11	What is the name of the school your child attends?	
12	What grade is your child in?	[1] Grade 1<br>[2] Grade 2<br>[3] Grade 3<br>[4] Grade 4<br>[5] Grade 5<br>[6] Grade 6<br>[7] Grade 7
13	What is the name of your child's class?<br>*Class ID to be codified.*	

**Parent/ guardian: Acute COVID-19**			
14	Are you currently feeling sick?		[0] No<br>[1] Yes
15	*Do you have any of the following symptoms now? If yes, indicate which signs/symptoms are currently present, and duration and severity*		
Cough	[0] No	Approximate	Seriousness today:

	[1] Yes	duration (in days)	[1] I could do everything that I usually do<br>[2] I could not do some of what I usually do<br>[3] I could not do some of what I usually do most of what I usually do
Sore Throat	[0] No<br>[1] Yes	Approximate duration (in days	Seriousness today:<br>[1] I could do everything that I usually do<br>[2] I could not do some of what I usually do<br>[3] I could not do some of what I usually do most of what I usually do
Fever	[0] No<br>[1] Yes	Approximate duration (in days	Seriousness today:<br>[1] I could do everything that I usually do<br>[2] I could not do some of what I usually do<br>[3] I could not do some of what I usually do most of what I usually do
Body ache	[0] No<br>[1] Yes	Approximate duration (in days *)*	seriousness today:<br>[1] I could do everything that I usually do<br>[2] I could not do some of what I usually do<br>[3] I could not do some of what I usually do most of what I usually do
Diarrhe a	[0] No<br>[1] Yes	Approximate duration (in days	Seriousness today:<br>[1] I could do everything that I usually do<br>[2] I could not do some of what I usually do

			[3] I could not do some of what I usually do most of what I usually do
Nause a/vomit ing	[0] No [1] Yes	Approximate duration (in days	Seriousness today: [1] I could do everything that I usually do [2] I could not do some of what I usually do [3] I could not do some of what I usually do most of what I usually do
Painful muscle and joints	[0] No [1] Yes	Approximate duration (in days	Seriousness today: [1] I could do everything that I usually do [2] I could not do some of what I usually do [3] I could not do some of what I usually do most of what I usually do
Loss of smell	[0] No [1] Yes	Approximate duration (in days	Seriousness today: [1] I could do everything that I usually do [2] I could not do some of what I usually do [3] I could not do some of what I usually do most of what I usually do
Loss of taste	[0] No [1] Yes	Approximate duration (in days	Seriousness today: [1] I could do everything that I usually do [2] I could not do some of what I usually do [3] I could not do some of what I usually do most of what I usually do
Tiredness and fatigue	[0] No [1] Yes	Approximate duration (in days	Seriousness today: [1] I could do everything that I usually do

			[2] I could not do some of what I usually do<br>[3] I could not do some of what I usually do most of what I usually do
Chills	[0] No<br>[1] Yes	Approximate duration (in days	Seriousness today:<br>[1] I could do everything that I usually do<br>[2] I could not do some of what I usually do<br>[3] I could not do some of what I usually do most of what I usually do
Headache	[0] No<br>[1] Yes	Approximate duration (in days	Seriousness today:<br>[1] I could do everything that I usually do<br>[2] I could not do some of what I usually do<br>[3] I could not do some of what I usually do most of what I usually do
Irritability or confusion	[0] No<br>[1] Yes	Approximate duration (in days	Seriousness today:<br>[1] I could do everything that I usually do<br>[2] I could not do some of what I usually do<br>[3] I could not do some of what I usually do most of what I usually do
General weakness	[0] No<br>[1] Yes	Approximate duration (in days	Seriousness today:<br>[1] I could do everything that I usually do<br>[2] I could not do some of what I usually do<br>[3] I could not do some of what I usually do most of what I usually do
Skin rash	[0] No	Approximate	Seriousness today:

	[1] Yes	duration (in days	[1] I could do everything that I usually do<br>[2] I could not do some of what I usually do<br>[3] I could not do some of what I usually do most of what I usually do

16	When did your symptoms first present?	dd/mm/yyyy
17	In the last month, have you had close contact with any of the following people:<br>*Note: Close contact means: face-to-face contact without a mask (≤1 meter) OR been in a closed space with a confirmed case for at least 15 minutes with or without a mask OR lived in the same household OR provided direct care without the recommended PPE:*	A suspected COVID-19 patient …<br>[0] No [1] Yes<br>A confirmed COVID-19 patient<br>[0] No [1] Yes…<br>Someone with the “flu” or “cold”<br>[0] No [1] Yes…
18	If yes for any of the above, what setting was the contact<br>*(Please select the answer that applies)*	[1] Healthcare setting<br>[2] Family setting<br>[3] School setting<br>[4] Public transport setting<br>[5] Other<br>*If other, please specify.*
19	Did you quarantine after the contact?	[0] No<br>[1] Yes
20	If yes, for how long did you quarantine?	days
21	If no, how many contacts have you had since that time?	
22	Have you been diagnosed with COVID-19 before?	[0] No<br>[1] Yes
23	If yes, how many times?	
24	Which was their most recent time? Give an approximate date of diagnosis	dd/mm/yyyy
25	Over the past 2 years have you felt sick or more tired, or had headaches or lost his/her taste or had COVID-19-like symptoms (signs) for 28-days or longer?	[0] No<br>[1] Yes

**Parent/ Guardian: Long COVID**<br>*(Only answer question if answer to question 25 was yes)*	
26	Tell us which signs/symptoms were present for more than 28-days.

Fatigue<br>[0] No<br>[1] Yes	
Stuffy/runny nose<br>[0] No<br>[1] Yes	Chest tightness<br>[0] No<br>[1] Yes
Chest pain<br>[0] No<br>[1] Yes	Cough<br>[0] No<br>[1] Yes
Wheezing<br>[0] No<br>[1] Yes	Sore throat<br>[0] No<br>[1] Yes
Muscle ache<br>[0] No<br>[1] Yes	Joint pain/swelling<br>[0] No<br>[1] Yes
Headache<br>[0] No<br>[1] Yes	Dizziness<br>[0] No<br>[1] Yes
Altered sense of taste (change in taste)<br>[0] No<br>[1] Yes	Altered sense of smell<br>[0] No<br>[1] Yes
Difficulty concentrating (focusing)<br>[0] No<br>[1] Yes	Sleep disorders<br>[0] No<br>[1] Yes
Mood alterations<br>[0] No<br>[1] Yes	Cognitive dysfunction<br>(loss of memory or difficulty processing information or paying attention)<br>[0] No<br>[1] Yes
Sensorimotor symptoms (tingling in the toes / feet / legs / fingers / hands or twitching of muscles)<br>[0] No<br>[1] Yes	Increased need for sleep<br>[0] No<br>[1] Yes
Weight loss<br>[0] No<br>[1] Yes	Diarrhea<br>[0] No<br>[1] Yes
Stomach pain<br>[0] No<br>[1] Yes	Poor appetite<br>[0] No<br>[1] Yes
Constipation<br>[0] No<br>[1] Yes	Skin rash<br>[0] No<br>[1] Yes
Tachycardia	Fever

(fast heart rate)<br>[0] No<br>[1] Yes		[0] No<br>[1] Yes
Other<br>*If other, please specify.*<br>*Please provide space to specify*		
36	If, you have selected at least one symptom (sign) above. Please select what applies:	[1] A doctor had to be seen because of this<br>[2] You had to stay away from work<br>*(If applicable answer question 37)*<br>[3] You had to be treated with medication<br>*(If applicable answer question 38)*<br>[4] You had to be hospitalized<br>*(If applicable answer question 39)*<br>[5] You have not done anything<br>[6] Other<br>*If other, please specify.*<br>*Please provide space to specify. (multiple answers possible)*
37	How many days did you have to miss?	_____*(Please enter number in days)*
38	How many days did you have to be treated with medication?	_____*(Please enter number in days)*
39	How many days did you have to spend in the hospital?	_____*(Please enter number in days)*
**Parent/ Guardian: COVID-19 vaccination and vaccination history**		
40	Have you been vaccinated against COVID-19?	[0] No<br>[1] Yes
41	If you have not been vaccinated, we would be interested to know why?	[1] It is a choice and I choose not too<br>[2] No time<br>[3] In general, my family is against all vaccinations<br>[4] No expected benefit (vaccination does not work at all or not enough)<br>[5] I do not trust the vaccine manufacturing companies<br>[6] I do not trust the government's ability to roll out a safe vaccine.<br>[7] I want to wait until there is more knowledge<br>[8]I fear the side effects, safety and effectiveness of vaccinations

		[9] Due to my religious or cultural beliefs<br>[10] I am afraid of needles<br>[11] I had COVID-19, so I do not consider the vaccination necessary<br>[14] Due to my medical condition<br>[15] Other<br>If other, please specify<br>*Please provide space to specify.*<br><br>*More than 1 answer allowed.*
42	If yes, when did the learner receive their first dose? Give an approximate date	dd/mm/yyyy
43	Which vaccine did you receive?<br>(*Please select the correct answer*)	see vaccine table below
44	Date of last dose?	dd/mm/yyyy
45	Do you have any of the following complications?<br>*(Please select the correct answer)*	

	J&J	0	1	2	
	AstraZeneca	0	1	2	3
	CoronaVac	0	1	2	3
	Moderna	0	1	2	3
	Pfizer	0	1	2	3
	Other (please specify)	0	1	2	3

HIV<br>[0] No<br>[1] Yes	Current TB<br>[0] No<br>[1] Yes	Chronic Kidney Disease<br>[0] No<br>[1] Yes	Chronic Liver Disease<br>[0] No<br>[1] Yes
Neurological/neuromuscular disease<br>[0] No<br>[1] Yes	Diabetes Mellitus (high blood sugar)<br>[0] No<br>[1] Yes	Heart Disease<br>[0] No<br>[1] Yes	Cancer<br>[0] No<br>[1] Yes
Prior TB infection<br>[0] No<br>[1] Yes	Hypertension (high blood pressure)<br>[0] No<br>[1] Yes	Asthma (Difficulty breathing)<br>[0] No<br>[1] Yes	Chronic Lung Disease<br>[0] No<br>[1] Yes
Rheumatological disease	Obesity/overweight	Autoimmune disease (not HIV. A disease	Other 1: Details<br>Other 2: Details

(disease of the joints and muscles)<br>[0] No<br>[1] Yes	[0] No<br>[1] Yes	whereby our immune system starts attacking our own tissues or organs e.g., SLE)<br>[0] No<br>[1] Yes	Other 3: Details<br>Other 4: Details

46	Are you currently taking any of the following medications NOW:
Steroids<br>(e.g., Prednisone, cortisone)<br>[0] No<br>[1] Yes<br>[2] Prefer not to answer	Anti-inflammatories<br>(e.g., high dose aspirin, ibuprofen)<br>[0] No<br>[1] Yes<br>[2] Prefer not to answer
Anti-hypertensives<br>(blood pressure medication e.g., indapamide)<br>[0] No<br>[1] Yes<br>[2] Prefer not to answer	Chemotherapy<br>(cancer treatment)<br>[0] No<br>[1] Yes<br>[2] Prefer not to answer
Hormonal treatment<br>[0] No<br>[1] Yes<br>[2] Prefer not to answer	Antibiotics<br>(e.g., penicillin, amoxicillin)<br>[0] No<br>[1] Yes<br>[2] Prefer not to answer
ARV/ART<br>[0] No<br>[1] Yes<br>[2] Prefer not to answer	Bactrim prophylaxis<br>[0] No<br>[1] Yes<br>[2] Prefer not to answer
Aspirin / Warfarin / Heparin<br>[0] No<br>[1] Yes<br>[2] Prefer not to answer	TB Meds<br>[0] No<br>[1] Yes<br>[2] Prefer not to answer
Other 1: Details<br>Other 2: Details<br>Other 3: Details	

47	What non pharmaceutical measures do you currently use to prevent COVID-19?		
Masks in public places e.g., buses and taxis<br>[0] No<br>[1] Yes always<br>[2] Yes sometimes	Sanitizing<br>[0] No<br>[1] Yes always<br>[2] Yes sometimes<br>[99] N/A	Masks in the workplace<br>[0] No<br>[1] Yes always<br>[2] Yes sometimes<br>[99] N/A	Distancing- - more than 1.5 meters away in the workplace?<br>[0] No<br>[1] Yes always

[99] N/A			[2] Yes sometimes<br>[99] N/A
Avoiding social gatherings/outings<br>[0] No<br>[1] Yes always<br>[2] Yes sometimes<br>[99] N/A	Avoiding weddings<br>[0] No<br>[1] Yes always<br>[2] Yes sometimes<br>[99] N/A	Avoiding funerals<br>[0] No<br>[1] Yes always<br>[2] Yes sometimes<br>[99] N/A	

**Parent/ Guardian: Specimen collection**<br>*This section is to be completed by study staff collecting the specimens from the parent/legal guardian*		
48	Date of specimen collection?	dd/mm/yyyy
49	Was blood collected for Rapid COVID-19 antibody POC test?	[0] No<br>[1] Yes<br>[99] N/A
50	If yes, what was the name of the test?	[1] Orient gene<br>[2] Other<br>*If other, please specify*
51	What was the result?<br>*(more than one answer allowed)*	[1] Indeterminate<br>[2] IgG positive<br>[3] IgM positive<br>[99] N/A
52	Was blood collected for DBS?	[0] No<br>[1] Yes<br>[99] N/A
53	Was saliva collected for future testing?	[0] No<br>[1] Yes<br>[99] N/A
**Learner: Acute COVID-19 infection**		
54	Is the learner currently feeling sick?	[0] No<br>[1] Yes
54	*Does the learner have any of the following symptoms now? If yes, indicate which symptoms are currently present, and approximate duration and severity*	

Cough	[0] No<br>[1] Yes	Approximate duration (in days)	Seriousness today:<br>[1] I could do everything that I usually do<br>[2] I could not do some of

			what I usually do<br>[3] I could not do some of what I usually do most of what I usually do
Sore Throat	[0] No<br>[1] Yes	Approximate duration (in days)	Seriousness today:<br>[1] I could do everything that I usually do<br>[2] I could not do some of what I usually do<br>[3] I could not do some of what I usually do most of what I usually do
Fever	[0] No<br>[1] Yes	Approximate duration (in days)	Seriousness today:<br>[1] I could do everything that I usually do<br>[2] I could not do some of what I usually do<br>[3] I could not do some of what I usually do most of what I usually do
Body ache	[0] No<br>[1] Yes	Approximate duration (in days)	Seriousness today:<br>[1] I could do everything that I usually do<br>[2] I could not do some of what I usually do<br>[3] I could not do some of what I usually do most of

			what I usually do
Diarrhea	[0] No<br>[1] Yes	Approximate duration (in days)	Seriousness today:<br>[1] I could do everything that I usually do<br>[2] I could not do some of what I usually do<br>[3] I could not do some of what I usually do most of what I usually do
Nausea/vomiting	[0] No<br>[1] Yes	Approximate duration (in days)	Seriousness today:<br>[1] I could do everything that I usually do<br>[2] I could not do some of what I usually do<br>[3] I could not do some of what I usually do most of what I usually do
Painful muscles and joints	[0] No<br>[1] Yes	Approximate duration (in days)	Seriousness today:<br>[1] I could do everything that I usually do<br>[2] I could not do some of what I usually do<br>[3] I could not do some of what I usually do most of what I usually do
Loss of smell	[0] No<br>[1] Yes	Approximate duration (in days)	Seriousness today:<br>[1] I could do everything

			that I usually do [2] I could not do some of what I usually do [3] I could not do some of what I usually do most of what I usually do
Loss of taste	[0] No [1] Yes	Approximate duration (in days)	Seriousness today: [1] I could do everything that I usually do [2] I could not do some of what I usually do [3] I could not do some of what I usually do most of what I usually do
Tiredness and fatigue	[0] No [1] Yes	Approximate duration (in days)	Seriousness today: [1] I could do everything that I usually do [2] I could not do some of what I usually do [3] I could not do some of what I usually do most of what I usually do
Chills	[0] No [1] Yes	Approximate duration (in days)	Seriousness today: [1] I could do everything that I usually do [2] I could not do some of what I usually

			do<br>[3] I could not do some of what I usually do most of what I usually do
Headache	[0] No<br>[1] Yes	Approximate duration (in days)	Seriousness today:<br>[1] I could do everything that I usually do<br>[2] I could not do some of what I usually do<br>[3] I could not do some of what I usually do most of what I usually do
Irritability or confusion	[0] No<br>[1] Yes	Approximate duration (in days)	Seriousness today:<br>[1] I could do everything that I usually do<br>[2] I could not do some of what I usually do<br>[3] I could not do some of what I usually do most of what I usually do
General weakness	[0] No<br>[1] Yes	Approximate duration (in days)	Seriousness today:<br>[1] I could do everything that I usually do<br>[2] I could not do some of what I usually do<br>[3] I could not do some of what I usually do most of what I usually do

Skin rash	[0] No<br>[1] Yes	Approximate duration (in days)	Seriousness today:<br>[1] I could do everything that I usually do<br>[2] I could not do some of what I usually do<br>[3] I could not do some of what I usually do most of what I usually do

56	When did the learner's symptoms first present?	dd/mm/yyyy
57	**In the last month, has the learner had close contact with someone with suspected COVID-19, confirmed COVID-19, flu or a cold?**<br>*Note: Close contact means the learner ate with or played with this person. They were close together for at least 15 minutes*	A suspected COVID-19 patient<br>[0] No [1] Yes<br>A confirmed COVID-19 patient<br>[0] No [1] Yes<br>Someone with the "flu" or "cold"<br>[0] No [1] Yes
58	If yes for any of the above, what setting was the contact:<br>*(Please select the answer that applies)*	[1] Healthcare setting<br>[2] Family setting<br>[3] School setting<br>[4] Public transport setting<br>[5] Other<br>*If other, please specify.*
59	Did the learner quarantine after the contact?	[0] No<br>[1] Yes
60	If yes, for how long did the learner quarantine?	____________days
61	If no, how many contacts did the learner have since that time?	
62	Has the learner been diagnosed with COVID-19 before?	[0] No<br>[1] Yes
63	If yes, how many times?	
64	Which was their most recent time? Give an approximate date of diagnosis	dd/mm/yyyy
65	Over the past 2 years has the learner felt sick or more tired, or had headaches or lost his/her taste or had COVID-19-like symptoms (signs) for 28-days or longer?	[0] No<br>[1] Yes

**Learner: Long COVID**<br>*(Only answer question if answer to question 65 was yes)*		
66	Tell us which signs/symptoms were present for more than 28-days.	
No symptoms<br>[0] No<br>[1] Yes		Fatigue<br>[0] No<br>[1] Yes
Stuffy/runny nose<br>[0] No<br>[1] Yes		Chest tightness<br>[0] No<br>[1] Yes
Chest pain<br>[0] No<br>[1] Yes		Cough<br>[0] No<br>[1] Yes
Wheezing<br>[0] No<br>[1] Yes		Sore throat<br>[0] No<br>[1] Yes
Muscle ache<br>[0] No<br>[1] Yes		Joint pain/swelling<br>[0] No<br>[1] Yes
Headache<br>[0] No<br>[1] Yes		Dizziness<br>[0] No<br>[1] Yes
Altered sense of taste (change in taste)<br>[0] No<br>[1] Yes		Altered sense of smell<br>[0] No<br>[1] Yes
Difficulty concentrating (focusing)<br>[0] No<br>[1] Yes		Sleep disorders<br>[0] No<br>[1] Yes
Mood alterations<br>[0] No<br>[1] Yes		Cognitive dysfunction (loss of memory or difficulty processing information or paying attention)<br>[0] No<br>[1] Yes
Sensorimotor symptoms (tingling in the toes / feet / legs / fingers / hands or twitching of muscles)<br>[0] No<br>[1] Yes		Increased need for sleep<br>[0] No<br>[1] Yes
Weight loss<br>[0] No<br>[1] Yes		Diarrhea<br>[0] No<br>[1] Yes
Stomach pain<br>[0] No<br>[1] Yes		Poor appetite<br>[0] No<br>[1] Yes
Constipation		Skin rash

[0] No<br>[1] Yes	[0] No<br>[1] Yes
Tachycardia<br>(fast heart rate)<br>[0] No<br>[1] Yes	Fever<br>[0] No<br>[1] Yes
Other<br>*If other, please specify.*<br>*Please provide space to specify*	

67	If, you have selected at least one symptom (sign) above. Please select what applies:	[1] A doctor had to be consulted because of this<br>[2] He/she had to stay away from school<br>*(If applicable answer question 67)*<br>[3] He/she had to be treated with medication<br>*(If applicable answer question 68)*<br>[4] He/she had to be hospitalized<br>*(If applicable answer question 69)*<br>[5] He/she has not done anything<br>[6] Other<br>*If other, please specify.*<br>*Please provide space to specify.*<br>*(multiple answers possible)*
68	How many days did the learner have to miss school?	____*(Please enter number in days)*
69	How many days did the learner have to be treated with medication?	____*(Please enter number in days)*
70	How many days did the learner have to spend in the hospital?	____*(Please enter number in days)*

**Learner (≥ 12 years of age): COVID-19 vaccination and vaccine hesitancy**<br>*These questions are applicable for learners that are eligible for the COVID-19 vaccination (≥ 12 years of age)*		
71	Has the learner been vaccinated against COVID-19?	[0] No<br>[1] Yes

72	If the learner has not been vaccinated, we would be interested to know why not?	1] It is a choice and the learner or learner's parent/legal guardian choose not too<br>[2] No time<br>[3] In general, the learner's family is against all vaccinations<br>[4] No expected benefit (vaccination does not work at all or not sufficiently)<br>[5] The learner or learner's parent/legal guardian does not trust the vaccine manufacturing companies<br>[6] The learner or learner's parent/legal guardian does not trust the government's ability to roll out a safe vaccine.<br>[7] The learner or learner's parent/legal guardian wants to wait until there is more knowledge<br>[8] The learner or learner's parent/legal guardian fear the side effects, safety and effectiveness of vaccinations<br>[9] Due to the learner or learner's parnet/legal guardian religious or cultural beliefs<br>[10] The learner is afraid of needles<br>[11] The learner had COVID-19, so I do not consider the vaccination necessary<br>[13] The learner's parent/legal guardian just won't let them take it.<br>[14] Due to the learner's medical condition<br>[15] Other<br>If other, please specify<br>*Please provide space to specify.*<br><br>*More than 1 answer allowed.*
73	If yes, when did the learner receive their first dose? Give an approximate date	dd/mm/yyyy
74	Which vaccine did the learner receive?	[1] Pfizer<br>[2] CoronaVac

		[3] Other (specify)<br>[4] Do not know
75	How many doses did the learner receive?<br>*Circle the correct answer*	Pfizer: 0 1 2 3<br>(CoronaVac): 0 1 2 3<br>Other (specify): 0
76	Date of last (most recent) dose?	dd/mm/yyyy
**Learner: Chronic illness**		
77	Does the learner have any of the following complications from the last survey?	

HIV<br>[0] No<br>[1] Yes	Current TB<br>[0] No<br>[1] Yes	Chronic Kidney Disease<br>[0] No<br>[1] Yes	Chronic Liver Disease<br>[0] No<br>[1] Yes
Neurological/neuromuscular disease<br>[0] No<br>[1] Yes	Diabetes Mellitus (high blood sugar)<br>[0] No<br>[1] Yes	Heart Disease<br>[0] No<br>[1] Yes	Cancer<br>[0] No<br>[1] Yes
Prior TB infection<br>[0] No<br>[1] Yes	Hypertension (high blood pressure)<br>[0] No<br>[1] Yes	Asthma (Difficulty breathing)<br>[0] No<br>[1] Yes	Chronic Lung Disease<br>[0] No<br>[1] Yes
Rheumatological disease (disease of the joints and muscles)<br>[0] No<br>[1] Yes	Obesity/overweight<br>[0] No<br>[1] Yes	Autoimmune disease (not HIV. A disease whereby our immune system starts attacking our own tissues or organs e.g., SLE)<br>[0] No<br>[1] Yes	Other 1: Details<br>Other 2: Details<br>Other 3: Details<br>Other 4: Details

78	Is the learner currently taking any of the following medications NOW:
Steroids<br>(e.g., Prednisone, cortisone)<br><br>[0] No<br>[1] Yes<br>[99] Prefer not to answer	Anti-inflammatories<br>(e.g., high dose aspirin, ibuprofen)<br><br>[0] No<br>[1] Yes<br>[99] Prefer not to answer
Anti-hypertensives	Chemotherapy

(blood pressure medication e.g., indapamide)<br><br>[0] No<br>[1] Yes<br>[99] Prefer not to answer	(cancer treatment)<br><br>[0] No<br>[1] Yes<br>[99] Prefer not to answer
Hormonal treatment<br><br>[0] No<br>[1] Yes<br>[99] Prefer not to answer	Antibiotics (e.g., penicillin, amoxicillin)<br><br>[0] No<br>[1] Yes<br>[99] Prefer not to answer
ARV/ART<br>[0] No<br>[1] Yes<br>[99] Prefer not to answer	Bactrim prophylaxis<br>[0] No<br>[1] Yes<br>[99] Prefer not to answer
Aspirin / Warfarin / Heparin<br>[0] No<br>[1] Yes<br>[99] Prefer not to answer	TB Meds<br>[0] No<br>[1] Yes<br>[99] Prefer not to answer
Other 1: Details<br>Other 2: Details<br>Other 3: Details	

79	What non pharmaceutical measures does the learner currently use to prevent COVID-19?		
Masks in public places e.g., buses and taxis<br>[0] No<br>[1] Yes always<br>[2] Yes – sometimes<br>[99] N/A	Sanitizing<br>[0] No<br>[1] Yes always<br>[2] Yes – sometimes<br>[99] N/A	Masks in the school setting?<br>[0] No<br>[1] Yes always<br>[2] Yes – sometimes<br>[99] N/A	Distancing- - more than 1.5 meters away in the school setting?<br>[0] No<br>[1] Yes always<br>[2] Yes – sometimes<br>[99] N/A
Avoiding social gatherings/outings<br>[0] No<br>[1] Yes always<br>[2] Yes – sometimes<br>[99] N/A	Avoiding weddings<br>[0] No<br>[1] Yes always<br>[2] Yes – sometimes<br>[99] N/A	Avoiding funerals<br>[0] No<br>[1] Yes always<br>[2] Yes – sometimes<br>[99] N/A	

Learner: Specimen collection

This section is to be completed by study staff collecting the specimens from the learner

80	Date of specimen collection?	dd/mm/yyyy
81	Was blood collected for Rapid COVID-19 antibody POC test?	[0] No<br>[1] Yes<br>[99] N/A
82	If yes, what was the name of the test?	[1] Orient gene<br>[2] Other<br>*If other, please specify*
83	What was the result?<br>*(more than one answer allowed)*	[1] Indeterminate<br>[2] IgG positive<br>[3] IgM positive<br>[99] N/A
84	Was blood collected for DBS?	[0] No<br>[1] Yes<br>[99] N/A
85	Was saliva collected for future testing?	[0] No<br>[1] Yes<br>[99] N/A

**Learner: Mental health**		
86	My child feels empty	[1] Never<br>[2] Sometimes<br>[3] Often<br>[4] Always
87	My child feels afraid of being alone at home	[1] Never<br>[2] Sometimes<br>[3] Often<br>[4] Always
88	My child worries when he/she think he/she has done poorly at something	[1] Never<br>[2] Sometimes<br>[3] Often<br>[4] Always
89	Nothing is much fun for my child anymore	[1] Never<br>[2] Sometimes<br>[3] Often<br>[4] Always
90	My child worries that something awful will happen to someone in the family	[1] Never<br>[2] Sometimes<br>[3] Often<br>[4] Always
91	My child is afraid of being in crowded places like shopping centers, the movies, buses, playgrounds	[1] Never<br>[2] Sometimes<br>[3] Often<br>[4] Always
92	My child worries what other people think of him/her	[1] Never<br>[2] Sometimes

		[3] Often<br>[4] Always
93	My child has trouble sleeping	[1] Never<br>[2] Sometimes<br>[3] Often<br>[4] Always
94	My child feels scared to sleep on his/her own	[1] Never<br>[2] Sometimes<br>[3] Often<br>[4] Always
95	My child has problems with his/her appetite	[1] Never<br>[2] Sometimes<br>[3] Often<br>[4] Always
96	My child feels dizzy or faints when there is no reason for this	[1] Never<br>[2] Sometimes<br>[3] Often<br>[4] Always
97	My child has to do some things over and over again (like washing hands, cleaning or putting things in a certain order)	[1] Never<br>[2] Sometimes<br>[3] Often<br>[4] Always
98	My child has no energy for things	[1] Never<br>[2] Sometimes<br>[3] Often<br>[4] Always
99	My child cannot think clearly	[1] Never<br>[2] Sometimes<br>[3] Often<br>[4] Always
100	My child suddenly starts to tremble or shake when there is no reason for this	[1] Never<br>[2] Sometimes<br>[3] Often<br>[4] Always
101	My child feels worthless	[1] Never<br>[2] Sometimes<br>[3] Often<br>[4] Always
102	My child has to think of special thoughts (like number or words) to stop bad things from happening	[1] Never<br>[2] Sometimes<br>[3] Often<br>[4] Always
103	My child thinks about death	[1] Never<br>[2] Sometimes<br>[3] Often<br>[4] Always
104	My child feels like he/she does not want to move	[1] Never

		[2] Sometimes<br>[3] Often<br>[4] Always
105	My child worries that he/she will suddenly get a scared feeling when there is nothing to be afraid of	[1] Never<br>[2] Sometimes<br>[3] Often<br>[4] Always
106	My child is tired alot	[1] Never<br>[2] Sometimes<br>[3] Often<br>[4] Always
107	My child feels like he/she will make a fool of him/her self in front of people	[1] Never<br>[2] Sometimes<br>[3] Often<br>[4] Always
108	My child has to do some things in just the right way to stop bad things from happening	[1] Never<br>[2] Sometimes<br>[3] Often<br>[4] Always
109	My child feels restless	[1] Never<br>[2] Sometimes<br>[3] Often<br>[4] Always
110	My child worries that something bad will happen to him or her	[1] Never<br>[2] Sometimes<br>[3] Often<br>[4] Always
