# Supplementary material for: SARS-CoV-2 Infections in a Triad of Primary School Learners (Grades 1-7), Their Parents, and Teachers in KwaZulu-Natal, South Africa: Protocol for a Cross-Sectional and Nested Case-Cohort Study
Source: JMIR Res Protoc. 2024 Dec 19;13:e52713. doi: 10.2196/52713 (PMC11695960; doi:10.2196/52713)
Supplement: Multimedia Appendix 4 [file resprot_v13i1e52713_app4.pdf]

CROSS-SECTIONAL SURVEY:
CRF FOR PARENT/PRIMARY CAREGIVER OF LEARNER IN GRADE 1-7

	<u>Instructions:</u><br>1. *All instructions are in italics.*<br>2. *In this study, parent also refers to primary caregiver.*	
1	Visit Code	
2	Research staff ID	
3	Does the child have an SA identity document or passport?	[1] SA Identity Document<br>[2] Passport
4	What is the child's SA ID or passport number?	
5	Study unique identifier (Child)<br><br>*This question will be completed when the form returned to the school.*<br>*RA must ensure that they have filled/ completed the link log with the study ID and name of participant.*	
6	Re-enter study unique identifier<br>*This question will be completed when the form is returned to the school.*<br>*RA to complete.*	
7	Today's date	dd/mm/yyyy
	**Enrollment/baseline Information of the learner**	
8	What is the name of school your child attends?	
9	What grade is your child in at school?	[1] Grade 1<br>[2] Grade 2<br>[3] Grade 3<br>[4] Grade 4<br>[5] Grade 5<br>[6] Grade 6<br>[7] Grade 7
10	What is the name of your child's class?<br>*Class ID to be CODIFIED.*	
11	What is the learner's gender (sex)?	[1] Male<br>[2] Female<br>[3]<br>Other________________<br>*If other, please specify. Please provide space to specify.*
12	What is the learner's ethnicity (race)?	[1] Black African<br>[2] Indian<br>[3] Coloured<br>[4] White<br>[5]

		Other________________<br>*If other, please specify.*<br>*Please provide space to specify.*
13	What is the learner's current address (needed for tracing purposes) and suburb?	
14	Please provide the parent/guardian's contact number?	
15	Please provide an alternate contact number	
16	How was the learner fed when they were a baby?	[1] Exclusively breastfed (explain – no other food or fluids)<br>[2] Exclusively formula fed<br>[3] Mixed fed (breastmilk and formula fed)<br>[4] Unknown
17	If the learner was exclusively breastfed, for how long?	_________months
18	If the learner was breastfed, for how long?	_________months
	**Learner: Acute COVID-19 infection**	
19	Is the learner currently feeling sick?	[0] No<br>[1] Yes
20	Does the learner have any of the following signs/symptoms now? If yes, indicate which symptoms are currently present, and indicate approximate duration and severity	

Cough	[0] No<br>[1] Yes	Approximate duration (in days)	Seriousness today:<br>[1] I could do everything that I usually do<br>[2] I could not do some of what I usually do<br>[3] I could not do some of

			what I usually do most of what I usually do
Sore Throat	[0] No<br>[1] Yes	Approximate duration (in days)	Seriousness today:<br>[1] I could do everything that I usually do<br>[2] I could not do some of what I usually do<br>[3] I could not do some of what I usually do most of what I usually do
Fever	[0] No<br>[1] Yes	Approximate duration (in days)	Seriousness today:<br>[1] I could do everything that I usually do<br>[2] I could not do some of what I usually do<br>[3] I could not do some of what I usually do most of what I usually do
Body ache	[0] No<br>[1] Yes	Approximate duration (in days)	Seriousness today:<br>[1] I could do everything that I usually do<br>[2] I could not do some of what I usually do

			[3] I could not do some of what I usually do most of what I usually do
Diarrhea	[0] No<br>[1] Yes	Approximate duration (in days)	Seriousness today:<br>[1] I could do everything that I usually do<br>[2] I could not do some of what I usually do<br>[3] I could not do some of what I usually do most of what I usually do
Nausea/vomiting	[0] No<br>[1] Yes	Approximate duration (in days)	Seriousness today:<br>[1] I could do everything that I usually do<br>[2] I could not do some of what I usually do<br>[3] I could not do some of what I usually do most of what I usually do
Painful muscle and joints	[0] No<br>[1] Yes	Approximate duration (in days)	Seriousness today:<br>[1] I could do everything that I usually do<br>[2] I could not do some of

			what I usually do<br>[3] I could not do some of what I usually do most of what I usually do
Loss of smell	[0] No<br>[1] Yes	Approximate duration (in days)	Seriousness today:<br>[1] I could do everything that I usually do<br>[2] I could not do some of what I usually do<br>[3] I could not do some of what I usually do most of what I usually do
Loss of taste	[0] No<br>[1] Yes	Approximate duration (in days)	Seriousness today:<br>[1] I could do everything that I usually do<br>[2] I could not do some of what I usually do<br>[3] I could not do some of what I usually do most of what I usually do
Tiredness and fatigue	[0] No<br>[1] Yes	Approximate duration (in days)	Seriousness today:<br>[1] I could do everything that I usually do

			[2] I could not do some of what I usually do<br>[3] I could not do some of what I usually do most of what I usually do
Chills	[0] No<br>[1] Yes	Approximate duration (in days)	Seriousness today:<br>[1] I could do everything that I usually do<br>[2] I could not do some of what I usually do<br>[3] I could not do some of what I usually do most of what I usually do
Headache	[0] No<br>[1] Yes	Approximate duration (in days)	Seriousness today:<br>[1] I could do everything that I usually do<br>[2] I could not do some of what I usually do<br>[3] I could not do some of what I usually do most of what I usually do
Irritability or confusion	[0] No<br>[1] Yes	Approximate duration (in days)	Seriousness today:<br>[1] I could do everything

			that I usually do<br>[2] I could not do some of what I usually do<br>[3] I could not do some of what I usually do most of what I usually do
General weakness	[0] No<br>[1] Yes	Approximate duration (in days)	Seriousness today:<br>[1] I could do everything that I usually do<br>[2] I could not do some of what I usually do<br>[3] I could not do some of what I usually do most of what I usually do
Skin rash	[0] No<br>[1] Yes	Approximate duration (in days)	Seriousness today:<br>[1] I could do everything that I usually do<br>[2] I could not do some of what I usually do<br>[3] I could not do some of what I usually do most of what I usually do

21	When did the learner's symptoms (signs) first present?	dd/mm/yyyy

22	**In the last month, has the learner had close contact with someone with suspected COVID-19, confirmed COVID-19, flu or a cold?**<br>*Note: Close contact means the learner ate with or played with this person. They were close together for at least 15 minutes*	A suspected COVID-19 patient<br>[0] No [1] Yes<br>A confirmed COVID-19 patient<br>[0] No [1] Yes<br>Someone with the "flu" or "cold"<br>[0] No [1] Yes
23	If yes for any of the above, what setting was the contact:<br>*(Please select the answer that applies)*	[1] Healthcare setting<br>[2] Family setting<br>[3] School setting<br>[4] Public transport setting<br>[5] Other<br>*If other, please specify.*
24	Did the learner quarantine after the contact?	[0] No<br>[1] Yes
25	If yes, for how long did the learner quarantine?	____________days
26	If no, how many contacts did the learner have since that time?	
27	Has the learner been diagnosed with COVID-19 before?	[0] No<br>[1] Yes
28	If yes, how many times?	
29	Which was their most recent time? Give an approximate date of diagnosis	dd/mm/yyyy
30	Over the past 2 years has the learner felt sick or more tired, or had headaches or lost his/her taste or had COVID-19-like symptoms (signs) for 28-days or longer?	[0] No<br>[1] Yes

**Learner: Long COVID**<br>*(only answer question, if answer to question 308 was yes)*	
31	Tell us which signs/symptoms were present for more than 28-days.
Fatigue<br>[0] No<br>[1] Yes	
Stuffy/runny nose<br>[0] No<br>[1] Yes	Chest tightness<br>[0] No<br>[1] Yes
Chest pain<br>[0] No<br>[1] Yes	Cough<br>[0] No<br>[1] Yes
Wheezing	Sore throat

[0] No<br>[1] Yes	[0] No<br>[1] Yes
Muscle ache<br>[0] No<br>[1] Yes	Joint pain/swelling<br>[0] No<br>[1] Yes
Headache<br>[0] No<br>[1] Yes	Dizziness<br>[0] No<br>[1] Yes
Altered sense of taste (change in taste)<br>[0] No<br>[1] Yes	Altered sense of smell<br>[0] No<br>[1] Yes
Difficulty concentrating (focusing)<br>[0] No<br>[1] Yes	Sleep disorders<br>[0] No<br>[1] Yes
Mood alterations<br>[0] No<br>[1] Yes	Cognitive dysfunction (loss of memory or difficulty processing information or paying attention)<br>[0] No<br>[1] Yes
Sensorimotor symptoms (tingling in the toes / feet / legs / fingers / hands or twitching of muscles)<br>[0] No<br>[1] Yes	Increased need for sleep<br>[0] No<br>[1] Yes
Weight loss<br>[0] No<br>[1] Yes	Diarrhea<br>[0] No<br>[1] Yes
Stomach pain<br>[0] No<br>[1] Yes	Poor appetite<br>[0] No<br>[1] Yes
Constipation<br>[0] No<br>[1] Yes	Skin rash<br>[0] No<br>[1] Yes
Tachycardia<br>(fast heart rate)<br>[0] No<br>[1] Yes	Fever<br>[0] No<br>[1] Yes
Other<br>*If other, please specify.*<br>*Please provide space to specify*	

32	If, you have ticked at least one symptom (sign) above. Please select what applies:1	[1] A doctor had to be consulted because of this<br>[2] He/she had to stay away from school<br>*(If applicable answer question 33)*<br>[3] He/she had to be treated with medication<br>*(If applicable answer question 34)*<br>[4] He/she had to be hospitalized<br>*(If applicable answer question 35)*<br>[5] He/she has not done anything<br>[6] Other<br>*If other, please specify.*<br>*Please provide space to specify.*<br>*(multiple answers possible)*
33	How many days did the learner have to miss school?	____*(Please enter number in days)*
34	How many days did the learner have to be treated with medication?	____*(Please enter number in days)*
35	How many days did the learner have to spend in the hospital?	____*(Please enter number in days)*

**Learner (≥ 12 years of age): COVID-19 vaccination and vaccine hesitancy**<br>*These questions are applicable for learners that are eligible for the COVID-19 vaccination (≥ 12 years of age)*		
36	Has the learner been vaccinated against COVID-19?	[0] No<br>[1] Yes
37	If the learner has not been vaccinated, we would be interested to know why not?	[1] It is a choice and the learner or learner's parent/primary caregiver choose not too<br>[2] No time<br>[3] In general, the learner's family is against all vaccinations<br>[4] No expected benefit (vaccination does not work at all or not sufficiently)

<table>
<tr><td></td><td></td><td>[5] The learner or learner’s parent/primary caregiver does not trust the vaccine manufacturing companies
[6] The learner or learner’s parent/primary caregiver does not trust the government’s ability to roll out a safe vaccine.
[7] The learner or learner’s parent/primary caregiver wants to wait until there is more knowledge
[8] The learner or learner’s parent/primary caregiver fear the side effects, safety and effectiveness of vaccinations
[9] Due to the learner or learner’s parnet/primary caregiver religious or cultural beliefs
[10] The learner is afraid of needles
[11] The learner had COVID-19, so I do not consider the vaccination necessary
[13] The learner’s parent/primary caregiver just won’t let them take it.
[14] Due to the learner’s medical condition
[15] Other
If other, please specify
Please provide space to specify.

More than 1 answer allowed.</td></tr>
<tr><td>38</td><td>If yes, when did the learner receive their first dose? Give an approximate date</td><td>dd/mm/yyyy</td></tr>
<tr><td>39</td><td>Which vaccine did the learner receive?</td><td>[1] Pfizer
[2] CoronaVac
[3] Other (specify)
[4] Do not know</td></tr>
<tr><td>40</td><td>How many doses did the learner receive?
Circle the correct answer</td><td><table>
<tr><td></td><td>Pfizer</td><td>0</td><td>1</td><td>2</td><td>3</td></tr>
<tr><td></td><td>CoronaVac</td><td>0</td><td>1</td><td>2</td><td>3</td></tr>
<tr><td></td><td>Other (specify)</td><td></td><td></td><td></td><td></td></tr>
</table></td></tr>
</table>

41	Date of last (most recent) dose?	dd/mm/yyyy
**Learner: Chronic illness**		
42	Does the learner have any of the following complications?	

HIV<br>[0] No<br>[1] Yes	Current TB<br>[0] No<br>[1] Yes	Chronic Kidney Disease<br>[0] No<br>[1] Yes	Chronic Liver Disease<br>[0] No<br>[1] Yes
Neurological/neuromuscular disease<br>[0] No<br>[1] Yes	Diabetes Mellitus (high blood sugar)<br>[0] No<br>[1] Yes	Heart Disease<br>[0] No<br>[1] Yes	Cancer<br>[0] No<br>[1] Yes
Prior TB infection<br>[0] No<br>[1] Yes	Hypertension (high blood pressure)<br>[0] No<br>[1] Yes	Asthma (Difficulty breathing)<br>[0] No<br>[1] Yes	Chronic Lung Disease<br>[0] No<br>[1] Yes
Rheumatological disease (disease of the joints and muscles)<br>[0] No<br>[1] Yes	Obesity/overweight<br>[0] No<br>[1] Yes	Autoimmune disease (not HIV. A disease whereby our immune system starts attacking our own tissues or organs e.g., SLE)<br>[0] No<br>[1] Yes	Other 1: Details<br>Other 2: Details<br>Other 3: Details<br>Other 4: Details

43	Is the learner currently taking any of the following medications NOW:

Steroids<br>(e.g., Prednisone, cortisone)<br>[0] No<br>[1] Yes<br>[2] Prefer not to answer	Anti-inflammatories<br>(e.g., high dose aspirin, ibuprofen)<br>[0] No<br>[1] Yes<br>[2] Prefer not to answer
Anti-hypertensives<br>(blood pressure medication e.g., indapamide)<br>[0] No<br>[1] Yes<br>[2] Prefer not to answer	Chemotherapy<br>(cancer treatment)<br>[0] No<br>[1] Yes<br>[2] Prefer not to answer
Hormonal treatment<br>[0] No<br>[1] Yes<br>[2] Prefer not to answer	Antibiotics<br>(e.g., penicillin, amoxicillin)<br>[0] No<br>[1] Yes

	[2] Prefer not to answer
ARV/ART<br>[0] No<br>[1] Yes<br>[2] Prefer not to answer	Bactrim prophylaxis<br>[0] No<br>[1] Yes<br>[2] Prefer not to answer
Aspirin / Warfarin / Heparin<br>[0] No<br>[1] Yes<br>[2] Prefer not to answer	TB Meds<br>[0] No<br>[1] Yes<br>[2] Prefer not to answer
Other 1: Details<br>Other 2: Details<br>Other 3: Details	

44	What non pharmaceutical measures does the learner currently use to prevent COVID-19?		
Masks in public places e.g., buses and taxis<br>[0] No<br>[1] Yes always<br>[2] Yes – sometimes<br>[99] Not applicable (N/A)	Sanitizing<br>[0] No<br>[1] Yes always<br>[2] Yes – sometimes<br>[99] N/A	Masks in the school setting?<br>[0] No<br>[1] Yes always<br>[2] Yes – sometimes<br>[99] N/A	Distancing- - more than 1.5 meters away in the school setting?<br>[0] No<br>[1] Yes always<br>[2] Yes – sometimes<br>[99] N/A
Avoiding social gatherings/outings<br>[0] No<br>[1] Yes always<br>[2] Yes – sometimes<br>[99] N/A	Avoiding weddings<br>[0] No<br>[1] Yes always<br>[2] Yes – sometimes<br>[99] N/A	Avoiding funerals<br>[0] No<br>[1] Yes always<br>[2] Yes – sometimes<br>[99] N/A	
**Information about people in the learner's house**			
A household is a group of persons who live together and provide themselves jointly with food and/or other essentials for living, or a single person who lives alone.<br><br>We would like to ask some question about your child's household(s) and household members. The following question help in understanding the importance of people's living circumstances related to COVID-19.<br>By household, we mean the group of people your child lives with, in a single house or dwelling.			
45	Does the learner spend time living across more than one household within a week?	[0] No<br>[1] Yes	
Your child may spend time living across two households regularly within a week. The following questions relate to their primary household and house, where your child spends most of their time:			
46	In addition to the learner, how many people		

	are in this household?	
47	How many rooms does the learner's house have in total (please exclude any kitchens or bathrooms)?	
48	How many rooms in the learner's house are regularly used for sleeping?	
Could you please provide us with some details including any history of COVID- 19-like symptoms (signs) among other household members (excluding {child's name}). The information will help us understand the circumstances around your child's test results. *PLEASE PROCEED FROM THE YOUNGEST TO THE OLDEST MEMBER OF THE HOUSEHOLD;* ***If the child lives between more than one household, please also provide details for members of all households:***		
	**Household Member 1:**<br>*This section is repeated to allow for up to 10 household members.*	
49	Relationship to learner:	[1] Spouse/Partner<br>[2] Child/Stepchild<br>[3] Mother/Mother-in-law<br>[4] Father/Father-in-law<br>[5] Brother/Stepbrother<br>[6] Sister/Stepsister<br>[7]Grand parent<br>[9] Aunt<br>[10] Uncle<br>[11] Other relative, please specify_______<br>[12] No relation/ House mate
50	What is their gender?	[1] Male<br>[2] Female<br>[3] Other<br>[4] Prefer not to answer
51	What is their date of birth?	dd/mm/yyyy
52	If date of birth is unknown, what age-group are they in?	[1] 0-4<br>[2] 5-9<br>[3] 10-14<br>[4] 15-19<br>[5] 20-24<br>[6] 25-29<br>[7] 30-34<br>[8] 35-39<br>[9] 40-44<br>[10] 45-49

		[11] 50-54<br>[12] 55-59<br>[13] 60-64<br>[14] 65-69<br>[5] 70-74<br>[6] 75-79<br>[7] 80-84<br>[8] 85-89<br>[9] 90 and above
53	What is their working status?	[1] Employed, Full-time<br>[2] Employed, Part-time<br>[3] Unemployed<br>[4] Other<br>[5] Retired<br>[6] Child < 5 attending nursery school, pre-school or child minder<br>[7] Child > 5 attending formal school
54	Have they ever tested positive for a COVID-19 test?	[0] No<br>[1] Yes<br>[2] Do not know
55	If yes - date/month of the test?	
	**Household Member 2:**<br>*This section is repeated to allow for up to 10 household members.*	
56	Relationship to learner:	[1] Spouse/Partner<br>[2] Child/Stepchild<br>[3] Mother/Mother-in-law<br>[4] Father/Father-in-law<br>[5] Brother/Stepbrother<br>[6] Sister/Stepsister<br>[7]Grand parent<br>[9] Aunt<br>[10] Uncle

		[11] Other relative, please specify_______<br>[12] No relation/ House mate
57	What is their gender?	[1] Male<br>[2] Female<br>[3] Other<br>[4] Prefer not to answer
58	What is their date of birth?	dd/mm/yyyy
59	If date of birth is unknown, what age-group are they in?	[1] 0-4<br>[2] 5-9<br>[3] 10-14<br>[4] 15-19<br>[5] 20-24<br>[6] 25-29<br>[7] 30-34<br>[8] 35-39<br>[9] 40-44<br>[10] 45-49<br>[11] 50-54<br>[12] 55-59<br>[13] 60-64<br>[14] 65-69<br>[5] 70-74<br>[6] 75-79<br>[7] 80-84<br>[8] 85-89<br>[9] 90 and above
60	What is their working status?	[1] Employed, Full-time<br>[2] Employed, Part-time<br>[3] Unemployed<br>[4] Other<br>[5] Retired<br>[6] Child < 5 attending nursery school, pre-school or child minder<br>[7] Child > 5 attending formal school

61	Have they ever tested positive for a COVID-19 test?	[0] No<br>[1] Yes<br>[2] Do not know
62	If yes - date/month of the test?	
	**Household Member 3:**<br>*This section is repeated to allow for up to 10 household members.*	
63	Relationship to learner:	[1] Spouse/Partner<br>[2] Child/Stepchild<br>[3] Mother/Mother-in-law<br>[4] Father/Father-in-law<br>[5] Brother/Stepbrother<br>[6] Sister/Stepsister<br>[7]Grand parent<br>[9] Aunt<br>[10] Uncle<br>[11] Other relative, please specify______<br>[12] No relation/ House mate
64	What is their gender?	[1] Male<br>[2] Female<br>[3] Other<br>[4] Prefer not to answer
65	What is their date of birth?	dd/mm/yyyy
66	If date of birth is unknown, what age-group are they in?	[1] 0-4<br>[2] 5-9<br>[3] 10-14<br>[4] 15-19<br>[5] 20-24<br>[6] 25-29<br>[7] 30-34<br>[8] 35-39<br>[9] 40-44<br>[10] 45-49<br>[11] 50-54<br>[12] 55-59<br>[13] 60-64<br>[14] 65-69<br>[5] 70-74<br>[6] 75-79<br>[7] 80-84<br>[8] 85-89

		[9] 90 and above
67	What is their working status?	[1] Employed, Full-time<br>[2] Employed, Part-time<br>[3] Unemployed<br>[4] Other<br>[5] Retired<br>[6] Child < 5 attending nursery school, pre-school or child minder<br>[7] Child > 5 attending formal school
68	Have they ever tested positive for a COVID-19 test?	[0] No<br>[1] Yes<br>[2] Do not know
69	If yes - date/month of the test?	
	**Household Member 4:**<br>*This section is repeated to allow for up to 10 household members.*	
70	Relationship to learner:	[1] Spouse/Partner<br>[2] Child/Stepchild<br>[3] Mother/Mother-in-law<br>[4] Father/Father-in-law<br>[5] Brother/Stepbrother<br>[6] Sister/Stepsister<br>[7]Grand parent<br>[9] Aunt<br>[10] Uncle<br>[11] Other relative, please specify_______<br>[12] No relation/ House mate
71	What is their gender?	[1] Male<br>[2] Female<br>[3] Other<br>[4] Prefer not to answer

72	What is their date of birth?	dd/mm/yyyy
73	If date of birth is unknown, what age-group are they in?	[1] 0-4<br>[2] 5-9<br>[3] 10-14<br>[4] 15-19<br>[5] 20-24<br>[6] 25-29<br>[7] 30-34<br>[8] 35-39<br>[9] 40-44<br>[10] 45-49<br>[11] 50-54<br>[12] 55-59<br>[13] 60-64<br>[14] 65-69<br>[5] 70-74<br>[6] 75-79<br>[7] 80-84<br>[8] 85-89<br>[9] 90 and above
74	What is their working status?	[1] Employed, Full-time<br>[2] Employed, Part-time<br>[3] Unemployed<br>[4] Other<br>[5] Retired<br>[6] Child < 5 attending nursery school, pre-school or child minder<br>[7] Child > 5 attending formal school
75	Have they ever tested positive for a COVID-19 test?	[0] No<br>[1] Yes<br>[2] Do not know
76	If yes - date/month of the test?	
	**Household Member 5:**<br>*This section is repeated to allow for up to 10 household members.*	
77	Relationship to learner:	[1] Spouse/Partner<br>[2] Child/Stepchild

		[3] Mother/Mother-in-law<br>[4] Father/Father-in-law<br>[5] Brother/Stepbrother<br>[6] Sister/Stepsister<br>[7]Grand parent<br>[9] Aunt<br>[10] Uncle<br>[11] Other relative, please specify______<br>[12] No relation/ House mate
78	What is their gender?	[1] Male<br>[2] Female<br>[3] Other<br>[4] Prefer not to answer
79	What is their date of birth?	dd/mm/yyyy
80	If date of birth is unknown, what age-group are they in?	[1] 0-4<br>[2] 5-9<br>[3] 10-14<br>[4] 15-19<br>[5] 20-24<br>[6] 25-29<br>[7] 30-34<br>[8] 35-39<br>[9] 40-44<br>[10] 45-49<br>[11] 50-54<br>[12] 55-59<br>[13] 60-64<br>[14] 65-69<br>[5] 70-74<br>[6] 75-79<br>[7] 80-84<br>[8] 85-89<br>[9] 90 and above
81	What is their working status?	[1] Employed, Full-time<br>[2] Employed, Part-time<br>[3] Unemployed

		[4] Other<br>[5] Retired<br>[6] Child < 5 attending nursery school, pre-school or child minder<br>[7] Child > 5 attending formal school
82	Have they ever tested positive for a COVID-19 test?	[0] No<br>[1] Yes<br>[2] Do not know
83	If yes - date/month of the test?	
	**Household Member 6:**<br>*This section is repeated to allow for up to 10 household members.*	
84	Relationship to learner:	[1] Spouse/Partner<br>[2] Child/Stepchild<br>[3] Mother/Mother-in-law<br>[4] Father/Father-in-law<br>[5] Brother/Stepbrother<br>[6] Sister/Stepsister<br>[7]Grand parent<br>[9] Aunt<br>[10] Uncle<br>[11] Other relative, please specify______<br>[12] No relation/ House mate
85	What is their gender?	[1] Male<br>[2] Female<br>[3] Other<br>[4] Prefer not to answer
86	What is their date of birth?	dd/mm/yyyy
87	If date of birth is unknown, what age-group are they in?	[1] 0-4<br>[2] 5-9<br>[3] 10-14<br>[4] 15-19<br>[5] 20-24<br>[6] 25-29<br>[7] 30-34

		[8] 35-39<br>[9] 40-44<br>[10] 45-49<br>[11] 50-54<br>[12] 55-59<br>[13] 60-64<br>[14] 65-69<br>[5] 70-74<br>[6] 75-79<br>[7] 80-84<br>[8] 85-89<br>[9] 90 and above
88	What is their working status?	[1] Employed, Full-time<br>[2] Employed, Part-time<br>[3] Unemployed<br>[4] Other<br>[5] Retired<br>[6] Child < 5 attending nursery school, pre-school or child minder<br>[7] Child > 5 attending formal school
89	Have they ever tested positive for a COVID-19 test?	[0] No<br>[1] Yes<br>[2] Do not know
90	If yes - date/month of the test?	
	**Household Member 7:**<br>*This section is repeated to allow for up to 10 household members.*	
91	Relationship to learner:	[1] Spouse/Partner<br>[2] Child/Stepchild<br>[3] Mother/Mother-in-law<br>[4] Father/Father-in-law<br>[5] Brother/Stepbrother<br>[6] Sister/Stepsister<br>[7]Grand parent<br>[9] Aunt

		[10] Uncle<br>[11] Other relative, please specify_______<br>[12] No relation/ House mate
92	What is their gender?	[1] Male<br>[2] Female<br>[3] Other<br>[4] Prefer not to answer
93	What is their date of birth?	dd/mm/yyyy
94	If date of birth is unknown, what age-group are they in?	[1] 0-4<br>[2] 5-9<br>[3] 10-14<br>[4] 15-19<br>[5] 20-24<br>[6] 25-29<br>[7] 30-34<br>[8] 35-39<br>[9] 40-44<br>[10] 45-49<br>[11] 50-54<br>[12] 55-59<br>[13] 60-64<br>[14] 65-69<br>[5] 70-74<br>[6] 75-79<br>[7] 80-84<br>[8] 85-89<br>[9] 90 and above
95	What is their working status?	[1] Employed, Full-time<br>[2] Employed, Part-time<br>[3] Unemployed<br>[4] Other<br>[5] Retired<br>[6] Child < 5 attending nursery school, pre-school or child minder

		[7] Child > 5 attending formal school
96	Have they ever tested positive for a COVID-19 test?	[0] No<br>[1] Yes<br>[2] Do not know
97	If yes - date/month of the test?	
	**Household Member 8:**<br>*This section is repeated to allow for up to 10 household members.*	
98	Relationship to learner:	[1] Spouse/Partner<br>[2] Child/Stepchild<br>[3] Mother/Mother-in-law<br>[4] Father/Father-in-law<br>[5] Brother/Stepbrother<br>[6] Sister/Stepsister<br>[7]Grand parent<br>[9] Aunt<br>[10] Uncle<br>[11] Other relative, please specify_______<br>[12] No relation/ House mate
99	What is their gender?	[1] Male<br>[2] Female<br>[3] Other<br>[4] Prefer not to answer
100	What is their date of birth?	dd/mm/yyyy
1019	If date of birth is unknown, what age-group are they in?	[1] 0-4<br>[2] 5-9<br>[3] 10-14<br>[4] 15-19<br>[5] 20-24<br>[6] 25-29<br>[7] 30-34<br>[8] 35-39<br>[9] 40-44<br>[10] 45-49<br>[11] 50-54<br>[12] 55-59<br>[13] 60-64<br>[14] 65-69<br>[5] 70-74

		[6] 75-79<br>[7] 80-84<br>[8] 85-89<br>[9] 90 and above
102	What is their working status?	[1] Employed, Full-time<br>[2] Employed, Part-time<br>[3] Unemployed<br>[4] Other<br>[5] Retired<br>[6] Child < 5 attending nursery school, pre-school or child minder<br>[7] Child > 5 attending formal school
103	Have they ever tested positive for a COVID-19 test?	[0] No<br>[1] Yes<br>[2] Do not know
104	If yes - date/month of the test?	
	**Household Member 9:**<br>*This section is repeated to allow for up to 10 household members.*	
105	Relationship to learner:	[1] Spouse/Partner<br>[2] Child/Stepchild<br>[3] Mother/Mother-in-law<br>[4] Father/Father-in-law<br>[5] Brother/Stepbrother<br>[6] Sister/Stepsister<br>[7]Grand parent<br>[9] Aunt<br>[10] Uncle<br>[11] Other relative, please specify_______<br>[12] No relation/ House mate
106	What is their gender?	[1] Male<br>[2] Female

		[3] Other<br>[4] Prefer not to answer
107	What is their date of birth?	dd/mm/yyyy
108	If date of birth is unknown, what age-group are they in?	[1] 0-4<br>[2] 5-9<br>[3] 10-14<br>[4] 15-19<br>[5] 20-24<br>[6] 25-29<br>[7] 30-34<br>[8] 35-39<br>[9] 40-44<br>[10] 45-49<br>[11] 50-54<br>[12] 55-59<br>[13] 60-64<br>[14] 65-69<br>[5] 70-74<br>[6] 75-79<br>[7] 80-84<br>[8] 85-89<br>[9] 90 and above
109	What is their working status?	[1] Employed, Full-time<br>[2] Employed, Part-time<br>[3] Unemployed<br>[4] Other<br>[5] Retired<br>[6] Child < 5 attending nursery school, pre-school or child minder<br>[7] Child > 5 attending formal school
110	Have they ever tested positive for a COVID-19 test?	[0] No<br>[1] Yes<br>[2] Do not know
111	If yes - date/month of the test?	
	**Household Member 10:**	

	This section is repeated to allow for up to 10 household members.	
112	Relationship to learner:	[1] Spouse/Partner<br>[2] Child/Stepchild<br>[3] Mother/Mother-in-law<br>[4] Father/Father-in-law<br>[5] Brother/Stepbrother<br>[6] Sister/Stepsister<br>[7]Grand parent<br>[9] Aunt<br>[10] Uncle<br>[11] Other relative, please specify______<br>[12] No relation/ House mate
113	What is their gender?	[1] Male<br>[2] Female<br>[3] Other<br>[4] Prefer not to answer
114	What is their date of birth?	dd/mm/yyyy
115	If date of birth is unknown, what age-group are they in?	[1] 0-4<br>[2] 5-9<br>[3] 10-14<br>[4] 15-19<br>[5] 20-24<br>[6] 25-29<br>[7] 30-34<br>[8] 35-39<br>[9] 40-44<br>[10] 45-49<br>[11] 50-54<br>[12] 55-59<br>[13] 60-64<br>[14] 65-69<br>[5] 70-74<br>[6] 75-79<br>[7] 80-84<br>[8] 85-89<br>[9] 90 and above
116	What is their working status?	[1] Employed, Full-time

		[2] Employed, Part-time<br>[3] Unemployed<br>[4] Other<br>[5] Retired<br>[6] Child < 5 attending nursery school, pre-school or child minder<br>[7] Child > 5 attending formal school
117	Have they ever tested positive for a COVID-19 test?	[0] No<br>[1] Yes<br>[2] Do not know
118	If yes - date/month of the test?	

**Learner: Specimen collection**<br>*This section is to be completed by study staff collecting the specimens from the learner*		
119	Date of specimen collection?	dd/mm/yyyy
120	Was blood collected for Rapid COVID-19 antibody POC test?	[0] No<br>[1] Yes<br>[99] N/A
121	If yes, what was the name of the test?	[1] Orient gene<br>[2] Other<br>*If other, please specify*
122	What was the result?<br>*(more than one answer allowed)*	[1] Indeterminate<br>[2] IgG positive<br>[3] IgM positive<br>[99] N/A
123	Was blood collected for DBS?	[0] No<br>[1] Yes<br>[99] N/A
124	Was saliva collected for future testing?	[0] No<br>[1] Yes<br>[99] N/A

**Learner: Mental health**		
125	My child feels empty	[1] Never<br>[2] Sometimes

		[3] Often<br>[4] Always
126	My child feels afraid of being alone at home	[1] Never<br>[2] Sometimes<br>[3] Often<br>[4] Always
127	My child worries when he/she think he/she has done poorly at something	[1] Never<br>[2] Sometimes<br>[3] Often<br>[4] Always
128	Nothing is much fun for my child anymore	[1] Never<br>[2] Sometimes<br>[3] Often<br>[4] Always
129	My child worries that something awful will happen to someone in the family	[1] Never<br>[2] Sometimes<br>[3] Often<br>[4] Always
130	My child is afraid of being in crowded places like shopping centers, the movies, buses, playgrounds	[1] Never<br>[2] Sometimes<br>[3] Often<br>[4] Always
131	My child worries what other people think of him/her	[1] Never<br>[2] Sometimes<br>[3] Often<br>[4] Always
132	My child has trouble sleeping	[1] Never<br>[2] Sometimes<br>[3] Often<br>[4] Always
133	My child feels scared to sleep on his/her own	[1] Never<br>[2] Sometimes<br>[3] Often<br>[4] Always
134	My child has problems with his/her appetite	[1] Never<br>[2] Sometimes<br>[3] Often<br>[4] Always
135	My child feels dizzy or faints when there is no reason for this	[1] Never<br>[2] Sometimes<br>[3] Often<br>[4] Always
136	My child has to do some things over and over again (like washing hands, cleaning or putting things in a certain order)	[1] Never<br>[2] Sometimes<br>[3] Often<br>[4] Always

137	My child has no energy for things	[1] Never<br>[2] Sometimes<br>[3] Often<br>[4] Always
138	My child cannot think clearly	[1] Never<br>[2] Sometimes<br>[3] Often<br>[4] Always
139	My child suddenly starts to tremble or shake when there is no reason for this	[1] Never<br>[2] Sometimes<br>[3] Often<br>[4] Always
140	My child feels worthless	[1] Never<br>[2] Sometimes<br>[3] Often<br>[4] Always
141	My child has to think of special thoughts (like number or words) to stop bad things from happening	[1] Never<br>[2] Sometimes<br>[3] Often<br>[4] Always
142	My child thinks about death	[1] Never<br>[2] Sometimes<br>[3] Often<br>[4] Always
143	My child feels like he/she does not want to move	[1] Never<br>[2] Sometimes<br>[3] Often<br>[4] Always
144	My child worries that he/she will suddenly get a scared feeling when there is nothing to be afraid of	[1] Never<br>[2] Sometimes<br>[3] Often<br>[4] Always
145	My child is tired alot	[1] Never<br>[2] Sometimes<br>[3] Often<br>[4] Always
146	My child feels like he/she will make a fool of him/her self in front of people	[1] Never<br>[2] Sometimes<br>[3] Often<br>[4] Always
147	My child has to do some things in just the right way to stop bad things from happening	[1] Never<br>[2] Sometimes<br>[3] Often<br>[4] Always
148	My child feels restless	[1] Never<br>[2] Sometimes

		[3] Often<br>[4] Always
149	My child worries that something bad will happen to him or her	[1] Never<br>[2] Sometimes<br>[3] Often<br>[4] Always

FOLLOW-UP SURVEY:
CRF FOR PARENT/PRIMARY CAREGIVER OF LEARNER IN GRADE 1-7

	Instructions:<br>1. *All instructions are in italics.*<br>2. *In this study, parent also refers to primary caregiver.*	
1	Visit Code	
2	Research staff ID	
3	Does the child have an SA ID or passport?	
4	What is the child's SA ID or passport number?	
5	Study unique identifier (Child)<br>*This question will be completed when the form returned to the school.*<br>*RA must ensure that they have filled/ completed the link log with the study ID and name of participant.*	
6	Re-enter study unique identifier<br>*This question will be completed when the form is and returned to the school.*<br>*RA to complete.*	
7	Today's date	dd/mm/yyyy
8	What is the name of the school your child attends?	
9	What grade is your child in?	[1] Grade 1<br>[2] Grade 2<br>[3] Grade 3<br>[4] Grade 4<br>[5] Grade 5<br>[6] Grade 6<br>[7] Grade 7
10	What is the name of your child's class?	*Class ID to be codified.*
**Learner: Acute COVID-19 infection**		
11	Is the learner currently feeling sick?	[0] No<br>[1] Yes
12	Does the learner have any of the following symptoms now? If yes, indicate which symptoms are currently present, and approximate duration and severity	

Cough	[0] No<br>[1] Yes	Approximate duration (in days)	Seriousness today:<br>[1] I could do everything that I usually do<br>[2] I could not do some of what I usually do

			[3] I could not do some of what I usually do most of what I usually do
Sore Throat	[0] No<br>[1] Yes	Approximate duration (in days)	Seriousness today:<br>[1] I could do everything that I usually do<br>[2] I could not do some of what I usually do<br>[3] I could not do some of what I usually do most of what I usually do
Fever	[0] No<br>[1] Yes	Approximate duration (in days)	Seriousness today:<br>[1] I could do everything that I usually do<br>[2] I could not do some of what I usually do<br>[3] I could not do some of what I usually do most of what I usually do
Body ache	[0] No<br>[1] Yes	Approximate duration (in days)	Seriousness today:<br>[1] I could do everything that I usually do<br>[2] I could not do some of what I

			usually do<br>[3] I could not do some of what I usually do most of what I usually do
Diarrhea	[0] No<br>[1] Yes	Approximate duration (in days)	Seriousness today:<br>[1] I could do everything that I usually do<br>[2] I could not do some of what I usually do<br>[3] I could not do some of what I usually do most of what I usually do
Nausea/vomiting	[0] No<br>[1] Yes	Approximate duration (in days)	Seriousness today:<br>[1] I could do everything that I usually do<br>[2] I could not do some of what I usually do<br>[3] I could not do some of what I usually do most of what I usually do
Painful muscles and joints	[0] No<br>[1] Yes	Approximate duration (in days)	Seriousness today:<br>[1] I could do everything that I usually do<br>[2] I could

			not do some of what I usually do [3] I could not do some of what I usually do most of what I usually do
Loss of smell	[[0] No [1] Yes	Approximate duration (in days)	Seriousness today: [1] I could do everything that I usually do [2] I could not do some of what I usually do [3] I could not do some of what I usually do most of what I usually do
Loss of taste	[[0] No [1] Yes	Approximate duration (in days)	Seriousness today: [1] I could do everything that I usually do [2] I could not do some of what I usually do [3] I could not do some of what I usually do most of what I usually do
Tiredness and fatigue	[0] No [1] Yes	Approximate duration (in days)	Seriousness today: [1] I could do everything that I

			usually do<br>[2] I could not do some of what I usually do<br>[3] I could not do some of what I usually do most of what I usually do
Chills	[0] No<br>[1] Yes	Approximate duration (in days)	Seriousness today:<br>[1] I could do everything that I usually do<br>[2] I could not do some of what I usually do<br>[3] I could not do some of what I usually do most of what I usually do
Headache	[0] No<br>[1] Yes	Approximate duration (in days)	Seriousness today:<br>[1] I could do everything that I usually do<br>[2] I could not do some of what I usually do<br>[3] I could not do some of what I usually do most of what I usually do
Irritability or confusion	[0] No<br>[1] Yes	Approximate duration (in days)	Seriousness today:<br>[1] I could do

			everything that I usually do [2] I could not do some of what I usually do [3] I could not do some of what I usually do most of what I usually do
General weakness	[0] No [1] Yes	Approximate duration (in days)	Seriousness today: [1] I could do everything that I usually do [2] I could not do some of what I usually do [3] I could not do some of what I usually do most of what I usually do
Skin rash	[0] No [1] Yes	Approximate duration (in days)	Seriousness today: [1] I could do everything that I usually do [2] I could not do some of what I usually do [3] I could not do some of what I usually do most of what I usually do

13	When did the learner's symptoms (signs) first present?	dd/mm/yyyy
14	**In the last month, has the learner had close contact with someone with suspected COVID-19, confirmed COVID-19, flu or a cold?**<br>*Note: Close contact means the learner ate with or played with this person. They were close together for at least 15 minutes*	A suspected COVID-19 patient<br>[0] No …[1] Yes…<br>A confirmed COVID-19 patient<br>[0] No …[1] Yes…<br>Someone with the "flu" or "cold"<br>[0] No …[1] Yes…
15	If yes for any of the above, what setting was the contact:<br>*(Please select the answer that applies)*	[1] Healthcare setting<br>[2] Family setting<br>[3] School setting<br>[4] Public transport setting<br>[5] Other<br>*If other, please specify.*
16	Did the learner quarantine after the contact?	[0] No<br>[1] Yes
17	If yes, for how long did the learner quarantine?	____________days
18	If no, how many contacts did the learner have since that time?	
19	Has the learner been diagnosed with COVID-19 before?	[0] No<br>[1] Yes
20	If yes, how many times?	
21	Which was their most recent time? Give an approximate date of diagnosis	dd/mm/yyyy
22	Over the past 2 years has the learner felt sick or more tired, or had headaches or lost his/her taste or had COVID-19-like symptoms (signs) for 28-days or longer?	[[0] No<br>[1] Yes

**Learner: Long COVID**<br>*(Only answer question, if answer to question 22 was yes)*	
23 Tell us which signs/symptoms were present for more than 28-days.	
Fatigue<br>[0] No<br>[1] Yes	
Stuffy/runny nose<br>[0] No<br>[1] Yes	Chest tightness<br>[0] No<br>[1] Yes
Chest pain<br>[0] No<br>[1] Yes	Cough<br>[0] No<br>[1] Yes
Wheezing	Sore throat

[0] No<br>[1] Yes	[0] No<br>[1] Yes
Muscle ache<br>[0] No<br>[1] Yes	Joint pain/swelling<br>[0] No<br>[1] Yes
Headache<br>[0] No<br>[1] Yes	Dizziness<br>[0] No<br>[1] Yes
Altered sense of taste (change in taste)<br>[0] No<br>[1] Yes	Altered sense of smell<br>[0] No<br>[1] Yes
Difficulty concentrating (focusing)<br>[0] No<br>[1] Yes	Sleep disorders<br>[0] No<br>[1] Yes
Mood alterations<br>[0] No<br>[1] Yes	Cognitive dysfunction (loss of memory or difficulty processing information or paying attention)<br>[0] No<br>[1] Yes
Sensorimotor symptoms (tingling in the toes / feet / legs / fingers / hands or twitching of muscles)<br>[0] No<br>[1] Yes	Increased need for sleep<br>[0] No<br>[1] Yes
Weight loss<br>[0] No<br>[1] Yes	Diarrhea<br>[0] No<br>[1] Yes
Stomach pain<br>[0] No<br>[1] Yes	Poor appetite<br>[0] No<br>[1] Yes
Constipation<br>[0] No<br>[1] Yes	Skin rash<br>[0] No<br>[1] Yes
Tachycardia (fast heart rate)<br>[0] No<br>[1] Yes	Fever<br>[0] No<br>[1] Yes
Other<br>*If other, please specify.*<br>*Please provide space to specify*	

24	If, you have ticked at least one symptom (sign) above. Please select what applies:	[1] A doctor had to be consulted because of this<br>[2] He/she had to stay away from school<br>*(If applicable answer question 25)*<br>[3] He/she had to be treated with medication<br>*(If applicable answer question 26)*<br>[4] He/she had to be hospitalized<br>*(If applicable answer question 27)*<br>[5] He/she has not done anything<br>[6] Other<br>*If other, please specify.*<br>*Please provide space to specify.*<br>*(multiple answers possible)*
25	How many days did the learner have to miss school?	____*(Please enter number in days)*
26	How many days did the learner have to be treated with medication?	____*(Please enter number in days)*
27	How many days did the learner have to spend in the hospital?	____*(Please enter number in days)*

**Learner (≥ 12 years of age): COVID-19 vaccination and vaccine hesitancy**<br>*These questions are applicable for learners that are eligible for the COVID-19 vaccination (≥ 12 years of age)*		
28	Has the learner been vaccinated against COVID-19?	[0] No<br>[1] Yes
29	If the learner has not been vaccinated, we would be interested to know why not?	1] It is a choice and the learner or learner's parent/primary caregiver choose not too<br>[2] No time<br>[3] In general, the learner's family is against all vaccinations<br>[4] No expected benefit (vaccination does not work at all or not sufficiently)

		[5] The learner or learner's parent/primary caregiver does not trust the vaccine manufacturing companies<br>[6] The learner or learner's parent/primary caregiver does not trust the government's ability to roll out a safe vaccine.<br>[7] The learner or learner's parent/primary caregiver wants to wait until there is more knowledge<br>[8] The learner or learner's parent/primary caregiver fear the side effects, safety and effectiveness of vaccinations<br>[9] Due to the learner or learner's parnet/primary caregiver religious or cultural beliefs<br>[10] The learner is afraid of needles<br>[11] The learner had COVID-19, so I do not consider the vaccination necessary<br>[13] The learner's parent/primary caregiver just won't let them take it.<br>[14] Due to the learner's medical condition<br>[15] Other<br>If other, please specify<br>*Please provide space to specify.*<br><br>*More than 1 answer allowed.*
30	If yes, when did the learner receive their first dose? Give an approximate date	dd/mm/yyyy
31	Which vaccine did the learner receive?	[1] Pfizer<br>[2] CoronaVac<br>[3] Other (specify)<br>[4] Do not know
32	How many doses did the learner receive?<br>*Circle the correct answer*	<table><tr><td></td><td>Pfizer</td><td>0</td><td>1</td><td>2</td><td>3</td></tr><tr><td></td><td>CoronaVac</td><td>0</td><td>1</td><td>2</td><td>3</td></tr><tr><td></td><td>Other (specify)</td><td>0</td><td></td><td></td><td></td></tr></table>

33	Date of last (most recent) dose?	dd/mm/yyyy

Learner: Chronic illness

34	Does the learner have any of the following complications from the last survey?

HIV<br>[0] No<br>[1] Yes	Current TB<br>[0] No<br>[1] Yes	Chronic Kidney Disease<br>[0] No<br>[1] Yes	Chronic Liver Disease<br>[0] No<br>[1] Yes
Neurological/neuromuscular disease<br>[0] No<br>[1] Yes	Diabetes Mellitus (high blood sugar)<br>[0] No<br>[1] Yes	Heart Disease<br>[0] No<br>[1] Yes	Cancer<br>[0] No<br>[1] Yes
Prior TB infection<br>[0] No<br>[1] Yes	Hypertension (high blood pressure)<br>[0] No<br>[1] Yes	Asthma (Difficulty breathing)<br>[0] No<br>[1] Yes	Chronic Lung Disease<br>[0] No<br>[1] Yes
Rheumatological disease (disease of the joints and muscles)<br>[0] No<br>[1] Yes	Obesity/overweight<br>[0] No<br>[1] Yes	Autoimmune disease (not HIV. A disease whereby our immune system starts attacking our own tissues or organs e.g., SLE)<br>[0] No<br>[1] Yes	Other 1: Details<br>Other 2: Details<br>Other 3: Details<br>Other 4: Details

35	Is the learner currently taking any of the following medications NOW:

Steroids<br>(e.g., Prednisone, cortisone)<br>[0] No<br>[1] Yes<br>[2] Prefer not to answer	Anti-inflammatories<br>(e.g., high dose aspirin, ibuprofen)<br>[0] No<br>[1] Yes<br>[2] Prefer not to answer
Anti-hypertensives<br>(blood pressure medication e.g., indapamide)<br>[0] No<br>[1] Yes<br>[2] Prefer not to answer	Chemotherapy<br>(cancer treatment)<br>[0] No<br>[1] Yes<br>[2] Prefer not to answer
Hormonal treatment<br>[0] No<br>[1] Yes	Antibiotics<br>(e.g., penicillin, amoxicillin)

[2] Prefer not to answer	[0] No<br>[1] Yes<br>[2] Prefer not to answer
ARV/ART<br>[0] No<br>[1] Yes<br>[2] Prefer not to answer	Bactrim prophylaxis<br>[0] No<br>[1] Yes<br>[2] Prefer not to answer
Aspirin / Warfarin / Heparin<br>[0] No<br>[1] Yes<br>[2] Prefer not to answer	TB Meds<br>[0] No<br>[1] Yes<br>[2] Prefer not to answer
Other 1: Details<br>Other 2: Details<br>Other 3: Details	

36	What non pharmaceutical measures does the learner currently use to prevent COVID-19?		
Masks in public places e.g., buses and taxis<br>[0] No<br>[1] Yes always<br>[2] Yes – sometimes<br>[99] N/A	Sanitizing<br>[0] No<br>[1] Yes always<br>[2] Yes – sometimes<br>[99] N/A	Masks in the school setting?<br>[0] No<br>[1] Yes always<br>[2] Yes – sometimes<br>[99] N/A	Distancing- - more than 1.5 meters away in the school setting?<br>[0] No<br>[1] Yes always<br>[2] Yes – sometimes<br>[99] N/A
Avoiding social gatherings/outings<br>[0] No<br>[1] Yes always<br>[2] Yes – sometimes<br>[99] N/A	Avoiding weddings<br>[0] No<br>[1] Yes always<br>[2] Yes – sometimes<br>[99] N/A	Avoiding funerals<br>[0] No<br>[1] Yes always<br>[2] Yes – sometimes<br>[99] N/A	

**Learner: Specimen collection**<br>*This section is to be completed by study staff collecting the specimens from the learner*		
37	Date of specimen collection?	dd/mm/yyyy
38	Was blood collected for Rapid COVID-19 antibody POC test?	[0] No<br>[1] Yes<br>[99] N/A
39	If yes, what was the name of the test?	[1] Orient gene<br>[2] Other<br>*If other, please specify*
40	What was the result?<br>*(more than one answer allowed)*	[1] Indeterminate<br>[2] IgG positive<br>[3] IgM positive

		[99] N/A
41	Was blood collected for DBS?	[0] No<br>[1] Yes<br>[99] N/A
42	Was saliva collected for future testing?	[0] No<br>[1] Yes<br>[99] N/A

**Learner: Mental health**		
43	My child feels empty	[1] Never<br>[2] Sometimes<br>[3] Often<br>[4] Always
44	My child feels afraid of being alone at home	[1] Never<br>[2] Sometimes<br>[3] Often<br>[4] Always
45	My child worries when he/she think he/she has done poorly at something	[1] Never<br>[2] Sometimes<br>[3] Often<br>[4] Always
46	Nothing is much fun for my child anymore	[1] Never<br>[2] Sometimes<br>[3] Often<br>[4] Always
47	My child worries that something awful will happen to someone in the family	[1] Never<br>[2] Sometimes<br>[3] Often<br>[4] Always
48	My child is afraid of being in crowded places like shopping centers, the movies, buses, playgrounds	[1] Never<br>[2] Sometimes<br>[3] Often<br>[4] Always
49	My child worries what other people think of him/her	[1] Never<br>[2] Sometimes<br>[3] Often<br>[4] Always
50	My child has trouble sleeping	[1] Never<br>[2] Sometimes<br>[3] Often<br>[4] Always
51	My child feels scared to sleep on his/her own	[1] Never<br>[2] Sometimes<br>[3] Often

		[4] Always
52	My child has problems with his/her appetite	[1] Never<br>[2] Sometimes<br>[3] Often<br>[4] Always
53	My child feels dizzy or faints when there is no reason for this	[1] Never<br>[2] Sometimes<br>[3] Often<br>[4] Always
54	My child has to do some things over and over again (like washing hands, cleaning or putting things in a certain order)	[1] Never<br>[2] Sometimes<br>[3] Often<br>[4] Always
55	My child has no energy for things	[1] Never<br>[2] Sometimes<br>[3] Often<br>[4] Always
56	My child cannot think clearly	[1] Never<br>[2] Sometimes<br>[3] Often<br>[4] Always
57	My child suddenly starts to tremble or shake when there is no reason for this	[1] Never<br>[2] Sometimes<br>[3] Often<br>[4] Always
58	My child feels worthless	[1] Never<br>[2] Sometimes<br>[3] Often<br>[4] Always
59	My child has to think of special thoughts (like number or words) to stop bad things from happening	[1] Never<br>[2] Sometimes<br>[3] Often<br>[4] Always
60	My child thinks about death	[1] Never<br>[2] Sometimes<br>[3] Often<br>[4] Always
61	My child feels like he/she does not want to move	[1] Never<br>[2] Sometimes<br>[3] Often<br>[4] Always
62	My child worries that he/she will suddenly get a scared feeling when there is nothing to be afraid of	[1] Never<br>[2] Sometimes<br>[3] Often<br>[4] Always
63	My child is tired alot	[1] Never

		[2] Sometimes<br>[3] Often<br>[4] Always
64	My child feels like he/she will make a fool of him/her self in front of people	[1] Never<br>[2] Sometimes<br>[3] Often<br>[4] Always
65	My child has to do some things in just the right way to stop bad things from happening	[1] Never<br>[2] Sometimes<br>[3] Often<br>[4] Always
66	My child feels restless	[1] Never<br>[2] Sometimes<br>[3] Often<br>[4] Always
67	My child worries that something bad will happen to him or her	[1] Never<br>[2] Sometimes<br>[3] Often<br>[4] Always
