# Supplementary material for: SARS-CoV-2 Infections in a Triad of Primary School Learners (Grades 1-7), Their Parents, and Teachers in KwaZulu-Natal, South Africa: Protocol for a Cross-Sectional and Nested Case-Cohort Study
Source: JMIR Res Protoc. 2024 Dec 19;13:e52713. doi: 10.2196/52713 (PMC11695960; doi:10.2196/52713)
Supplement: Multimedia Appendix 5 [file resprot_v13i1e52713_app5.pdf]

COKIDSS NESTED CASE COHORT SUB STUDY:
eCRF FOR CLOSE CONTACT

Instructions:<br>1. *All instructions are in italics.*		
1	Visit code	
2	Research staff ID<br><br>*This refers to the staff member assisting the participant to complete this CRF.*	
3	Do you have an SA ID or passport number?	
4	What is your SA ID or passport number?	
6	Study unique identifier<br><br>*RA must ensure that they have filled/ completed the link log with the study ID and name of participant.*	
7	Re-enter study unique identifier	*RA to complete.*
8	Today's date	dd/mm/yyyy
9	Is the participant a close contact (<18 years of age), or a close contact (≥ 18 years of age)?	[1] Close contact (<18 years of age)<br><br>*(RA must ensure that parental consent and assent is obtained, prior to the completion of this CRF.)*<br><br>[2] Close contact (≥ 18 years of age)<br>*(Only consent from the participant is required prior to the completion of this CRF.)*
10	Are you presently a learner, parent or teacher participating in the study?	[1] Learner<br>[2] Parent<br>[3] Teacher<br>[4] Not applicable (N/A)
11	If you are a learner/parent/teacher already participating in the study, what is the name of the school that you or your child attends or teach?	
12	If you are a learner or parent of a learner, what grade are you/your child in?	[1] Grade 1<br>[2] Grade 2<br>[3] Grade 3<br>[4] Grade 4<br>[5] Grade 5<br>[6] Grade 6<br>[7] Grade 7
13	If you are teacher, what grade do you teach?	[1] Grade 1<br>[2] Grade 2<br>[3] Grade 3<br>[4] Grade 4

		[5] Grade 5<br>[6] Grade 6<br>[7] Grade 7
14	What is the name of the learner or teacher's class?<br>*Class ID to be CODIFIED.*	
15	What is your sex (Gender)?	[1] Male<br>[2] Female<br>[3] Other, *please specify*_____________________________
16	What is your ethnicity (Race)?	[1] Black African<br>[2] Indian<br>[3] Coloured<br>[4] White<br>[5] Other, *please specify*_______________________________
17	What is your current address (needed for tracing purposes) and suburb?	
18	What is your contact number?<br>*Must input 10 digits.*	
19	What is your alternate contact number?<br>*Must input 10 digits.*	
20	What is the name of the learner's /teacher's class?<br>*Class ID to be codified.*	
21	If you answered N/A to question 10 have you completed school, what is your highest level of education?	[1] No formal education<br>[2] Junior primary school (Grade 1-4)<br>[3] Senior primary school (Grade 5-7)<br>[4] Some secondary school (Grade 8-12)<br>[5] Completed secondary school with certificate (Grade 12)<br>[6] Some university/technical education<br>[7] Completed university/technical education<br>[8] National certificate/trade certificate/national diploma/occupational certificate<br>[9] Some graduate school (doctorate, masters, honors, higher education degree)<br>[10] Completed graduate school<br>[11] Prefer not to answer

22	If you answered N/A to question 10 are you working, what is your current work situation?	[1] Employed, part-time<br>[2] Employed, full-time<br>[2] Unemployed<br>[3] Other___________________________

Close Contact: Acute COVID-19 infection					
23	Are you currently feeling sick?			[0] No<br>[1] Yes	
24	Do you have any of the following symptoms now? If yes, indicate which symptoms are currently present, and approximate duration and severity				
	Cough	[0] No<br>[1] Yes	Approximate duration (in days)	Seriousness today<br>[1] I could do everything that I usually do<br>[2] I could not do some of what I usually do<br>[3] I could not do most of what I usually do	
	Sore throat	[0] No<br>[1] Yes	Approximate duration (in days)	Seriousness today:<br>[1] I could do everything that I usually do<br>[2] I could not do some of what I usually do<br>[3] I could not do most of what I usually do	
	Fever	[0] No<br>[1] Yes	Approximate duration (in days)	Seriousness today:<br>[1] I could do everything that I usually do<br>[2] I could not do some of what I usually do<br>[3] I could not do most of what I usually do	
	Body aches	[0] No<br>[1] Yes	Approximate duration (in days)	Seriousness today:<br>[1] I could do everything that I usually do<br>[2] I could not do some of what I usually do<br>[3] I could not do most of what I usually do	
	Diarrhea	[0] No<br>[1] Yes	Approximate duration (in days)	Seriousness today:<br>[1] I could do everything that I usually do<br>[2] I could not do some of what I usually do	

				[3] I could not do most of what I usually do
	Nausea and / or Vomiting	[0] No<br>[1] Yes	Approximate duration (in days)	Seriousness today:<br>[1] I could do everything that I usually do<br>[2] I could not do some of what I usually do<br>[3] I could not do most of what I usually do
	Painful muscles and joints	[0] No<br>[1] Yes	Approximate duration (in days)	Seriousness today:<br>[1] I could do everything that I usually do<br>[2] I could not do some of what I usually do<br>[3] I could not do most of what I usually do
	Loss of smell	[0] No<br>[1] Yes	Approximate duration (in days)	Seriousness today:<br>[1] I could do everything that I usually do<br>[2] I could not do some of what I usually do<br>[3] I could not do most of what I usually do
	Tiredness and fatigue	[0] No<br>[1] Yes	Approximate duration (in days)	Seriousness today:<br>[1] I could do everything that I usually do<br>[2] I could not do some of what I usually do<br>[3] I could not do most of what I usually do
	Loss of taste	[0] No<br>[1] Yes	Approximate duration (in days)	Seriousness today:<br>[1] I could do everything that I usually do<br>[2] I could not do some of what I usually do<br>[3] I could not do most of what I usually do
	Chills	[0] No<br>[1] Yes	Approximate duration (in days)	Seriousness today:<br>[1] I could do everything that I usually do<br>[2] I could not do some of what I usually do<br>[3] I could not do most of what I usually do
	Headache	[0] No<br>[1] Yes	Approximate duration (in days)	Seriousness today:<br>[1] I could do everything that I usually do

				[2] I could not do some of what I usually do<br>[3] I could not do most of what I usually do
	Irritability/ confusion	[0] No<br>[1] Yes	Approximate duration (in days)	Seriousness today:<br>[1] I could do everything that I usually do<br>[2] I could not do some of what I usually do<br>[3] I could not do most of what I usually do
	General weakness	[0] No<br>[1] Yes	Approximate duration (in days)	Seriousness today:<br>[1] I could do everything that I usually do<br>[2] I could not do some of what I usually do<br>[3] I could not do most of what I usually do
	Skin rash	[0] No<br>[1] Yes	Approximate duration (in days)	Seriousness today:<br>[1] I could do everything that I usually do<br>[2] I could not do some of what I usually do<br>[3] I could not do most of what I usually do
25	When did your symptoms first present?		dd/mm/yyyy	
26	Have you been diagnosed with COVID-19 before?		[0] No<br>[1] Yes	
27	If yes, how many times?			
28	Which was your most recent time? Give an approximate date of diagnosis		dd/mm/yyyy	
29	Have you been vaccinated?		[0] No<br>[1] Yes	
30	If you have not been vaccinated, we would be interested to know why not?			[1] It is a choice and the learner or learner's parent/primary caregiver choose not too<br>[2] No time<br>[3] In general, the learner's family is against all vaccinations<br>[4] No expected benefit (vaccination does not work at all or not sufficiently)<br>[5] The learner or learner's parent/primary caregiver does not trust the vaccine manufacturing companies<br>[6] The learner or learner's parent/primary caregiver does not trust the government's ability to roll out a safe vaccine.

		[7] The learner or learner’s parent/primary caregiver wants to wait until there is more knowledge<br>[8] The learner or learner’s parent/primary caregiver fear the side effects, safety and effectiveness of vaccinations<br>[9] Due to the learner or learner’s parnet/primary caregiver religious or cultural beliefs<br>[10] The learner is afraid of needles<br>[11] The learner had COVID-19, so I do not consider the vaccination necessary<br>[13] The learner’s parent/primary caregiver just won’t let them take it.<br>[14] Due to the learner’s medical condition<br>[15] Other<br>If other, please specify<br>*Please provide space to specify.*<br><br>*More than 1 answer allowed.*
31	If yes, when did you receive your first dose? Give an approximate date	dd/mm/yyyy
32	Which vaccine did you receive?	[1] Pfizer<br>[2] CoronaVac<br>[3] Other (specify)<br>[4] Don’t know
33	How many doses did you receive?<br>*Circle the correct answer*	Pfizer 0 1 2 3<br>CoronaVac 0 1 2 3<br>Other (specify) 0 1 2 3
34	Date of last (most recent dose)	dd/mm/yyyy

35	Do you have any of the following chronic illnesses:			
	HIV<br>[0] No<br>[1] Yes	Current TB<br>[0] No<br>[1] Yes	Chr. Kidney Disease<br>[0] No<br>[1] Yes	Chronic Liver Disease<br>[0] No<br>[1] Yes
	Neurological/neuromuscular Disease<br>[0] No<br>[1] Yes	Diabetes Mellitus (high blood sugar)<br>[0] No<br>[1] Yes	Heart disease<br>[0] No<br>[1] Yes	Cancer<br>[0] No<br>[1] Yes
	Prior TB infection<br>[0] No<br>[1] Yes	Hypertension (high blood pressure)<br>[0] No<br>[1] Yes	Asthma (Difficulty breathing)<br>[0] No<br>[1] Yes	Chr. Lung Disease/ COPD<br>[0] No<br>[1] Yes

	Rheumatologically (disease of the joints and muscles)<br>[0] No<br>[1] Yes	Obesity/Overweight<br>[0] No<br>[1] Yes	Autoimmune Disease (not HIV. A disease whereby our immune system starts attacking our own tissues or organs e.g., SLE)<br>[0] No<br>[1] Yes	Other 1: Details<br>Other 2: Details:<br>Other 3: Details:<br>Other 4: Details
36	Are you a smoker?		[0] No<br>[1] Yes	

37	**What non pharmaceutical measures do you currently use to prevent COVID-19?**			
	Masks in public places e.g., buses and taxis<br>[0] No<br>[1] Yes – always<br>[2] Yes - sometimes	Sanitizing<br>[0] No<br>[1] Yes – always<br>[2] Yes - sometimes	Masks in the school setting or workplace?<br>[0] No<br>[1] Yes – always<br>[2] Yes - sometimes	Distancing- - more than 1.5 meters away in the school setting or workplace?<br>[0] No<br>[1] Yes – always<br>[2] Yes - sometimes
	Avoiding social gatherings/outings<br>[0] No<br>[1] Yes – always<br>[2] Yes - sometimes	Avoiding weddings<br>[0] No<br>[1] Yes – always<br>[2] Yes - sometimes	Avoiding funerals<br>[0] No<br>[1] Yes – always<br>[2] Yes - sometimes	
**Close Contact: Specimen collection**				
38	Date of specimen collection?		*dd/mm/yyyy*	
39	Was a nasal swab collected for Rapid COVID-19 antigen POC test?		[1] Yes<br>[2] No<br>[99] Not applicable (N/A)	
40	If yes, what was the name of the test?			
41	What was the result?		[1] Positive<br>[2] Negative<br>[3] Indeterminant<br>[99] N/A	
42	Was a nasal swab collected for RT-PCR?		[1] Yes<br>[2] No<br>[99] N/A	

43	Was blood collected for DBS?	[1] Yes<br>[2] No<br>[99] N/A
