# Supplementary material for: SARS-CoV-2 Infections in a Triad of Primary School Learners (Grades 1-7), Their Parents, and Teachers in KwaZulu-Natal, South Africa: Protocol for a Cross-Sectional and Nested Case-Cohort Study
Source: JMIR Res Protoc. 2024 Dec 19;13:e52713. doi: 10.2196/52713 (PMC11695960; doi:10.2196/52713)
Supplement: Multimedia Appendix 6 [file resprot_v13i1e52713_app6.pdf]

COKIDSS NESTED CASE COHORT SUB STUDY:
eCRF FOR SUB-STUDY

Instructions:<br>*Study staff to follow all instructions in italics.*<br>*CRF questions to be repeated on RedCap at 30-days, 3-months and 6-months post COVID-19 diagnosis (n=4 visits).*		
1	Visit code	
2	Research staff ID<br>*This refers to the staff member assisting the participant to complete this CRF.*	
3	Do you have an SA ID or passport?	
4	What is the participants SA ID number or passport?	
5	Study unique identifier<br>*The RA will obtain the participant's unique identifier from the link log that was completed in the cross-sectional and/or studies.*	
6	Re-enter study unique identifier	*RA to complete.*
7	Today's date	dd/mm/yyyy
8	Is the participant a learner in grade 1-7, a parent/primary caregiver or a teacher?	[1] Learner in grade 1-7<br>[2] Parent/primary caregiver<br>[3] Teacher
9	What is the name of the school you attend (Learner or Teacher)?<br>OR<br>What is the name of the school your child attends (Parent/primary caregiver)?	
10	What year are you in at school (Learner)?<br>OR<br>What year is your child in at school (Parent/primary caregiver)?<br>OR<br>What year do you teach (Teacher)?	[1] Grade 1<br>[2] Grade 2<br>[3] Grade 3<br>[4] Grade 4<br>[5] Grade 5<br>[6] Grade 6<br>[7] Grade 7
11	What is the name of the learner or teacher'sclass?<br>*Class ID to be CODIFIED.*	

**Acute COVID-19 infection (within 10 days post diagnosis)**			
12	Are you currently feeling sick?		[0] No<br>[1] Yes
13	Do you have any of the following signs/symptoms now? If yes, indicate which signs/symptoms are currently present, and indicate approximate **duration and severity.**		*Use the format dd/mm/yyyy for date and days for duration*
Cough	[0] No	Approximate	Severity of

	[1] Yes	duration (in days)	symptoms today:<br>[1] I could do everything that I usually do<br>[2] I could not do some of what I usually do<br>[3] I could not do some of what I usually do most of what I usually do
Sore Throat	[0] No<br>[1] Yes	Approximate duration (in days)	Severity of symptoms today:<br>[1] I could do everything that I usually do<br>[2] I could not do some of what I usually do<br>[3] I could not do some of what I usually do most of what I usually do
Fever	[0] No<br>[1] Yes	Approximate duration (in days)	Severity of symptoms today:<br>[1] I could do everything that I usually do<br>[2] I could not do some of what I usually do<br>[3] I could not do some of what I usually do most of what I usually do
Body ache	[0] No<br>[1] Yes	Approximate duration (in days)	Severity of symptoms today:<br>[1] I could do everything that I usually do<br>[2] I could not do some of what I usually do

			[3] I could not do some of what I usually do most of what I usually do
Diarrhea	[0] No<br>[1] Yes	Approximate duration (in days)	Severity of symptoms today:<br>[1] I could do everything that I usually do<br>[2] I could not do some of what I usually do<br>[3] I could not do some of what I usually do most of what I usually do
Nausea/vomiting	[0] No<br>[1] Yes	Approximate duration (in days)	Severity of symptoms today:<br>[1] I could do everything that I usually do<br>[2] I could not do some of what I usually do<br>[3] I could not do some of what I usually do most of what I usually do
Painful muscle and joints	[0] No<br>[1] Yes	Approximate duration (in days)	Severity of symptoms today:<br>[1] I could do everything that I usually do<br>[2] I could not do some of what I usually do<br>[3] I could not do some of what I usually do most of what I usually do
Loss of smell	[0] No<br>[1] Yes	Approximate duration (in days)	Severity of symptoms today:<br>[1] I could do everything that

			I usually do<br>[2] I could not do some of what I usually do<br>[3] I could not do some of what I usually do most of what I usually do
Loss of taste	[0] No<br>[1] Yes	Approximate duration (in days)	Severity of symptoms today:<br>[1] I could do everything that I usually do<br>[2] I could not do some of what I usually do<br>[3] I could not do some of what I usually do most of what I usually do
Tiredness and fatigue	[0] No<br>[1] Yes	Approximate duration (in days)	Severity of symptoms today:<br>[1] I could do everything that I usually do<br>[2] I could not do some of what I usually do<br>[3] I could not do some of what I usually do most of what I usually do
Chills	[0] No<br>[1] Yes	**Approximate duration (in days)**	Severity of symptoms today:<br>[1] I could do everything that I usually do<br>[2] I could not do some of what I usually do<br>[3] I could not do some of what I

			usually do most of what I usually do
Headache	[0] No<br>[1] Yes	Approximate duration (in days)	Severity of symptoms today:<br>[1] I could do everything that I usually do<br>[2] I could not do some of what I usually do<br>[3] I could not do some of what I usually do most of what I usually do
Irritability or confusion	[0] No<br>[1] Yes	Approximate duration (in days)	Seriousness today:<br>[1] I could do everything that I usually do<br>[2] I could not do some of what I usually do<br>[3] I could not do some of what I usually do most of what I usually do
General weakness	[0] No<br>[1] Yes	Approximate duration (in days)	Severity of symptoms today:<br>[1] I could do everything that I usually do<br>[2] I could not do some of what I usually do<br>[3] I could not do some of what I usually do most of what I usually do
Skin rash	[0] No<br>[1] Yes	**Approximate duration (in days)**	Severity of symptoms today:<br>[1] I could do everything that I usually do<br>[2] I could not

			do some of what I usually do<br>[3] I could not do some of what I usually do most of what I usually do

14	When did your symptoms (signs) first present?	dd/mm/yyyy
15	In the last month, have you had close contact with any of the following people:<br>*Note:Close contact means you ate with or was in this person's company. They were close together for at least 15 minutes*	A suspected COVID-19 patient [1] Yes…[0] No<br>A confirmed COVID-19 patient [1] Yes…[0] No<br>Someone with the "flu" or "cold" [1] Yes…[0] No
16	If yes for any of the above, what setting was the contact<br>*(Please select the answer that applies)*	[1] Healthcare setting<br>[2] Family setting<br>[3] School setting<br>[4] Public transport setting<br>[5] Other<br>*If other, please specify.*
17	Did you quarantine after the contact?	[0] No<br>[1] Yes
18	If yes, for how long did you quarantine?	days
19	If no, how many contacts have you had since that time?	
**Close contact details**<br>***(****In RedCap, this section must be repeated to allow for up to 6 contacts to be inserted)*		
	Close Contact # 1	
20	Is the close contact a child or adult?	[1] 6-12 years of age<br>[2] >12 – 17 year of age<br>[3] ≥18 years of age
21	Address of close contact?	
22	Contact Number<br>*10 digits*	
	Close Contact # 2	
23	Is the close contact a child or adult?	[1] 6-12 years of age<br>[2] >12 – 17 year of age<br>[3] ≥18 years of age
24	Address of close contact?	
25	Contact Number<br>*10 digits*	
	Close Contact # 3	
26	Is the close contact a child or adult?	[1] 6-12 years of age<br>[2] >12 – 17 year of age

		[3] ≥18 years of age
27	Address of close contact?	
28	Contact Number<br>*10 digits*	
	Close Contact # 4	
29	Is the close contact a child or adult?	[1] 6-12 years of age<br>[2] >12 – 17 year of age<br>[3] ≥18 years of age
30	Address of close contact?	
31	Contact Number<br>*10 digits*	
	Close Contact # 5	
32	Is the close contact a child or adult?	[1] 6-12 years of age<br>[2] >12 – 17 year of age<br>[3] ≥18 years of age
33	Address of close contact?	
34	Contact Number<br>*10 digits*	
	Close Contact # 6	
35	Is the close contact a child or adult?	[1] 6-12 years of age<br>[2] >12 – 17 year of age<br>[3] ≥18 years of age
36	Address of close contact?	
37	Contact Number<br>*10 digits*	
**Previous history of COVID-19**		
38	Have you been diagnosed with COVID-19 before?	[0] No<br>[1] Yes
39	If yes, how many times?	
40	Which was their most recent time? Give an approximate date of diagnosis	dd/mm/yyyy
41	Over the past 2 years have you felt sick or more tired, or had headaches or lost his/her taste or had COVID-19-like symptoms (signs) for 28-days or longer?	[0] No<br>[1] Yes

**Long COVID**<br>*(Only answer question if answer to question 41 was yes)*	
42	Tell us which signs/symptoms were present for more than 28-days.
Fatigue/tiredness<br>[0] No<br>[1] Yes	
Stuffy/runny nose<br>[0] No<br>[1] Yes	Chest tightness<br>[0] No<br>[1] Yes

Chest pain<br>[0] No<br>[1] Yes	Cough<br>[0] No<br>[1] Yes
Wheezing<br>[0] No<br>[1] Yes	Sore throat<br>[0] No<br>[1] Yes
Muscle ache<br>[0] No<br>[1] Yes	Joint pain/swelling<br>[0] No<br>[1] Yes
Headache<br>[0] No<br>[1] Yes	Dizziness<br>[0] No<br>[1] Yes
Altered sense of taste (change in taste)<br>[0] No<br>[1] Yes	Altered sense of smell<br>[0] No<br>[1] Yes
Difficulty concentrating (focusing)<br>[0] No<br>[1] Yes	Sleep disorders<br>[0] No<br>[1] Yes
Mood alterations<br>[0] No<br>[1] Yes	Cognitive dysfunction<br>(loss of memory or attentiveness)<br>[0] No<br>[1] Yes
Sensorimotor symptoms (tingling in the toes / feet / legs / fingers / hands or twitching of muscles)<br>[0] No<br>[1] Yes	Increased need for sleep<br>[0] No<br>[1] Yes
Weight loss<br>[0] No<br>[1] Yes	Diarrhea<br>[0] No<br>[1] Yes
Stomach pain<br>[0] No<br>[1] Yes	Poor appetite<br>[0] No<br>[1] Yes
Constipation<br>[0] No<br>[1] Yes	Skin rash<br>[0] No<br>[1] Yes
Tachycardia<br>(fast heart rate)<br>[0] No<br>[1] Yes	Fever<br>[0] No<br>[1] Yes
Other<br>*If other, please provide space to specify*	

43	If, you have ticked at least one symptom (sign) above. Please select	[1] A doctor had to be seen because of this

	what applies:	[2] You had to stay away from school/work<br>*(If applicable answer question 28)*<br>[3] You had to be treated with medication<br>*(If applicable answer question 29)*<br>[4] You had to be hospitalized<br>*(If applicable answer question 30)*<br>[5] You have not done anything<br>[6] Other<br>*If other, please specify.______________________________*<br>*(multiple answers possible)*
44	How many days did you have to miss school/work because of any one or more of these symptoms?	______***(Please enter number in days)***
45	How many days did you have to be treated with medication because of any one or more of these symptoms?	______***(Please enter number in days)***
46	How many days did you have to spend in the hospital because of any one or more of these symptoms?	______***(Please enter number in days)***

**COVID-19 vaccination and vaccination history**<br>*These questions are applicable for participants that are eligible for the COVID-19 vaccination (≥ 12 years of age)*		
47	Have you been vaccinated against COVID-19?	[0] No<br>[1] Yes
48	If you have not been vaccinated, we would be interested to know why?	[1] It is a choice and I choose not too<br>[2] No time<br>[3] In general, my family is against all vaccinations<br>[4] No expected benefit (vaccination does not work at all or not enough)<br>[5] I do not trust the vaccine manufacturing companies<br>[6] I do not trust the government's ability to roll out a safe vaccine.<br>[7] I want to wait until there is more knowledge<br>[8]I fear the side effects, safety and effectiveness of vaccinations

		[9] Due to my religious or cultural beliefs<br>[10] I am afraid of needles<br>[11] I had COVID-19, so I do not consider the vaccination necessary<br>[14] Due to my medical condition<br>[15] Other<br>If other, please specify___________________<br>*Please provide space to specify.*<br><br>*More than 1 answer allowed.*
49	If yes, when did you receive your first dose? Give an approximate date	dd/mm/yyyy
50	Which vaccine did you receive?<br>(*Please tick the correct answer*)	see vaccine table below
51	Date of last dose?	dd/mm/yyyy
52	Do you have any of the following complications?<br>*(Please tick the correct answer)*	

	J&J	0	1	2	
	AstraZeneca	0	1	2	3
	CoronaVac	0	1	2	3
	Moderna	0	1	2	3
	Pfizer	0	1	2	3
	Other (please specify)	0	1	2	3

HIV<br>[0] No<br>[1] Yes	Current TB<br>[0] No<br>[1] Yes	Chronic Kidney Disease<br>[0] No<br>[1] Yes	Chronic Liver Disease<br>[0] No<br>[1] Yes
Neurological/neuromuscular disease<br>[0] No<br>[1] Yes	Diabetes Mellitus<br>[0] No<br>[1] Yes	Heart Disease<br>[0] No<br>[1] Yes	Cancer<br>[0] No<br>[1] Yes
Prior TB infection<br>[0] No<br>[1] Yes	Hypertension<br>[0] No<br>[1] Yes	Asthma<br>[0] No<br>[1] Yes	Chronic Lung Disease<br>[0] No<br>[1] Yes
Rheumatological disease<br>[0] No<br>[1] Yes	Obesity/overweight<br>[0] No<br>[1] Yes	Autoimmune disease (e.g., SLE)<br>[0] No<br>[1] Yes	Other 1: Details<br>Other 2: Details<br>Other 3: Details<br>Other 4: Details

53	Are you currently taking any of the following medications NOW:
Steroids<br>(e.g., Prednisone, cortisone)<br>[0] No<br>[1] Yes<br>[2] Prefer not to answer	Anti-inflammatories<br>(e.g., high dose aspirin, ibuprofen)<br>[0] No<br>[1] Yes<br>[2] Prefer not to answer
Anti-hypertensive<br>(blood pressure medication e.g., indapamide)<br>[0] No<br>[1] Yes<br>[2] Prefer not to answer	Chemotherapy<br>(cancer treatment)<br>[0] No<br>[1] Yes<br>[3] Prefer not to answer
Hormonal treatment<br>[0] No<br>[1] Yes<br>[2] Prefer not to answer	Antibiotics<br>(e.g., penicillin, amoxicillin)<br>[0] No<br>[1] Yes<br>[2] Prefer not to answer
ARV/ART<br>[0] No<br>[1] Yes<br>[2] Prefer not to answer	Bactrim prophylaxis<br>[0] No<br>[1] Yes<br>[2] Prefer not to answer
Aspirin / Warfarin / Heparin<br>[0] No<br>[1] Yes<br>[2] Prefer not to answer	TB Meds<br>[0] No<br>[1] Yes<br>[2] Prefer not to answer
Other 1: Details<br>Other 2: Details<br>Other 3: Details	

54	What non pharmaceutical measures does the learner currently use to prevent COVID-19?		
Masks in public places e.g., buses and taxis<br>[0] No<br>[1] Yes- always<br>[2] Yes - sometimes<br>[99] Not applicable (N/A)	Sanitizing<br>[0] No<br>[1] Yes- always<br>[2] Yes - sometimes<br>[99] N/A	Masks in the school setting or workplace?<br>[0] No<br>[1] Yes- always<br>[2] Yes - sometimes<br>[99] N/A	Distancing- - more than 1.5 meters away in the school setting or workplace?<br>[0] No<br>[1] Yes- always

			[2] Yes - sometimes<br>[99] N/A
Avoiding social gatherings/outings<br>[0] No<br>[1] Yes- always<br>[2] Yes - sometimes<br>[99] N/A	Avoiding weddings<br>[0] No<br>[1] Yes- always<br>[2] Yes - sometimes<br>[99] N/A	Avoiding funerals<br>[0] No<br>[1] Yes- always<br>[2] Yes - sometimes<br>[99] N/A	

**Specimen collection**<br>*This section is to be completed by study staff collecting the specimens from the participant*		
55	Date of specimen collection?	dd/mm/yyyy
56	Was a nasal swab collected for Rapid COVID-19 antigen POC test?	[0] No<br>[1] Yes<br>[99] N/A
57	If yes, what was the name of the test?	
58	What was the result?	[1] Positive<br>[2] Negative<br>[3] Indeterminant<br>[99] N/A
59	Was a nasal swab collected for RT-PCR?	[0] No<br>[1] Yes<br>[99] N/A
60	Was blood collected for immunology tests	[0] No<br>[1] Yes<br>[99] N/A
61	Was blood collected for Rapid COVID-19 antibody POC test?	[0] No<br>[1] Yes<br>[99] N/A
62	If yes, what was the name of the test?	[1] Orient gene<br>[2] Other, please specify____________________
63	What was the result?<br>*(more than one answer allowed)*	[1] Indeterminant<br>[2] IgM positive<br>[3] IgG positive
64	Was blood collected for DBS?	[0] No<br>[1] Yes<br>[99] N/A
