# Supplementary material for: SARS-CoV-2 Infections in a Triad of Primary School Learners (Grades 1-7), Their Parents, and Teachers in KwaZulu-Natal, South Africa: Protocol for a Cross-Sectional and Nested Case-Cohort Study
Source: JMIR Res Protoc. 2024 Dec 19;13:e52713. doi: 10.2196/52713 (PMC11695960; doi:10.2196/52713)
Supplement: Multimedia Appendix 7 [file resprot_v13i1e52713_app7.pdf]

Date: ____________

Name/ID: ________________________

RCADS-P-25

Relationship to Child: ______________________

Please put a circle around the word that shows how often each of these things happens for your child.

1. My child feels sad or empty	Never	Sometimes	Often	Always
2. My child worries when he/she thinks he/she has done poorly at something	Never	Sometimes	Often	Always
3. My child feels afraid of being alone at home	Never	Sometimes	Often	Always
4. Nothing is much fun for my child anymore	Never	Sometimes	Often	Always
5. My child worries that something awful will happen to someone in the family	Never	Sometimes	Often	Always
6. My child is afraid of being in crowded places (like shopping centers, the movies, buses, busy playgrounds)	Never	Sometimes	Often	Always
7. My child worries what other people think of him/her	Never	Sometimes	Often	Always
8. My child has trouble sleeping	Never	Sometimes	Often	Always
9. My child feels scared to sleep on his/her own	Never	Sometimes	Often	Always
10. My child has problems with his/her appetite	Never	Sometimes	Often	Always
11. My child suddenly becomes dizzy or faint when there is no reason for this	Never	Sometimes	Often	Always
12. My child has to do some things over and over again (like washing hands, cleaning, or putting things in a certain order)	Never	Sometimes	Often	Always
13. My child has no energy for things	Never	Sometimes	Often	Always
14. My child suddenly starts to tremble or shake when there is no reason for this	Never	Sometimes	Often	Always
15. My child cannot think clearly	Never	Sometimes	Often	Always
16. My child feels worthless	Never	Sometimes	Often	Always
17. My child has to think of special thoughts (like numbers or words) to stop bad things from happening	Never	Sometimes	Often	Always
18. My child thinks about death	Never	Sometimes	Often	Always
19. My child feels like he/she doesn’t want to move	Never	Sometimes	Often	Always
20. My child worries that he/she will suddenly get a scared feeling when there is nothing to be afraid of	Never	Sometimes	Often	Always
21. My child is tired a lot	Never	Sometimes	Often	Always
22. My child feels afraid that he/she will make a fool of him/herself in front of people	Never	Sometimes	Often	Always
23. My child has to do some things in just the right way to stop bad things from happening	Never	Sometimes	Often	Always
24. My child feels restless	Never	Sometimes	Often	Always
25. My child worries that something bad will happen to him/her	Never	Sometimes	Often	Always

© 1998 Bruce F. Chorpita and Susan H. Spence – For terms of use, see User’s Guide at www.childfirst.ucla.edu/resources/
