# Supplementary material for: SARS-CoV-2 Infections in a Triad of Primary School Learners (Grades 1-7), Their Parents, and Teachers in KwaZulu-Natal, South Africa: Protocol for a Cross-Sectional and Nested Case-Cohort Study
Source: JMIR Res Protoc. 2024 Dec 19;13:e52713. doi: 10.2196/52713 (PMC11695960; doi:10.2196/52713)
Supplement: Multimedia Appendix 8 [file resprot_v13i1e52713_app8.docx]

**Appendix 8**

**Detailed data collection**

Follow-up of participants will assess flu-like symptoms (onset, type, duration, severity) and SARS-CoV-2 infection test results outside this research study within the household; adherence to preventive measures and possible exposures (travels abroad, contact with confirmed SARS-CoV-2 cases, etc.) within the household and symptoms of long COVID if applicable.

School principals will also be asked to complete questionnaires during the cross-sectional survey and follow-up survey. The following information will be collected: total number of learners per school and school level; the number of learners and teachers in classes; preventive measures at school organizational, infrastructure and personnel levels. Socioeconomic status of the school will also be estimated at baseline from official statistics. All enrolled teachers at participating schools will complete a questionnaire. The survey will include questions covering socio-demographics; preventive measures being implemented; challenges and consistency with which they are implemented; facilitators for implementation; satisfaction with school communication and approach and occupational stress.

Participants will be referred to the DoH facilities, should the study team identify prevention and management of medical conditions including HIV/AIDS, STIs, TB and COVID-19 during the duration of the study as per institution’s memorandum of agreement with the South African DoH.

**Sample collection, testing procedures**

Table 5 summarizes the sample collection at various time points. Sample collection and testing will take place in a sufficiently large room, in small groups of participating learners, parents/guardians and teachers, with all necessary non-pharmaceutical interventions (NPIs) in place. All study staff will use WHO-recommended personal protective equipment (PPE).

First, study information will be provided, and the child’s identity, assent (8-<18 years of age, if applicable) and parental consent confirmed .

*COVID-19 antibody testing:* A SAHPRA approved point-of-care COVID-19 antibody test will be used to assess antibody prevalence. These tests require a fingerstick blood draw, and results can be provided on the same day.

*Dried Blood Spot (DBS) sample collection:* DBS samples will also be collected from all participants for future analyses when the finger-prick blood draw is performed for SARS-CoV-2 antibody testing or venous blood draw is conducted.

*Saliva sample collection and storage:* Saliva samples will be collected and stored from 10% of participants enrolled across the cross-sectional and follow-up surveys for future testing.

*COVID-19 antigen testing:* When the protocol team becomes aware of the first 30 participants (at least 20 children, preferably all children (n=30)) who test positive for SARS-CoV-2, through routine testing using SAHPRA approved point of care SARS-CoV-2 antigen test or a RT- PCR test, these 30 participants, now called the primary positives, will be offered enrolment into the nested case cohort sub study. A trained nurse will collect one (child) or two (adult) nasal swabs for SARS-CoV-2 antigen detection. Remnants of the nasal swab for RT-PCR testing will be sent for viral genome sequencing.

The primary positives will be contacted to identify up to 10 close contacts. These close contacts will be offered enrolment into the transmission dynamics investigation study and enrolled participants will be investigated for SARS-CoV-2 infection using a point-of-care antigen test and SARS-CoV-2 PCR test. Remnants of the SARS-CoV-2 PCR test nasal swab will be used for the viral genome sequencing.

In the nested case cohort sub-study, the primary positive participants will be followed-up for 6-months post-diagnosis. During follow-up, the nurse will collect venous blood for B- and T- cell response testing.

*Lab assays for exploratory sub study:*

*Whole genome sequencing:* Remnant specimens from participants with PCR-confirmed SARS-CoV-2, will be fed into the Network for Genomic Surveillance in SA (NGS-SA) for routine viral sequencing. Amplification and sequencing for viral lineages will be conducted per previously published protocols [1,2].

*T-Cell responses:* We will assess for Spike-specific responses of SARS-CoV-2 convalescent learners/parents. We will describe the effect of prior COVID-19 infection on T cell responses, by measuring intracellular cytokine production (IFN-g, TNF-a and IL-2) in response to PBMCs that are stimulated by peptides covering the wild-type virus spike (PepTivator® SARS-CoV-2 Prot_S+, Miltenyi Biotec).

*Binding antibody responses:* To evaluate binding antibody responses, we will use antibody mediated binding of SARS-CoV-2 antigens by using an enzyme linked immunosorbent assay (ELISA), the methodology of which has been published elsewhere [3].

*Neutralizing antibody responses:* We will assess for neutralizing antibody responses in SARS-CoV-2 convalescent learners/parents using a SARS-CoV-2 pseudovirus neutralization assay [4].

*Storage conditions and protection of biospecimens*

Dried blood spot (DBS) and saliva samples will be collected from all participants and 10% of participants enrolled in the study, respectively for future studies. All proposed research studies using these samples will be reviewed by the SAMRC, HREC in SA. The samples will be stored for a maximum of 15 years at the HIDRU, SAMRC, biorepository in -80°C freezers. The biorepository is registered on the Division of AIDS (DAIDS) oversight system and currently houses samples for both National Institute of Health [NIH] and Non-NIH studies. The LDMS program developed by Frontier science is used by the biorepository as a storage module [5]. The biorepository monitors temperature manually and through a continuous temperature monitoring system called Omniflex. The system is used to monitor the temperature of the freezers and storage areas at the biorepository. Two back-up generators are installed to keep the biorepository operating in the event of a loss of electrical supply. Alarms are sent out indicate when temperature deviations occur outside of the referenced range of the storage area. Alarms and notifications are sent to staff via short messaging system (SMS) and email to notify them of any temperature deviations which are attended to by the staff. Entry into the Biorepository is access controlled.

**References**

1. Baggio S, L’Huillier AG, Yerly S, Bellon M, Wagner N, Rohr M, et al. SARS-CoV-2 viral load in the upper respiratory tract of children and adults with early acute COVID-19. Clinical Infectious Diseases 2020. https://doi.org/[10.1093/cid/ciaa1157](https://doi.org/10.1093%2Fcid%2Fciaa1157)
2. Tegally H, Wilkinson E, Marta Giovanetti M, Iranzadeh A, Fonseca V, Giandhari J, et al (2020). Emergence and rapid spread of a new severe acute respiratory syndrome-related coronavirus 2 (SARS-CoV-2) lineage with multiple spike mutations in South Africa. medRxiv 2020.12.21.20248640. https://doi.org/10.1101/2020.12.21.20248640
3. Moyo-Gwete T, Madzivhandila M, Makhado Z, Ayres F, Mhlanga D, Oosthuysen B, et al. Cross-reactive neutralizing antibody responses elicited by SARS-CoV-2 501Y.V2 (B.1.351). New England Journal of Medicine 2021; 384:2161-2163.
   https://doi.org/10.1056/NEJMc2104192
4. Wibmer CK, Ayres F, Hermanus T, Madzivhandila M, Kgagudi P, Oosthuysen B et al. SARS-CoV-2 501Y.V2 escapes neutralization by South African COVID-19 donor plasma. Nature Medicine 2021; 27: 622-625. <https://doi.org/10.1038/s41591-021-01285-x>
5. Laboratory Data Management System, Frontier Science Foundation

<http://www.fstrf.org/ldms/>. Accessed on 08 September 2024.
