# Supplementary material for: SARS-CoV-2 Infections in a Triad of Primary School Learners (Grades 1-7), Their Parents, and Teachers in KwaZulu-Natal, South Africa: Protocol for a Cross-Sectional and Nested Case-Cohort Study
Source: JMIR Res Protoc. 2024 Dec 19;13:e52713. doi: 10.2196/52713 (PMC11695960; doi:10.2196/52713)
Supplement: Multimedia Appendix 9 [file resprot_v13i1e52713_app9.docx]

**Appendix 9.** Study activities and timelines.

|  | May 2023 | Jun 2023 | Jul 2023 | Aug 2023 | Sep 2023 | Oct 2023 | Nov 2023 | Dec 2023 | Jan 2024 | Feb 2024 | Mar 2024 | Apr 2024 | May 2024 | Jun 2024 | Jul 2024 | Aug 2024 | Sep 2024 | Oct 2024 | Nov 2024 | Dec 2024 |
| --- | --- | --- | --- | --- | --- | --- | --- | --- | --- | --- | --- | --- | --- | --- | --- | --- | --- | --- | --- | --- |
| Recruitment and enrolment:  Cross-sectional survey | ✓ | ✓ | ✓ | ✓ |  |  |  |  |  |  |  |  |  |  |  |  |  |  |  |  |
| Follow-up:  Follow-up survey |  |  |  |  |  | ✓ |  |  |  |  |  |  |  |  |  |  |  |  |  |  |
| Nested case-cohort substudy  Recruitment and follow-up |  |  |  |  |  |  | ✓ | ✓ | ✓ | ✓ | ✓ | ✓ | ✓ | ✓ | ✓ | ✓ | ✓ | ✓ |  |  |
| Data clean up and analysis  Cross-sectional survey |  |  |  |  |  |  |  |  |  |  |  | ✓ | ✓ | ✓ |  |  |  |  |  |  |
| Data clean up and analysis  Follow-up survey and nested case-cohort substudy |  |  |  |  |  |  |  |  |  |  |  |  |  |  | ✓ | ✓ | ✓ |  |  |  |
| Write-up and dissemination |  |  |  |  |  |  |  |  |  |  |  |  |  |  |  |  |  | ✓ | ✓ | ✓ |
